# Leveraging machine learning and network biology approaches to predict brain gene expression from blood transcriptomes

**DOI:** 10.1093/gigascience/giag058

**Published:** 2026-05-18

**Authors:** Cigdem Sevim Bayrak, Qi Zeng, Marjan Ilkov, Scott J Russo, Minghui Wang, Bin Zhang

**Affiliations:** Department of Genetics and Genomic Sciences, Icahn School of Medicine at Mount Sinai, One Gustave L. Levy Place, Box 1498 New York, NY 10029, USA; Mount Sinai Center for Transformative Disease Modeling, Icahn School of Medicine at Mount Sinai, One Gustave L. Levy Place, Box 1498 New York, NY 10029, USA; Department of Genetics and Genomic Sciences, Icahn School of Medicine at Mount Sinai, One Gustave L. Levy Place, Box 1498 New York, NY 10029, USA; Mount Sinai Center for Transformative Disease Modeling, Icahn School of Medicine at Mount Sinai, One Gustave L. Levy Place, Box 1498 New York, NY 10029, USA; Department of Genetics and Genomic Sciences, Icahn School of Medicine at Mount Sinai, One Gustave L. Levy Place, Box 1498 New York, NY 10029, USA; Mount Sinai Center for Transformative Disease Modeling, Icahn School of Medicine at Mount Sinai, One Gustave L. Levy Place, Box 1498 New York, NY 10029, USA; Friedman Brain Institute, Icahn School of Medicine at Mount Sinai, One Gustave L. Levy Place, Box 1498 New York, NY 10029, USA; Nash Family Department of Neuroscience, Icahn School of Medicine at Mount Sinai, One Gustave L. Levy Place, Box 1498 New York, NY 10029, USA; Brain and Body Research Institute, Icahn School of Medicine at Mount Sinai, One Gustave L. Levy Place, Box 1498 New York, NY 10029, USA; Department of Genetics and Genomic Sciences, Icahn School of Medicine at Mount Sinai, One Gustave L. Levy Place, Box 1498 New York, NY 10029, USA; Mount Sinai Center for Transformative Disease Modeling, Icahn School of Medicine at Mount Sinai, One Gustave L. Levy Place, Box 1498 New York, NY 10029, USA; Department of Genetics and Genomic Sciences, Icahn School of Medicine at Mount Sinai, One Gustave L. Levy Place, Box 1498 New York, NY 10029, USA; Mount Sinai Center for Transformative Disease Modeling, Icahn School of Medicine at Mount Sinai, One Gustave L. Levy Place, Box 1498 New York, NY 10029, USA

**Keywords:** blood-based biomarkers, transcriptomics, gene co-expression networks, machine learning

## Abstract

**Background:**

Blood-based biomarkers offer a promising non-invasive strategy for detecting disease-related changes and monitoring tissue and organ health, including brain function. While recent studies have leveraged blood transcriptomic data to predict gene expression in the brain, existing models generally suffer from poor accuracy, limiting their translational utility.

**Findings:**

We present an integrative prediction system (IPS) that combines machine learning with network biology to predict region-specific brain gene expression from blood transcriptomic data. Our framework integrates global blood transcriptomic signals, co-expression network features, and inter-tissue gene–gene interaction data linking blood genes to their target genes in the brain. Applied to the Genotype-Tissue Expression cohort, IPS substantially outperforms existing approaches in both the number and accuracy of brain genes that can be reliably predicted from blood. Notably, immune-related blood genes emerged as key contributors to model performance, underscoring the systematic interplay between peripheral immune signaling and central nervous system.

**Conclusions:**

These findings highlight the potential of blood-based transcriptomic models as scalable, non-invasive tools for studying brain function and developing diagnostic and prognostic biomarkers for neurological and psychiatric disorders.

## Graphical Abstract

**Graphical Abstract:**
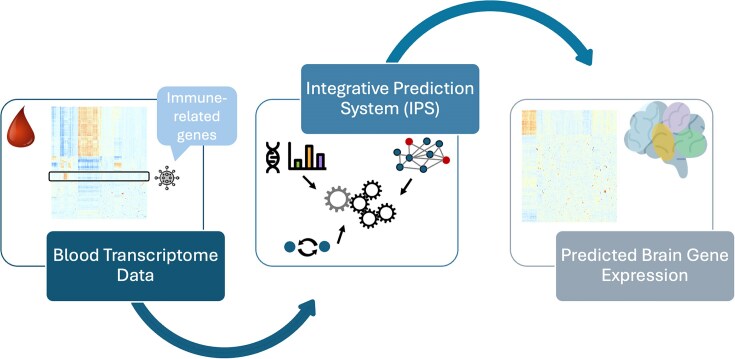

## Introduction

Blood biomarkers are emerging as a minimally invasive approach to investigate both neurodegenerative disease (NDD) pathology and normal brain function. NDDs, including Alzheimer’s disease (AD) and Parkinson’s disease (PD), affect millions globally and account for ~15% of the population. With the global population aging, the burden of these disorders is expected to rise significantly [1]. Early and precise diagnosis is essential for effective prevention and treatment but remains challenging in clinical settings. While neuroimaging and cerebrospinal fluid biomarkers have advanced in vivo characterization of disease processes, their use is limited by cost, limited accessibility, and invasiveness [2]. Blood-based biomarkers offer a minimally invasive, cost-effective alternative for detecting disease-related changes and monitoring brain health across the lifespan [3], with the potential to transform both clinical practice and research into normal and pathological brain aging.

The brain, the most complex organ in the human body, consists of billions of neurons forming trillions of connections with each other [4]. Characterizing the brain transcriptome is essential for understanding the molecular mechanisms that underpin neurological disorders. However, the limited availability of human brain tissue samples presents a significant challenge [5]. While gene expression patterns in the brain are largely consistent across individuals, distinct transcriptional profiles are observed across different tissues, with tissue-specific characteristics primarily determined by a select group of genes [6, 7]. Notably, studies have shown a strong correlation between gene expression profiles in the brain and blood [8], with co-expression networks of genes maintained across both tissues [9]. This strong correlation, along with the ease of access and cost-effectiveness of blood samples, makes the blood transcriptome a valuable resource for studying neurological disorders. Hence, many research efforts focus on utilizing patient blood samples to identify gene signatures associated with these conditions [10–12]. Predicting brain gene expression using blood transcriptome data could significantly enhance our understanding of brain-specific gene activity and disease-related changes. Moreover, incorporating transcriptional profiles from various tissues could further improve the accuracy of predictions regarding gene expression in the brain.

Recent studies have leveraged blood transcriptome data to develop generalized, transcriptome-wide models for predicting brain expression data [13–15]. Among these, BrainGENIE is a computational framework that uses peripheral blood gene expression profiles to impute brain tissue-specific expression levels across multiple brain regions. BrainGENIE employs principal component analysis (PCA) for feature selection to address the high dimensionality of the data, and applies regression-based modeling to predict brain expression patterns. While such approaches have demonstrated improved performance over genotype-based prediction models for subsets of genes, their overall predictive accuracy remains limited. More advanced and targeted approaches are required for more accurately capturing the complex, tissue-specific interactions between peripheral and brain gene expression. To improve gene-specific prediction performance, we propose an integrative prediction system (IPS), which leverages a diverse set of feature selection algorithms to model the complex, tissue-specific relationships, rather than relying on a singular, genome-wide predictive framework. In this study, we apply both (i) unsupervised feature selection, where features are selected without reference to the target brain gene, and (ii) supervised feature selection, where features are selected based on their relationship with the target brain gene. Leveraging paired blood and brain expression data from the Genotype-Tissue Expression (GTEx) dataset (v.8) [16], this study seeks to enhance the accuracy of gene-specific predictions across 12 brain tissues by integrating features from both blood transcriptomic data and co-expression network models derived from the Multiscale Embedded Gene co-Expression Network Analysis (MEGENA) network [17]. Our comparison of different feature sets and selection strategies demonstrates that different approaches predict distinct gene sets, highlighting the importance of using complementary methods to improve gene-specific predictions. This approach lays the groundwork for more precise, tissue-specific biomarkers and better understanding of gene expression dynamics across tissues. Prediction models based on peripheral tissue could extend their applicability beyond brain tissue and NDDs. When applied to other tissues, such as the heart and lungs, these models have the potential to provide valuable diagnostic and prognostic insights for a wide range of conditions.

## Materials and methods

**Figure 1:**
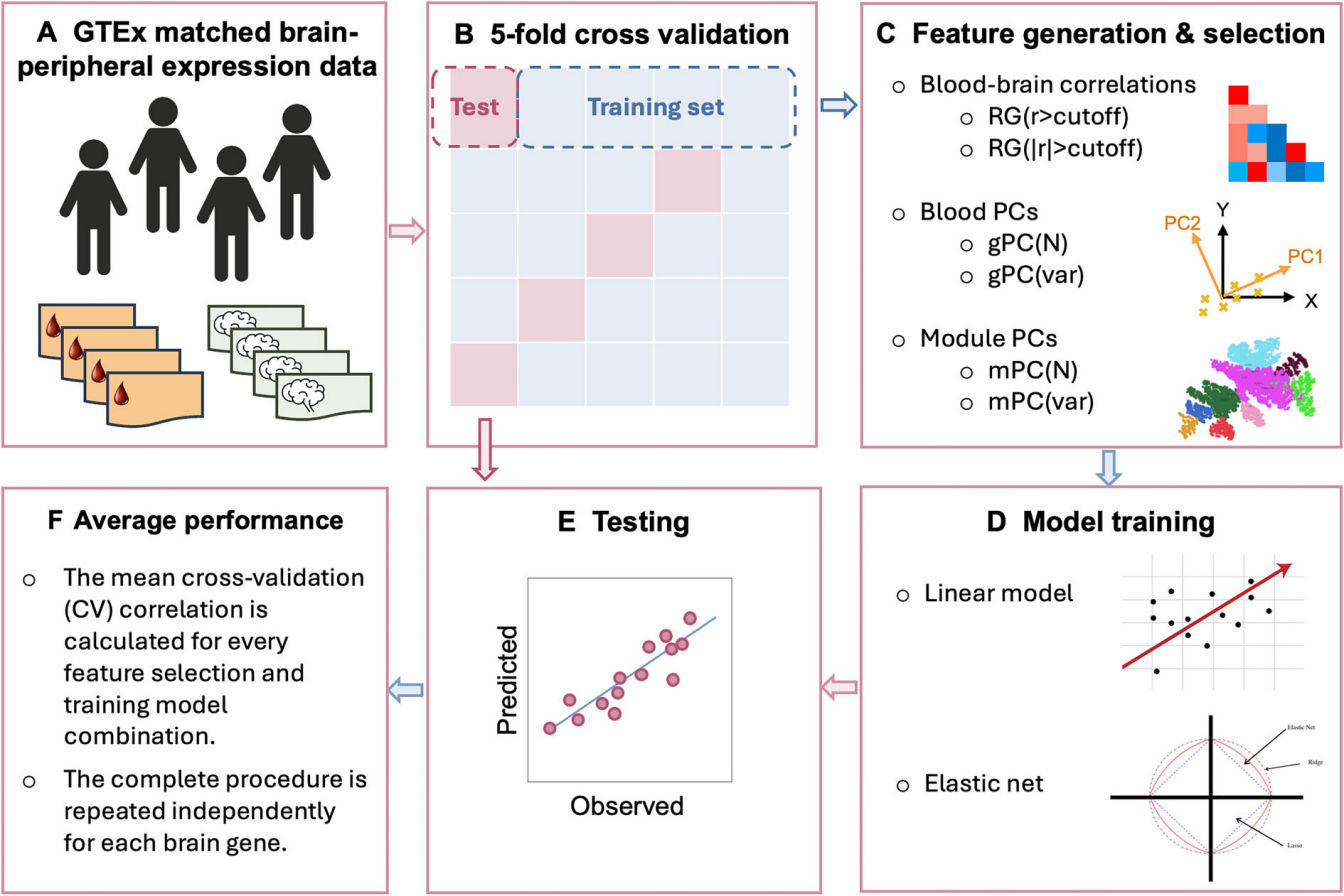
General framework of the IPS for predicting brain gene expression using paired blood and brain transcriptome data from the GTEx dataset. The prediction models have been trained on paired blood and brain expression data from the GTEx dataset (A) for each brain tissue via 5-fold cross-validation (B). Predictive features have been generated by (i) taking the top principal components, (ii) taking the highly correlated features with the target brain gene, and (iii) taking the top principal components of the MEGENA modules of blood tissue (C). For training (D), linear regression and elastic models were used. The model prediction accuracy (E) was estimated by calculating the average correlation between predicted and observed brain gene expression values over 5 folds (F). Icon by Selman Design, via Icon-Icons licensed under CC BY 4.0.

A general workflow of this study is shown in Fig. 1, and the details are explained below.

### Dataset

**Figure 2:**
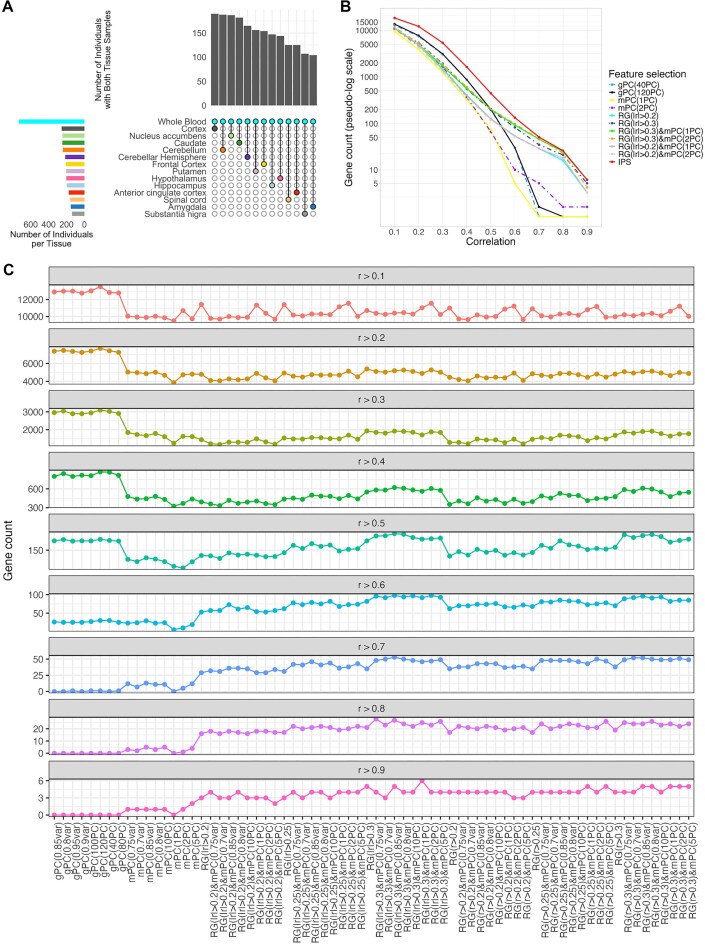
Sample overlap and gene prediction performance across feature selection methods. (A) Upset plot showing the number of individuals with paired whole blood and brain tissue samples in GTEx. The plot indicates that more than 100 subjects have both whole blood and brain tissue samples across all brain regions. (B) Number of genes predicted at various accuracy cutoffs using different feature selection methods. The *x*-axis shows the average correlation between the actual and predicted frontal cortex expression derived from blood tissue. gPC(.) represents the features selected by PCA from the blood transcriptome data, mPC(.) represents the module features selected by PCA, and RG(.) represents the features selected based on correlation with target brain gene. IPS indicates the number of genes when best feature selection method was considered per gene. (C) Number of predicted genes by different feature selection strategies. The *y*-axis indicates the number of predicted genes in the frontal cortex. Each plot shows the number of predicted genes at varying levels of prediction accuracy, measured by the average cross-validation correlation, *r*.

We have downloaded the raw count of RNA-seq data from the GTEx (v8) database and normalized using trimmed mean of *M*-values normalization (TMM) method to adjust for sequencing library size difference [18]. The normalized gene expressions were then log2 transformed. We applied a linear model to adjust for the covariates, including “SMCENTER” (collection sites), “SMRIN” (RNA integrity), “SMTSISCH” (ischemic time), “SMEXNCRT” (exonic rate), “SMRRNART” (rRNA rate), “SMNTERRT” (intergenic rate), and “SEX” (gender) and used the residuals from the regression model for downstream analysis. Next, we have prepared paired blood and brain transcriptome data for each brain tissue. Figure 2A shows the number of individuals with paired blood-brain data across different brain tissues. A detailed summary of the corresponding sample characteristics is provided in Supplementary Table S1, including the preservation method, number of individuals with paired whole blood samples, mean age, and sex distribution for each tissue.

### Network models

In order to identify groups of closely co-expressed genes, we have generated tissue-specific co-expression networks utilizing the MEGENA [17] R package. The gene expression matrix was permuted (*n* = 10) across the samples to calculate the false-positive rate and the corresponding false discovery rate (FDR) for each correlation coefficient cutoff. An FDR threshold of 0.05 was then applied to determine the correlation coefficient cutoff that effectively filtered out insignificant correlations. The significant gene pairs were sorted by their absolute Pearson correlation coefficients. These sorted gene pairs were sequentially examined to determine if they could be placed on a 3-dimensional topological sphere without intersecting other edges, a process known as the planarity test. Multiscale clustering analysis (MCA) was applied on the resulting co-expression network, planar filtered network, to identify network clusters (e.g., gene modules) at various compactness resolutions. MCA divides the parent module into child modules by searching for an optimal partition based on Newman’s modularity. Multiscale hub analysis was then performed by to identify nodes with significantly higher network connectivity compared to the randomly permuted planar networks (*P* < 0.05). Finally, PCA was applied on each module to determine module features.

### Feature selection

Unsupervised feature selection: To reduce the dimensionality of the blood expression training data, PCA was applied using the prcomp() R function. We evaluated different numbers of principal components (PCs, e.g., 40, 80, 100, and 120 PCs) and explored various thresholds for the percentage of explained variance (e.g., 80, 85, 90, and 95%).

Supervised feature selection: To select subsets of features that are highly correlated with the target brain gene expression data, we have applied correlation-based feature selection by Pearson’s correlation measure using the cor() R function. We evaluated highly correlated features (e.g., $| {\mathrm{\rho }} | \,\gt\, \{ {0.2,\ 0.25,\ {\mathrm{and\ 0}}{\mathrm{.3}}} \}$) as well as positively correlated features (e.g.,${\mathrm{\rho }} \,\gt\, \{ {0.2,\ 0.25,\ {\mathrm{and\ 0}}{\mathrm{.3}}} \}$).

Network features selection: To generate network-based features from the blood modules, eigengenes (the first PC) of each module were calculated. To further explore the effectiveness of network features, we also explored using the top 2, 5, and 10 PCs and the PCs that explained 70, 75, 80, and 85% of the total variance of each module.

### Training and prediction

To develop gene-specific prediction models, we applied 5-fold cross-validation (CV), where in each fold, 4/5 of the data were used for training and 1/5 served as a hold-out test set. In each training subset, we select features by choosing (i) top PCs from PCA on blood transcriptome data, (ii) highly correlated blood gene markers with target brain genes, (iii) top PCs from each network module, and (iv) union of highly correlated blood gene markers and top PCs of blood gene expression network modules. Then, we performed both linear regression, using the lm() function, and elastic net modeling, using the glmnet() function, on the selected features in R (version 4.2.0). In addition to these linear approaches, we evaluated several nonlinear machine learning methods within a unified training framework implemented via the caret package. Specifically, we trained random forest (method = “rf”), support vector machines (method = “svmLinear2”), and gradient boosting models (method = “xgbTree”), with model tuning and evaluation conducted using CV and optimization of the *R*-squared metric. To avoid overfitting, linear regression models were fitted only when the number of selected features was <1,000. For each gene, we have performed 70 different feature selection methods using various cutoffs and approaches as explained above. The performance of each model was evaluated by calculated average CV correlation coefficient, *r*, between predicted and observed expression values in the corresponding hold-out (e.g., test) data from each fold.

### Prediction models

We trained models to predict expression of individual brain genes from blood gene expression. The dependent variable *y* is the TMM-normalized, log_2_-transformed expression of a single brain gene, and residualized for sex and sequencing covariates (SMCENTER, SMRIN, SMTSISCH, SMEXNCRT, SMRRNART, SMNTERRT). The features *X* are normalized expression levels of all blood genes.

Linear regression models were defined as


\begin{eqnarray*}
{{y}_{{\mathrm{ brain}}}} = {{\beta }_0} + \mathop \sum \limits_{i = 1}^p {{\beta }_i}{{x}_{{\mathrm{ blood}},i}} + \in,
\end{eqnarray*}

where ${{\beta }_i}$ are regression coefficient, *p* is the number of blood features, and $\epsilon $ is residual error.

Elastic net models extended this formulation with L1/L2 regularization to improve prediction stability for high correlated features:


\begin{eqnarray*}
\hat{\beta } &=& {\mathrm{arg\ }}\mathop {\min }\limits_\beta \left\lbrace \frac{1}{{2n}}\mathop \sum \limits_{j = 1}^n {{{\left( {{{y}_j} - {{\beta }_0} - \mathop \sum \limits_{i = 1}^p {{\beta }_i}{{x}_{ji}}} \right)}}^2}\right. \\&&\left.+\, \lambda \left( {\alpha \mathop \sum \limits_{i = 1}^p \left| {{{\beta }_i}} \right| + \frac{{1 - \alpha }}{2}\mathop \sum \limits_{i = 1}^p \beta _i^2} \right) \right\rbrace,
\end{eqnarray*}

where $\lambda $ controls regularization strength and $\alpha \in [ {0,1} ]$ balances L1 (lasso) and L2 (ridge) penalties. Optimal $\alpha $ and $\lambda $ were selected using 10-fold CV, testing $\alpha $ values from 0 to 1 in increments of 0.1. If fitting failed, the model was refit as ridge regression ($\alpha = 0$).

### Pathway enrichment

For functional analysis of the genes, we used the R package enrichR with the GO-Biological Process and Reactome databases [19–21].

### ROSMAP data

Gene expression data from the dorsolateral prefrontal cortex in the ROSMAP cohort were obtained from RNA-seq and corrected for postmortem interval, RNA integrity number (RIN), sex, study, and batch effects. Monocyte gene expression data were corrected for exonic rate, sex, study, and batch effects, with 279 individuals having paired monocyte and brain expression data [22].

## Results

### Distinct information captured by supervised and unsupervised feature selection

To identify the most effective feature selection mechanism for brain gene expression, we utilized 4 different approaches within the training sets from 5-fold CV. We first applied PCA on the blood transcriptome data and used the top PCs as features for predicting gene expression in the brain tissues (i.e., unsupervised feature selection). The top 40, 80, 100, and 120 PCs as well as the PCs that explain 80, 85, 90, and 95% of the variance (denoted as gPC(.)) were selected as features.

As an alternative approach, we also identified blood genes whose gene expression profiles were correlated with a target brain gene (i.e., supervised feature selection) by Pearson’s correlation coefficient thresholds of |*r*| > 0.2, 0.25, and 0.3, as well as *r* > 0.2, 0.25, and 0.3 (denoted as RG(.)). Thirdly, we extracted the module features by selecting the top 1, 2, 5, and 10 PCs, and the PCs that explained 70, 75, 80, and 85% of the variance of each co-expressed gene module (denoted as mPC(.)). Lastly, we combined the module features with blood genes that were highly correlated with a given brain gene.

Prediction accuracy was assessed as the average correlation between the predicted brain gene expression values and the actual brain gene expression values across 5 folds. The number of predicted genes (with an average CV correlation coefficient, *r* > 0.1) varied by brain region and feature selection method. When using the top-performing method for each region, the number of predicted genes ranged from 10,583 in hippocampus to 15,253 in the cerebellum (PAXgene-preserved), with corresponding method-specific configurations noted in Supplementary Table S2. The number of genes predicted with moderate accuracy (*r* ≥ 0.6) ranged from 65 to 134, with the highest count observed in the cerebellum using a combination of correlation-based and module-derived features. High-confidence predictions (*r* ≥ 0.9) were achieved for 2 –10 genes per region, again most frequently in the cerebellar hemisphere.

To more systematically evaluate prediction performance across accuracy thresholds, we quantified the number of predicted genes at 9 *r* thresholds (0.1 through 0.9) across brain regions (Supplementary Fig. S1). When using only the top-performing feature selection method, the cerebellum (PAXgene-preserved) and cortex (PAXgene-preserved) showed the highest number of predicted genes with *r* > 0.1 (15,253 and 15,136, respectively), while the putamen and hippocampus showed the lowest (11,323 and 10,583, respectively). At *r* > 0.5, prediction counts ranged from 363 in the cerebellum to 130 in the anterior cingulate cortex. At the highest accuracy threshold (*r* > 0.9), gene counts ranged from 10 in the cerebellar hemisphere to 2 in the anterior cingulate. When aggregating predictions from the top 5 feature selection methods, these numbers increased overall, with the substantia nigra and hypothalamus showing the most genes at *r* > 0.1 (18,335 and 18,100, respectively), and the hippocampus showing the least (16,373). For *r* > 0.5, the cerebellum again had the highest count (653), and the putamen the lowest (202). At *r* > 0.9, the cerebellar hemisphere had the most genes (13), and the anterior cingulate cortex the fewest (2). One contributing factor to the variation in predictive performance across brain regions may be the number of available paired samples, which is highest for the cortex, cerebellum, caudate, and cerebellar hemisphere (Fig. 2A), potentially enhancing model robustness and prediction accuracy in these regions.

Overall, unsupervised PC-based feature selection predicted a larger number of genes, but with lower accuracy (average CV *r* between 0.1 and 0.6), whereas correlation-based feature selection predicted fewer genes, but with much higher accuracy (*r* ≥ 0.6). Figure 2B shows these trends in the frontal cortex, and Fig. 2C shows how different feature selection strategies contribute across accuracy thresholds, suggesting that integrating supervised and unsupervised approaches can improve predictive power.

**Figure 3:**
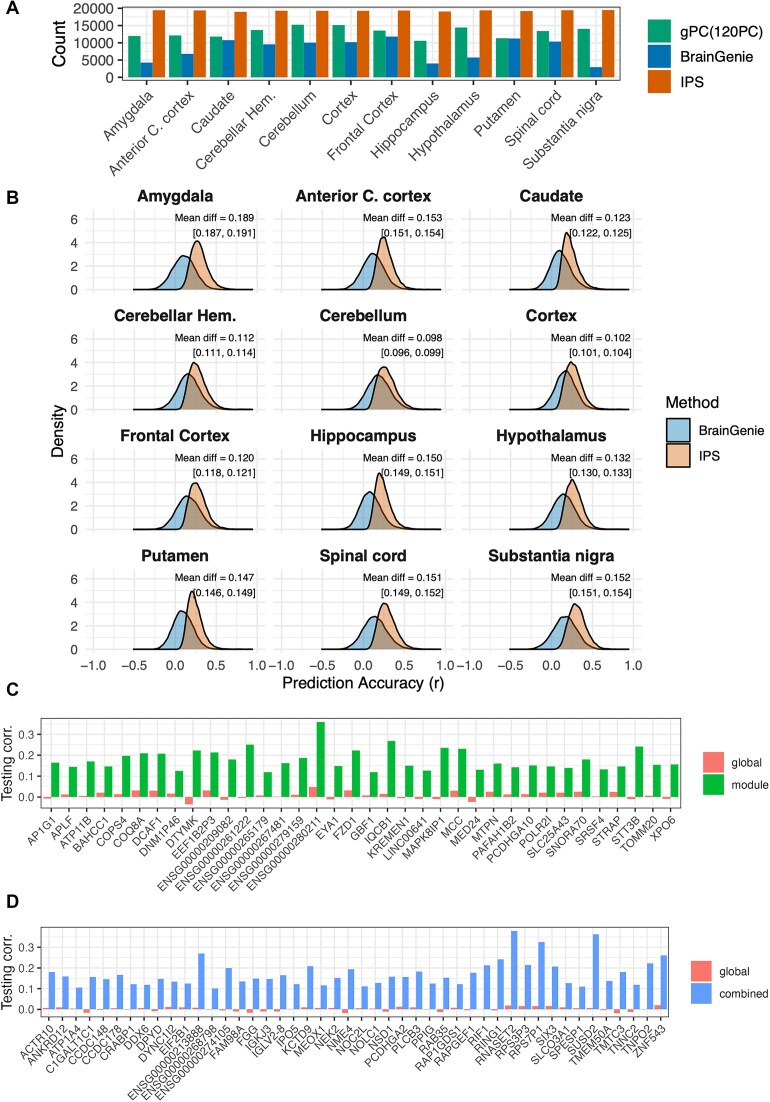
Comparative performance and accuracy gains of feature selection strategies for brain gene expression prediction. (A) Comparison of the performance of the proposed method, IPS, with existing approach. The *x*-axis shows the number of genes predicted with an average CV *r* > 0.1 when using the top 120 PCs from blood transcriptome, BrainGenie (top 40 PCs), and when integrating the results from all 70 feature selection approaches proposed in this study (i.e., IPS). (B) Density distributions of CV *r* across genes for each brain region. Mean differences are reported as mean ± 95% CI. (C) Genes showing the greatest improvement in prediction accuracy (>500%) when using only module features compared to using only global features in the frontal cortex. (D) Genes showing the greatest improvement in prediction accuracy (>1,000%) when using a combination of global and module features vs. global features alone in the frontal cortex.

To ensure a direct comparison with the recently developed BrainGENIE method [15], which uses the top 40 blood PCs within a linear modeling framework for all genes, we trained a linear model using the top 40 PCs and identical training and testing splits as those used for IPS. Under this matched setting, IPS consistently yielded a higher number of predicted genes (avg CV *r* > 0.1) across all brain tissues (Fig. 3A). For example, in the cerebellum 15,247 genes were predicted by using the top 120 PCs (e.g., gPC(120 PC)), whereas BrainGENIE predicted only 10,048 genes. We further compared the distribution of CV correlation coefficients (Fig. 3B), which shows that IPS is consistently shifted toward higher CV *r* values relative to BrainGENIE, demonstrating improved prediction performance across genes.

In addition to this direct comparison, we examined previously published approaches, including TEEBoT [13] and B-GEX [14]. Both methods were trained on earlier GTEx releases with smaller sample sizes, and direct retraining under identical conditions was not feasible. Available results were limited to 2 regions for TEEBoT (caudate and cerebellum) and one region for B-GEX (cerebellum). Across all evaluated accuracy thresholds (*r* > 0.0–0.9), IPS consistently predicted substantially more genes than TEEBoT in both caudate and cerebellum. For example, at a moderate threshold (*r* > 0.3), IPS predicted 4,153 vs. 344 genes in caudate and 7,930 vs. 1,241 in cerebellum, with this advantage persisting even at stringent thresholds (e.g., *r* > 0.8). For cerebellum, B-GEX reported a mean gene-level correlation of 0.044, which was substantially lower than that achieved by our integrated IPS framework (0.286). While these comparisons are constrained by differences in training data versions and available outputs, they further support the improved performance of IPS.

While more complex nonlinear models can theoretically capture higher-order interactions, our comparisons showed no performance advantage over linear or elastic net models. We evaluated these approaches using hippocampus data under a simplified setting that excluded network module features to reduce both model and computational complexity. As shown in Supplementary Table S3, these comparisons did not demonstrate a performance advantage over linear or elastic net models. Given the limited sample size and correlated transcriptomic features, linear models provided a stable solution in this context.

### Blood network features enhance the accuracy of predictions for specific genes

We have constructed a blood tissue co-expression network using MEGENA and determined the top PCs of each co-expression network module as module features (i.e., mPC(.)). We have determined the module eigengene (the first PC) as well as the first 2, 5, and 10 PCs, and the PCs that explained 70, 75, 80, and 85% of the variance of each module. The prediction accuracy for genes, ranging from 2,395 to 3,043, with an average CV correlation *r* > 0.1, was increased by at least 5% across different brain regions when module features were used (Supplementary Table S4). Figure 3C illustrates the genes that showed the most notable improvement in prediction accuracy within the frontal cortex.

We then assessed whether integrating features derived directly from the blood transcriptome (e.g., global features) with those derived from the blood network (e.g., module features) improves predictive accuracy for specific genes. This approach improved the prediction accuracy for a greater number of genes. Specifically, the accuracy of genes, ranging from 6,228 to 8,777, with an average CV *r* > 0.1, was increased by at least 5% across all brain regions (Supplementary Table S5). Figure 3D illustrates the genes that showed the most notable improvement in prediction accuracy within the frontal cortex.

**Figure 4:**
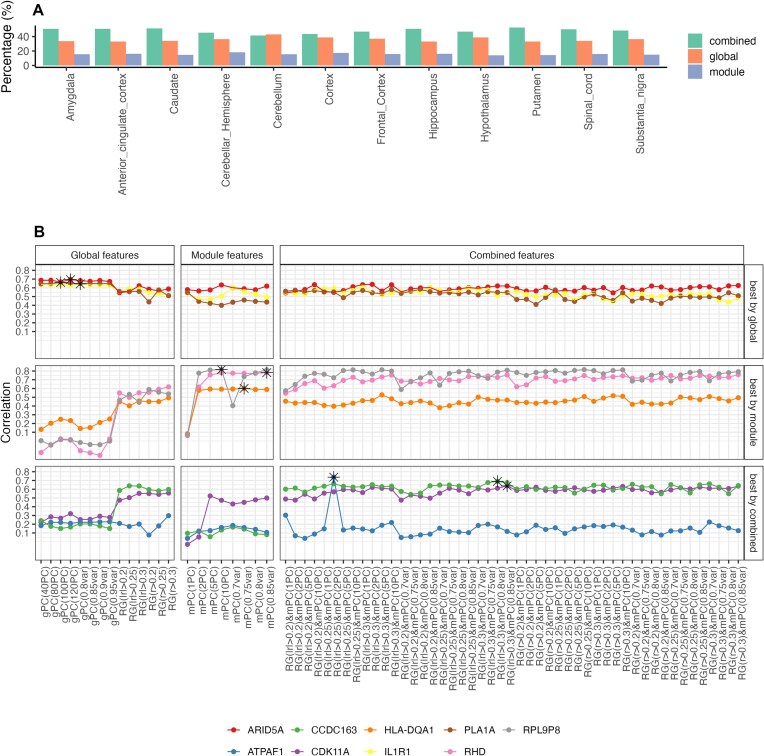
Performance of different feature sets. (A) The percentage of genes for which each feature set performed best across different brain regions. (B) Performance of different feature selection strategies in the frontal cortex, measured by correlation, for various genes.

Overall, the combination of module features and global features had the best performance by predicting half of the profiled genes with *r* >0.1, while global features alone accounted for 35% of the genes, and module features alone contributed to 15% across all the brain regions (Fig. 4A). Figure 4B shows the performance of different gene sets, highlighting the feature sets that yielded the best results for each. Utilizing different feature sets and feature selection approaches captures complementary, orthogonal information, each contributing to the prediction of different genes.

### Gene-specific power of different feature selection methods

To evaluate the performance of all proposed feature selection methods, we identified the top 5 methods with the highest number of predicted genes with an average CV *r* > 0.5. Rather than relying on a single feature selection strategy, we assessed whether combining multiple approaches would improve predictive coverage. Specifically, for each gene, we selected the best-performing method among the top 5 feature selection approaches. This combined strategy increased the number of well-predicted genes (*r* > 0.5) compared to using any single method alone. The effect of combining an increasing number of feature selection methods (from 1 to 5) on the number of predicted genes across brain regions is shown in Supplementary Fig. S1. For 10/12 brain regions, the feature selection method predicting the most genes with *r* > 0.5 was the combination of module and global features (Supplementary Fig. S2). The unsupervised (PCA) method applied on blood transcriptome was the top method for the remaining 2/12 brain regions (spinal cord and substantia nigra) and was the second best for the 9/12 regions. Interestingly, the second-best method was the supervised feature selection for amygdala.

Overall, the ranking of the top 5 methods varied across brain regions, indicating that no single feature selection strategy consistently dominated across tissues. Instead, different approaches captured complementary predictive signals. Supplementary Fig. S2 summarizes these results using a Venn diagram representation of the 5 top-performing feature selection methods, illustrating their overlap and relative ranking across brain regions. In the figure, the method predicting the largest number of genes with average CV *r* > 0.5 is highlighted in red, followed by the second-best in green, the third in purple, the fourth in blue, and the fifth in orange.

Supplementary Table S6 provides a detailed breakdown of the feature combinations and gene prediction performance across brain tissues. For each tissue and correlation threshold (accuracy cutoff; mean CV *r*), the table reports the number of genes predicted above the specified cutoff when combining different numbers of top-performing feature selection methods (1–5 methods). For each combination level, predictions were generated by selecting, for each gene, the best-performing method among the top-ranked methods for that tissue. The table also lists the specific feature selection method(s) included in each combination.

Consistent with the results summarized in Supplementary Figs S1 and S2, these results highlight that different feature selection strategies capture complementary predictive signals. As a result, combining multiple top-performing approaches increases the number of genes that can be accurately predicted (*r* > 0.5) compared to relying on a single method alone.

Notably, each gene-brain region model was trained using paired blood and brain expression data specific to that tissue, which optimizes performance for the corresponding region. Because regulatory relationships and co-expression patterns differ across brain regions, applying models trained in one region to another is not recommended.

### Preservation of age-associated expression patterns in predicted gene profiles

As a representative analysis, we examined whether our predictive models preserve age-associated gene expression patterns in the hippocampus. To evaluate how preservation of age-associated expression patterns depends on prediction accuracy, we stratified genes into 3 groups based on cross-validated prediction performance (CV *r*): poorly predicted (CV *r* < 0.1; *n* = 536), moderately predicted (0.1 ≤ CV *r* ≤ 0.5; *n* = 18,753), and well-predicted (CV *r* > 0.5, *n* = 353). For each gene, we computed the correlation between observed expression and age (*r*₁) and between predicted expression and age (*r*₂).

**Figure 5:**
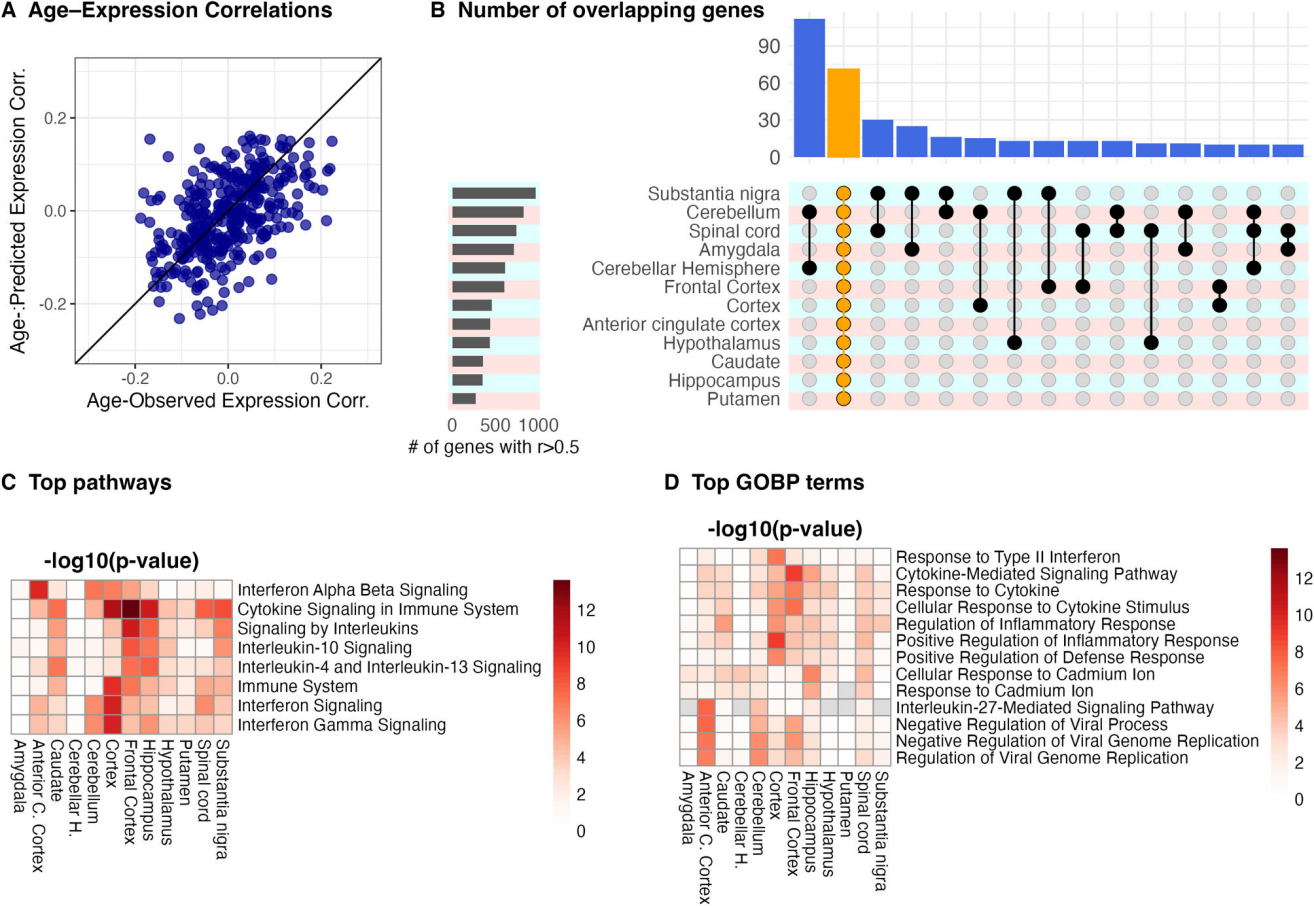
Analysis of accurately predicted brain genes (*r* > 0.5). (A) Correlation between age-association patterns based on observed vs. predicted gene expression for genes with prediction accuracy CV *r* > 0.5 in the hippocampus. Each point represents a gene; the *x*-axis shows correlation with age based on observed expression, and the *y*-axis shows correlation with age based on predicted expression. (B) Number of genes predicted with *r* > 0.5 accuracy across different brain genes. UpSet plot highlights the number of genes that are accurately predicted in multiple brain regions. (C) Top canonical pathways of the accurately predicted genes in each brain region. The color intensity indicates the significance of enrichment (−log10 *P*-value). (D) Top gene ontology biological processes of the accurately predicted genes in each brain region. The color intensity indicates the significance of enrichment (−log10 *P*-value).

Correspondence between observed and predicted age associations increased with prediction accuracy: poorly predicted genes had *r* = 0.195, moderately predicted genes *r* = 0.296, and well-predicted genes *r* = 0.496 (Supplementary Table S7). Similarly, the mean absolute difference between r₁ and r₂ decreased from 0.10 in poorly predicted genes to 0.06 in well-predicted genes. These results demonstrate that predicted expression preserves age-associated patterns primarily for the genes with higher prediction accuracy, while preservation is weaker for poorly predicted genes. The relationship between *r*₁ and *r*₂ is visualized in Fig. 5A, highlighting the consistency between observed and predicted age associations for well-predicted (CV *r* > 0.5) genes. This analysis supports the interpretation that age-related variation is partially retained in predicted expression and highlights the dependency on prediction quality. While this analysis was conducted in the hippocampus, the same approach can be extended to other brain regions as well as to other phenotypes beyond age, such as disease status.

### Immune-related genes exhibit greater predictive power

To assess biological functions of the accurately predicted genes (*r* > 0.5) in each brain region, we performed pathway enrichment analysis using EnrichR [19]. On average, 565 genes were predicted with *r* > 0.5 across all brain regions, with the number of predicted genes ranging from 273 in the putamen to 976 in the substantia nigra (Fig. 5B). We observed significant enrichment in immune-related pathways, including cytokine-mediated signaling, interleukin signaling, interferon signaling, and response to cytokine, across most brain regions (Fig. 5C–D). The enrichment was strongest in the cortex (right cerebral frontal pole cortex, sampled at donor collection site and preserved in PAXgene fixative) and frontal cortex (right cerebral frontal pole cortex, sampled at the Miami Brain Bank and preserved as fresh frozen tissue). One potential explanation for this observation is the interaction between the brain and peripheral immune systems, which share common pathways and mechanisms, thereby leading to correlated changes in gene expression. Additionally, it is known that systemic immune cells, which can mobilize and directly infiltrate the brain parenchyma—influence gene expression in brain-resident immune cell responses.

### Top blood-based predictors

**Figure 6:**
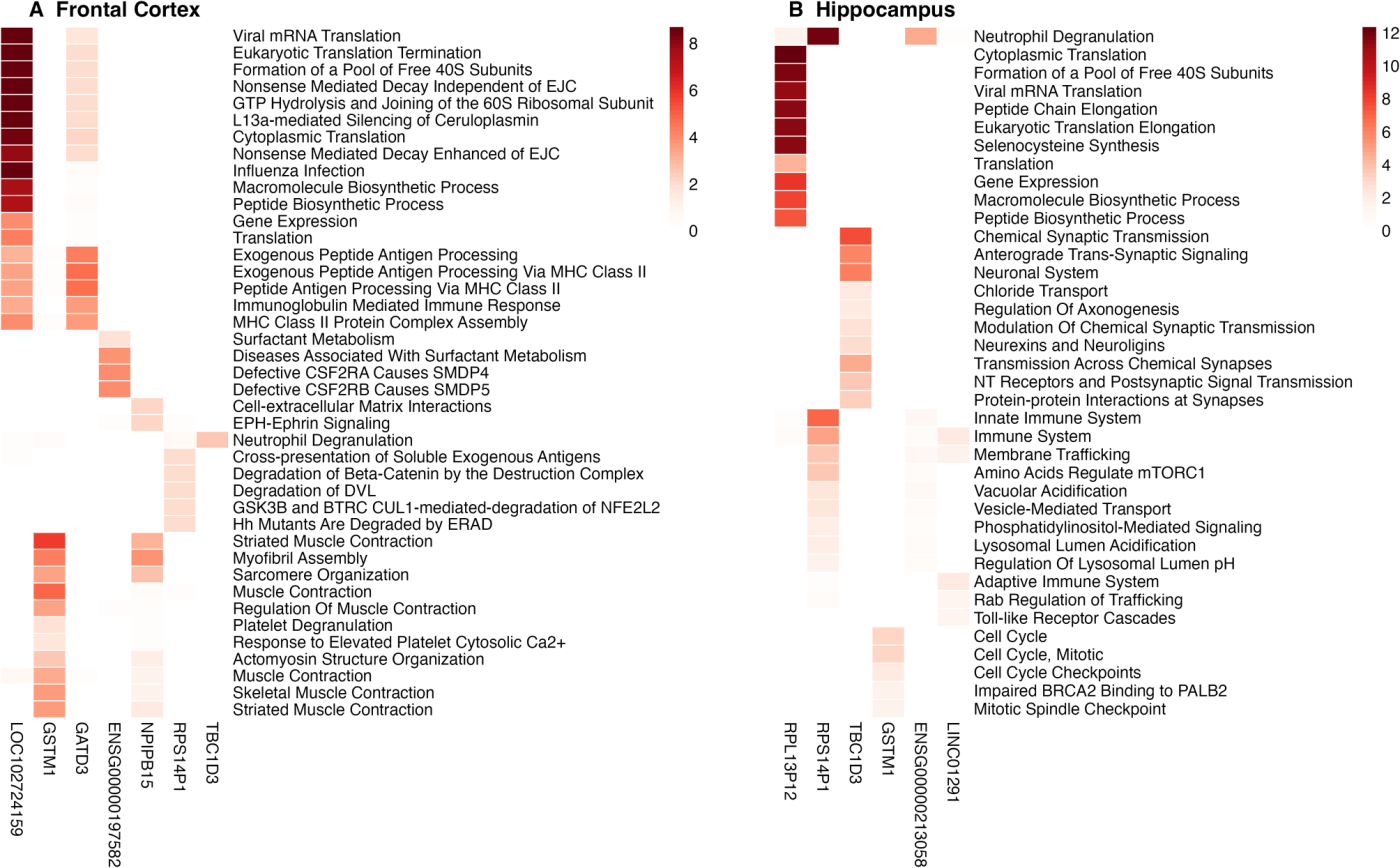
Analysis of the top blood-based predictors for brain genes selected based on the top 10 highest prediction accuracies. Pathway enrichment analysis of the top blood biomarkers for genes (A) in the frontal cortex and (B) in the hippocampus. Blood-based predictors were selected based on their correlation with target brain genes (|*r*| > 0.2) selection in at least 2 out of 5 CV folds. The color intensity represents the significance of the enrichment (−log10 *P-*value).

To better understand the interactions between the blood biomarkers, and the brain genes, we assessed the most informative features of the brain genes with the highest prediction accuracy. Initially, the top 10 brain genes exhibiting the highest accuracy in the frontal cortex were identified, including *NPIPB15, GSTM1, ENSG00000213058, RPS14P1, ENSG00000197582, GATD3, RPL13P12, TBC1D3, LINC01291, LOC102724159*, with average CV correlation coefficients (CV *r*) ranging from 0.89 to 0.94. Similarly, the top 10 genes with the highest accuracy in the hippocampus were determined, namely *GSTM1, NPIPB15, GATD3, ENSG00000213058, RPS14P1, LINC01291, TBC1D3, ENSG00000262539, RPL13P12*, and *LOC102724023*, with average CV *r* values between 0.86 and 0.95. Subsequently, for each gene, the top blood biomarkers were identified based on their correlation with the target brain gene (|*r*| > 0.2) within each CV fold. The top blood-based predictors were defined as those selected in at least 2 out of 5 CV folds. Pathway enrichment analysis was then performed on these top features (Fig. 6). The results showed that the blood predictors for the genes *GATD3* and *LOC102724159* were significantly enriched in immune response-related pathways, including immunoglobulin-mediated immune response and antigen processing, which might suggest a role in immune response. The blood predictors for *GSTM1* and *NPIPB15* were enriched in pathways associated with muscle contraction. Additionally, the blood predictors of the gene *TBC1D3*, a gene promoting dendritic arborization and protracting the pace of synaptogenesis [23], were enriched in the synaptic signaling pathways, while those for *RPL13P12* were enriched in biosynthetic process. Finally, the blood predictors for *RPS14P1* were enriched in pathways related to neutrophil degranulation and innate immune system. These results highlight the complex interactions between peripheral biomarkers and brain functions.

### Prediction performance is not driven by gene expression abundance

To assess whether prediction performance was influenced by gene expression abundance, we examined the relationship between gene expression level and prediction accuracy across brain regions. For each region, we computed the correlation between gene expression abundance (mean expression level across samples) and cross-validation performance (CV *r*), separately using gene expression levels measured in blood and in the corresponding brain tissue (Supplementary Figs S3 and S4). Across all regions, the correlation between expression abundance and prediction accuracy was negligible (|*r*| < 0.1), indicating that predictive performance is not systematically driven by highly expressed genes. Notably, top-performing genes exhibited moderate expression levels in both blood and brain tissues.

### Application of the prediction pipeline to the ROSMAP cohort

To assess the behavior of our framework in an independent dataset with a different experimental context, we applied the same pipeline to the ROSMAP cohort using monocyte RNA-seq data as predictors [22]. Brain expression in ROSMAP was derived from the dorsolateral prefrontal cortex, while in GTEx, we used frontal cortex (BA9, snap frozen) samples. These regions are anatomically similar but not perfectly matched. Importantly, this analysis does not constitute an external validation, as the predictor modality (monocytes vs. whole blood), cohort characteristics (AD and controls vs. healthy individuals), and potential differences in tissue sampling and processing remain.

Using identical feature selection procedures and 5-fold CV, we evaluated prediction performance across correlation thresholds (Supplementary Fig. S5-A). Across all thresholds, the number of predictable genes was constantly higher in GTEx than in ROSMAP. These differences likely reflect a combination of factors, including the broader cellular composition captured in whole blood relative to monocytes. The results highlight the sensitivity of prediction performance to both biological context and study design, while demonstrating that the proposed pipeline can be flexibly applied across datasets.

To further evaluate the consistency of gene-level predictions across datasets, we examined the overlap between the top-ranked predicted genes in GTEx and ROSMAP. Across multiple cutoffs (top 10 to top 2,000 genes), the number of overlapping genes exceeded expectations under a null model, with statistically significant enrichment observed at all thresholds (Supplementary Fig. S5-B). For example, 13 genes overlapped among the top 50 (*P* = 1.19 × 10^−24^), and 236 genes overlapped among the top 2000 (*P* = 1.26 × 10^−7^). This enrichment indicates that, despite differences in datasets, a subset of genes is consistently well-predicted across both datasets.

### Prediction performance of the AD related genes

To assess the predictive capacity of our models for brain expression of AD-related genes, we evaluated the prediction accuracy of the top 1,000 AD key drivers previously identified in postmortem parahippocampal gyrus samples from the Mount Sinai Brain Bank (MSBB) AD cohort [24]. The analysis was intended to assess the baseline predictability of AD-related genes, rather than to evaluate disease-specific expression changes or external cohort generalizability.

**Figure 7:**
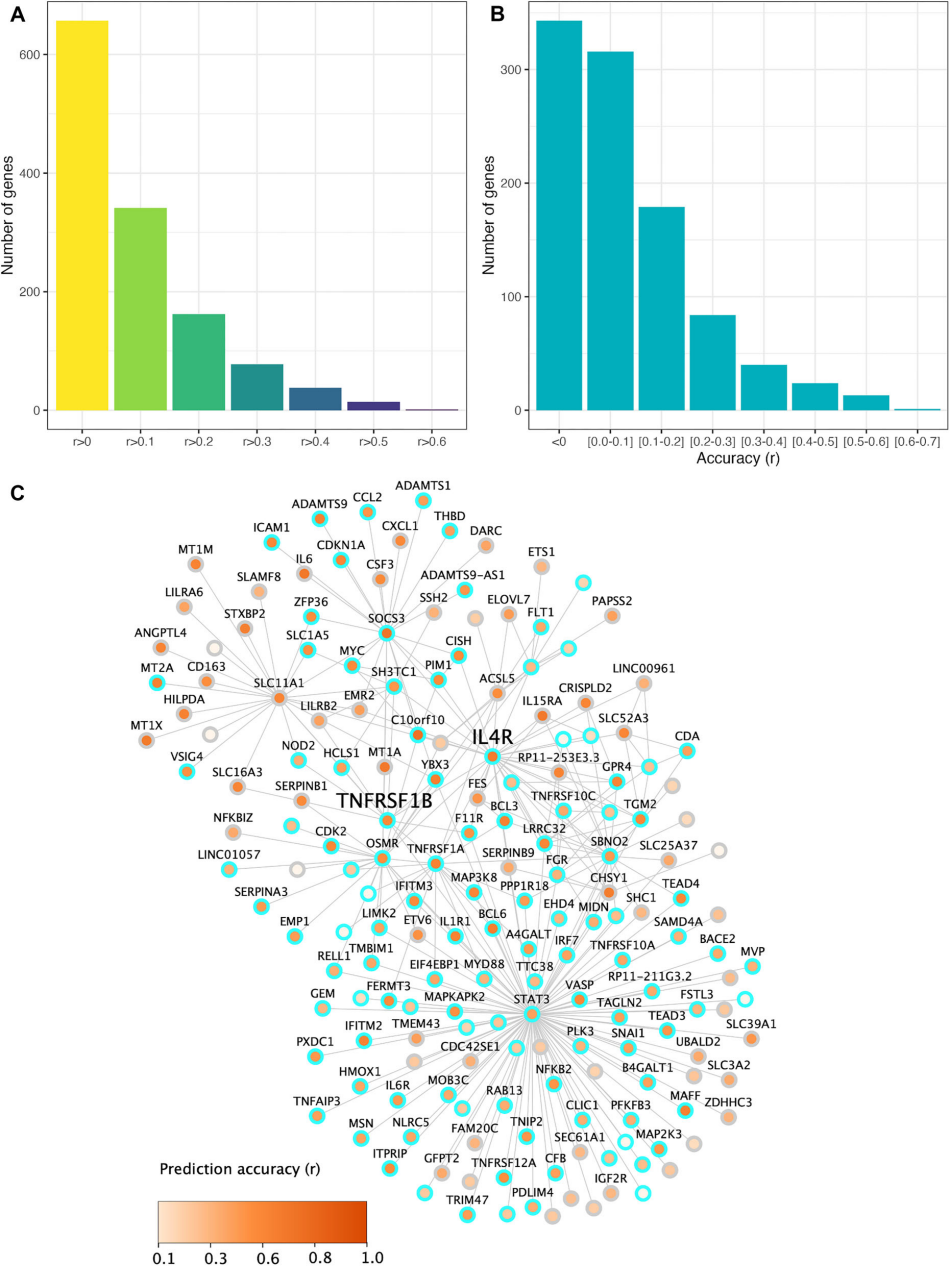
Prediction performance and functional relevance of AD key driver genes in the hippocampus. (A) Cumulative number of top 1,000 AD key driver genes achieving varying levels of prediction accuracy in the hippocampus, measured as the average cross-validated correlation (*r*) between observed and predicted expression. (B) Distribution of gene counts across defined prediction accuracy thresholds, highlighting how many genes fall within each performance range. (C) Co-expression network of IL4R in the hippocampus. Node color reflects prediction accuracy (darker indicates higher accuracy), and nodes with cyan borders are associated with AD based on Clinical Dementia Rating (CDR).

Among those genes, 341 genes exhibited an average CV *r* > 0.1, with 14 genes reaching *r* > 0.5 in the hippocampus (Fig. 7A–B). The IL-4 receptor (*IL4R*) gene demonstrated the highest prediction accuracy with average CV *r* of 0.61. *IL4R* is expressed on microglia and plays a crucial role in regulating microglial phenotype [25, 26]. It has been proposed that IL-4, a ligand for IL4R, may have a protective role in AD by regulating neuroinflammation and amyloid-beta pathology [27].

Another well-predicted AD-related gene is the *TNFRSF1B* gene (avg CV *r* = 0.51), which encodes a protein that is a member of the TNF-receptor superfamily. Genetic variants in *TNFRSF1B* have been associated with cognitive resilience in AD [28]. To further explore the functional context of these genes, we generated hierarchical co-expression networks centered on *IL4R* and *TNFRSF1B* in the hippocampus using the MSBB dataset. These networks were constructed at 2 levels (layer 1 and layer 2 from *IL4R* or *TNFRSF1B*), reflecting immediate and extended co-expression relationships. We assessed the functional relevance of genes within each network layer by evaluating their predictive performance and association with AD pathology. Specifically, we examined the previously established AD associations of these network genes as identified through prior analyses of differential expression patterns across clinical traits, including Clinical Dementia Rating (CDR), Braak & Braak score (bbscore), CERAD score, and plaque density, in AD patients vs. controls from the MSBB dataset [24]. To illustrate the model’s performance, we highlighted a representative layer 2 network associated with CDR for *IL4R*, which is also closely connected to *TNFRSF1B*, in Fig. 7C. These examples demonstrate that the genes that overlap with AD molecular signatures have good prediction performance (172/182 network genes were predicted with average CV *r* > 0.1). Comprehensive results for all network layers and traits are provided in the Supplementary Figures. Full panels of predictive performance plots for *IL4R* (8 conditions: 2 layers × 4 traits) are shown in Supplementary Fig. S6, and corresponding analyses for *TNFRSF1B* are included in Supplementary Fig. S7. Additionally, to further validate the predictive accuracy of our model at the individual gene level, Supplementary Fig. S8 presents observed vs. predicted gene expression values in the hippocampus for the 4 most predictable genes from the 1,000 AD key drivers list: *IL4R, CSDA, MAFF*, and BCL6. These plots highlight the model’s ability to accurately capture gene-specific expression patterns relevant to AD biology.

## Discussion

Understanding the molecular processes in the brain is crucial for improving our knowledge of brain disorders, as gene expression in the brain plays an important role in the pathogenesis of both neurological (i.e., neurodegenerative) and psychiatric diseases. However, direct investigation of brain tissue remains limited due to the invasive nature of sample collection. Consequently, there is a pressing need for non-invasive biomarkers that can serve as reliable proxies for brain gene expression.

In this study, we explored the potential of blood-derived transcriptomic signatures to predict brain gene expression. Blood samples offer a minimally invasive and widely accessible source, making them an attractive alternative for large-scale studies and longitudinal monitoring. By integrating blood transcriptome data with a variety of feature selection techniques, we developed gene-specific predictive models. This gene-specific approach enables the identification of blood biomarkers that are closely tied to specific molecular processes in the brain, offering improved interpretability and potential clinical utility.

The proposed framework is intentionally model-agnostic and flexible, allowing for the integration of diverse machine learning approaches depending on the specific research context. Although linear models performed well in our analyses, particularly at higher correlation thresholds, we do not advocate for a single modeling strategy. Rather, the central contribution of this study is the demonstration that gene-specific feature selection and model selection can substantially improve predictive accuracy compared to conventional genome-wide, one-size-fits-all approaches. This flexibility enables the pipeline to accommodate alternative models, which may further enhance performance for specific genes or datasets.

Despite promising results, several challenges remain. One key challenge is the heterogeneity of whole blood. The complex interactions between different cell types could dilute or conceal the signals that are informative of brain gene activity. Incorporating cell-type deconvolution methods may help improve the signal-to-noise ratio and enhance model performance.

Another major limitation is the lack of independent cohorts with matched brain and blood expression data, which prevents direct external validation of the proposed models. The availability of such matched samples would enable more robust validation of the predictive models and offer a clearer understanding of cross-tissue gene expression relationships. While we leveraged large-scale, publicly available datasets for model training and evaluation, future efforts should prioritize collecting matched multi-tissue datasets, particularly in specific disease contexts, to support more rigorous benchmarking. More broadly, these results highlight the importance of accounting for dataset shift and avoiding over-reliance on performance within a single dataset, as CV alone does not guarantee generalizability to independent or clinical cohorts [29].

Despite this limitation, our findings demonstrate that blood-based transcriptomic models can provide a research tool for inferring brain gene expression, highlighting their potential utility for studying neurological disorders. While these models show promise, their performance in independent or clinical cohorts remains to be established, and external validation in appropriately matched blood and brain samples will be necessary before they can be considered for diagnostic or prognostic applications. Future studies may explore their use in identifying early molecular signatures of disorders such as AD, PD, and major depressive disorder, or for non-invasive monitoring of disease progression, but such applications remain speculative at this stage.

Additionally, although immune-related genes emerged as strong predictors of brain gene expression, the directionality and causality of these associations remain to be clarified. It is plausible that peripheral immune activity reflects or influences neuroinflammatory processes, which are increasingly recognized as key contributors to NDD. Indeed, controlled studies in rodents have now shown the dynamic interactions between peripheral immune compartments and the brain, which support brain–body directionality and causality [30]. Further investigation into the shared regulatory mechanisms between the immune system and the brain will be essential to untangle these relationships.

Our findings underscore the feasibility of using blood-based transcriptomic models to infer brain gene expression and lay the groundwork for future diagnostic and prognostic tools. If validated in clinical cohorts, these models could be employed to identify early molecular signatures of neurological disorders such as AD, PD, and major depressive disorders. Moreover, they may enable non-invasive monitoring of disease progression or response to therapy, offering valuable insights for precision medicine.

Looking ahead, integrating additional layers of omics data—such as epigenetic modifications, proteomics, and metabolomics—may further refine prediction accuracy and enhance biological relevance. Incorporating longitudinal blood samples could also reveal dynamic transcriptomic changes associated with disease progression or treatment. Ultimately, expanding our understanding of peripheral-brain tissue interactions has the potential to bridge a gap in neurodegenerative research and improve clinical outcomes through earlier and more precise intervention.

## Availability of source code and requirements

Project name: Blood Biomarkers of Brain Gene Expression.

Project home page: https://gitlab.com/csbayrak/ips.

Operating system(s): Platform independent.

Programming language: R.

Other requirements: R 4.2 or higher.

License: Data files for examples are distributed under the CC0 1.0 Universal (CC0 1.0) Public Domain Dedication, all code is distributed under the MIT license.

RRID:SCR_027608

## Supplementary Material

giag058_Supplemental_Files

giag058_Authors_Response_To_Reviewer_Comments_original_submission

giag058_GIGA-D-25-00434_original_submission

giag058_GIGA-D-25-00434_revision_1

giag058_Reviewer_1_Report_original_submission Reviewer 1 -- 12/9/2025

giag058_Reviewer_1_Report_revision_1 Reviewer 1 -- 4/7/2026

giag058_Reviewer_2_Report_original_submission Reviewer 2 -- 1/11/2026

giag058_Reviewer_2_Report_revision_1 Reviewer 2 -- 5/5/2026

## Data Availability

The gene expression data and the associated sample information are publicly available through the GTEx Consortium portal. Specifically, we used GTEx Analysis V8 bulk tissue RNA-seq gene read count data and the corresponding meta files [16]. The code and input files for an illustrative example are available on GitLab (https://gitlab.com/csbayrak/ips), and a snapshot of the project has been archived on Zenodo under doi:10.5281/zenodo.17477449 [31]. The IPS workflow is registered in WorkflowHub doi:10.48546/workflowhub.workflow.2139.1 [32]. ROSMAP gene expression data were obtained from the Religious Orders Study and Memory and Aging Project cohort and are available through the AMP-AD Knowledge Portal, under controlled access (Synapse accession: syn2580853). DOME-ML annotations can be accessed via the DOME registry (accession xrf3z08hoe) [33].

## Additional files

**Supplementary Table S1:** Demographic characteristics of individuals with paired whole blood and brain tissue samples included in the analysis.

**Supplementary Table S2:** Number of predicted genes at various accuracy cutoffs.

**Supplementary Table S3:** Number of genes predicted in the hippocampus by different machine learning models across varying accuracy thresholds. Feature selection included principal component analysis (PCA) and correlation-based filtering, excluding network-based features.

**Supplementary Table S4:** Number of genes with greater prediction accuracy (>5%) when using only module features compared to global features.

**Supplementary Table S5:** Number of genes with greater prediction accuracy (>5%) when using combined features compared to global features.

**Supplementary Table S6:** Number of genes predicted above correlation thresholds using combinations of 1–5 methods per brain region.

**Supplementary Table S7:** Analysis of age-associated expression patterns. Genes were binned by prediction accuracy (CV *r*), and the correlation between observed (*r*₁) and predicted (*r*₂) age associations was computed for each bin.

**Supplementary Figure S1:** Prediction performance across brain regions and feature selection combinations. Each panel displays the number of predicted genes (*y*-axis) at increasing prediction accuracy thresholds (*x*-axis, cross-validation correlation coefficient *r*) for a given number of combined feature selection methods (from 1 to 5). Each line represents a different brain region.

**Supplementary Figure S2:** Top 5 feature selection methods based on the number of genes predicted with average CV *r* > 0.5. The method that predicted the most genes is annotated in red, the second in green, the third in purple, the fourth in blue, and the fifth in orange.

**Supplementary Figure S3:** Relationship between blood gene expression abundance and prediction accuracy across brain regions. Scatter plots show the association between gene expression abundance measured in blood (*x*-axis; mean expression across samples) and prediction accuracy (*y*-axis; cross-validation correlation coefficient, CV *r*) for each brain region. Each point represents a gene. A linear regression line with 95% confidence interval is shown in red. The Pearson correlation coefficient between gene abundance and prediction accuracy is displayed in each panel.

**Supplementary Figure S4:** Relationship between brain gene expression abundance and prediction accuracy across brain regions. Scatter plots show the association between gene expression abundance measured in the corresponding brain tissue (*x*-axis; mean expression across samples) and prediction accuracy (*y*-axis; cross-validation correlation coefficient, CV *r*) for each brain region. Each point represents a gene. A linear regression line with 95% confidence interval is shown in red. The Pearson correlation coefficient between gene abundance and prediction accuracy is displayed in each panel.

**Supplementary Figure S5:** Comparison of gene prediction performance between GTEx and ROSMAP. (A) Number of genes predicted across correlation thresholds (*r* > 0.1 to *r* > 0.9). (B) Overlap of top-ranked genes across datasets at increasing cutoffs; points are colored by −log_10_(*P*), and the red dashed line indicates the expected overlap under the null. Observed overlaps exceed expectation at all thresholds.

**Supplementary Figure S6:** Co-expression networks of IL4R in the hippocampus across AD-related traits.

**Supplementary Figure S7:** Co-expression networks of TNFRSF1B in the hippocampus across AD-related traits.

**Supplementary Figure S8:** Observed versus predicted gene expression in the hippocampus for the 4 most predictable genes among the 1,000 AD key drivers: IL4R, CSDA, MAFF, and BCL6.
