# Supplementary material for: Leveraging machine learning and network biology approaches to predict brain gene expression from blood transcriptomes
Source: Gigascience. 2026 May 18;15:giag058. doi: 10.1093/gigascience/giag058 (PMC13201078; doi:10.1093/gigascience/giag058)
Supplement: giag058_Supplemental_Files [file giag058_supplemental_files.zip › SupplFigs_May2026.pdf]

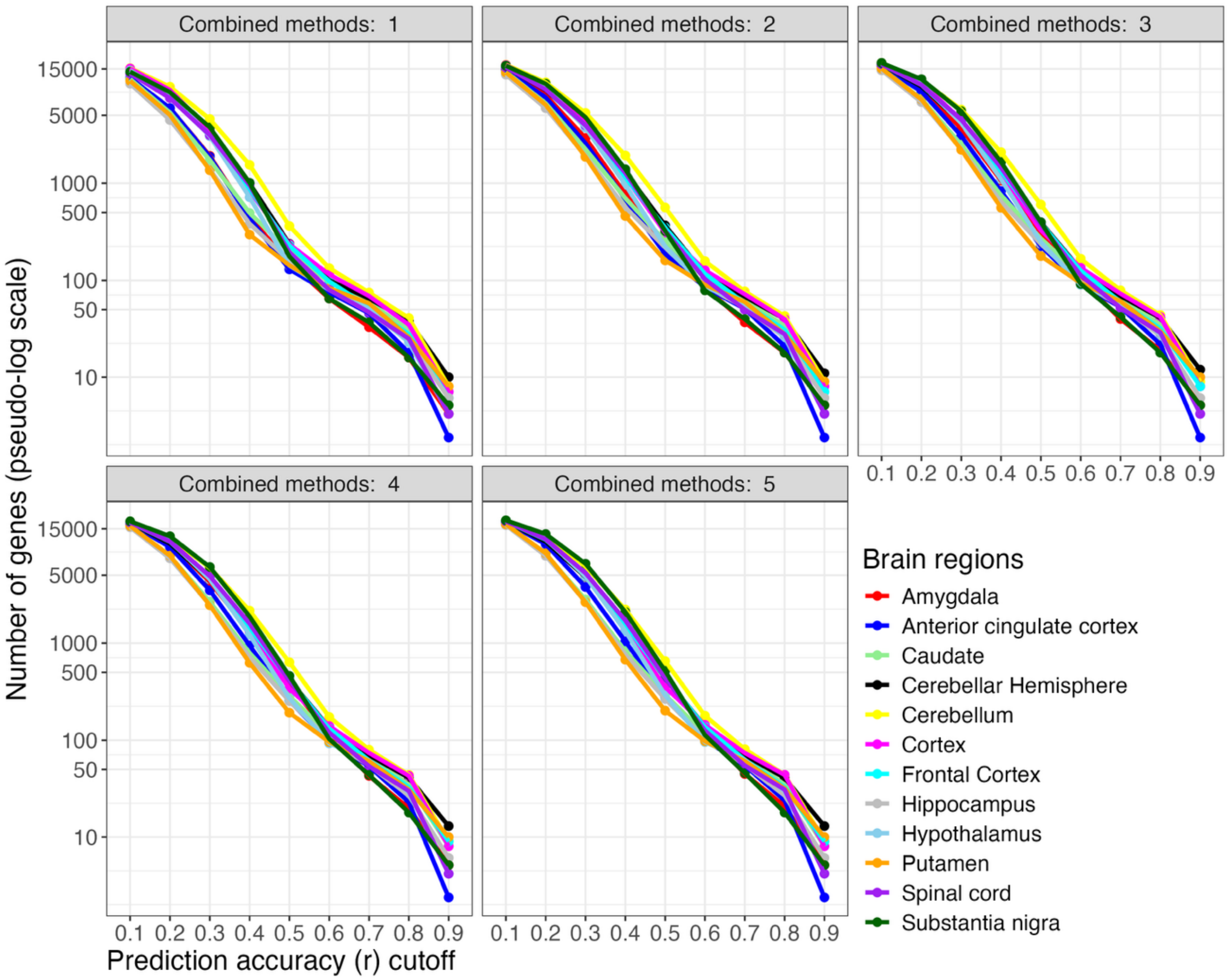

Figure S1. Prediction performance across brain regions and feature selection combinations. Each panel displays the number of predicted genes (y-axis) at increasing prediction accuracy thresholds (x-axis, cross-validation correlation coefficient r) for a given number of combined feature selection methods (from 1 to 5). Each line represents a different brain region.

Amygdala

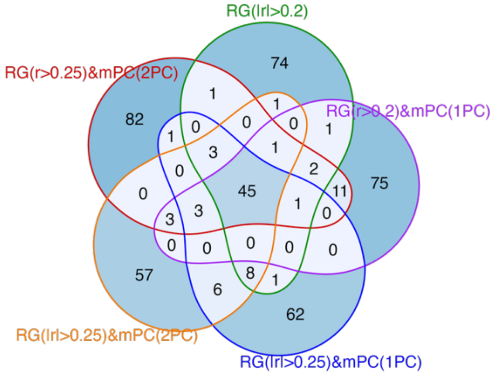

Anterior Cingulate Cortex

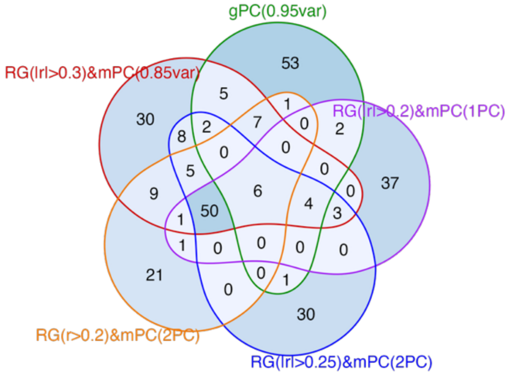

Caudate

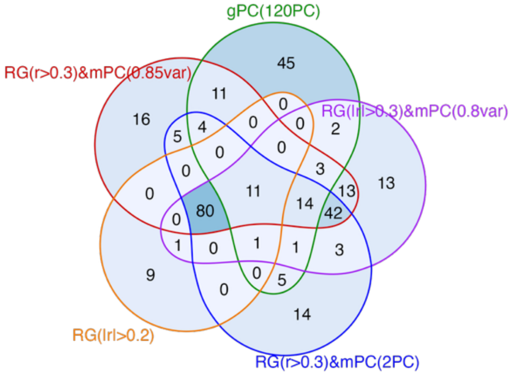

Cerebellar Hemisphere

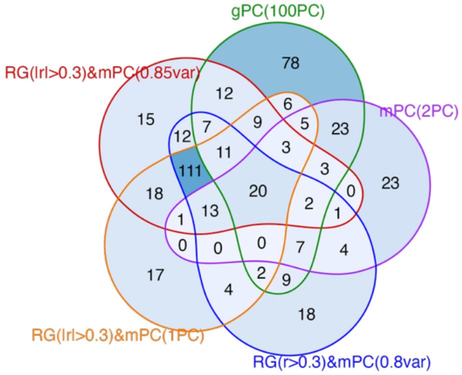

Cerebellum

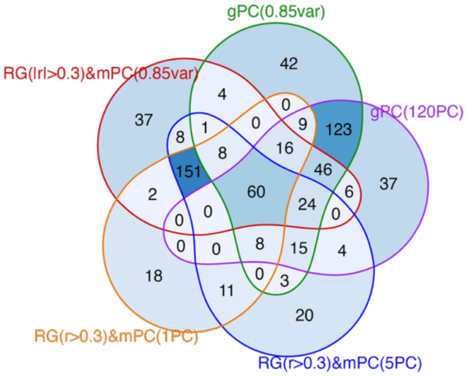

Cortex

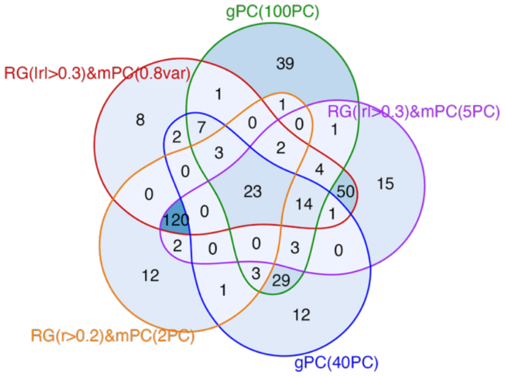

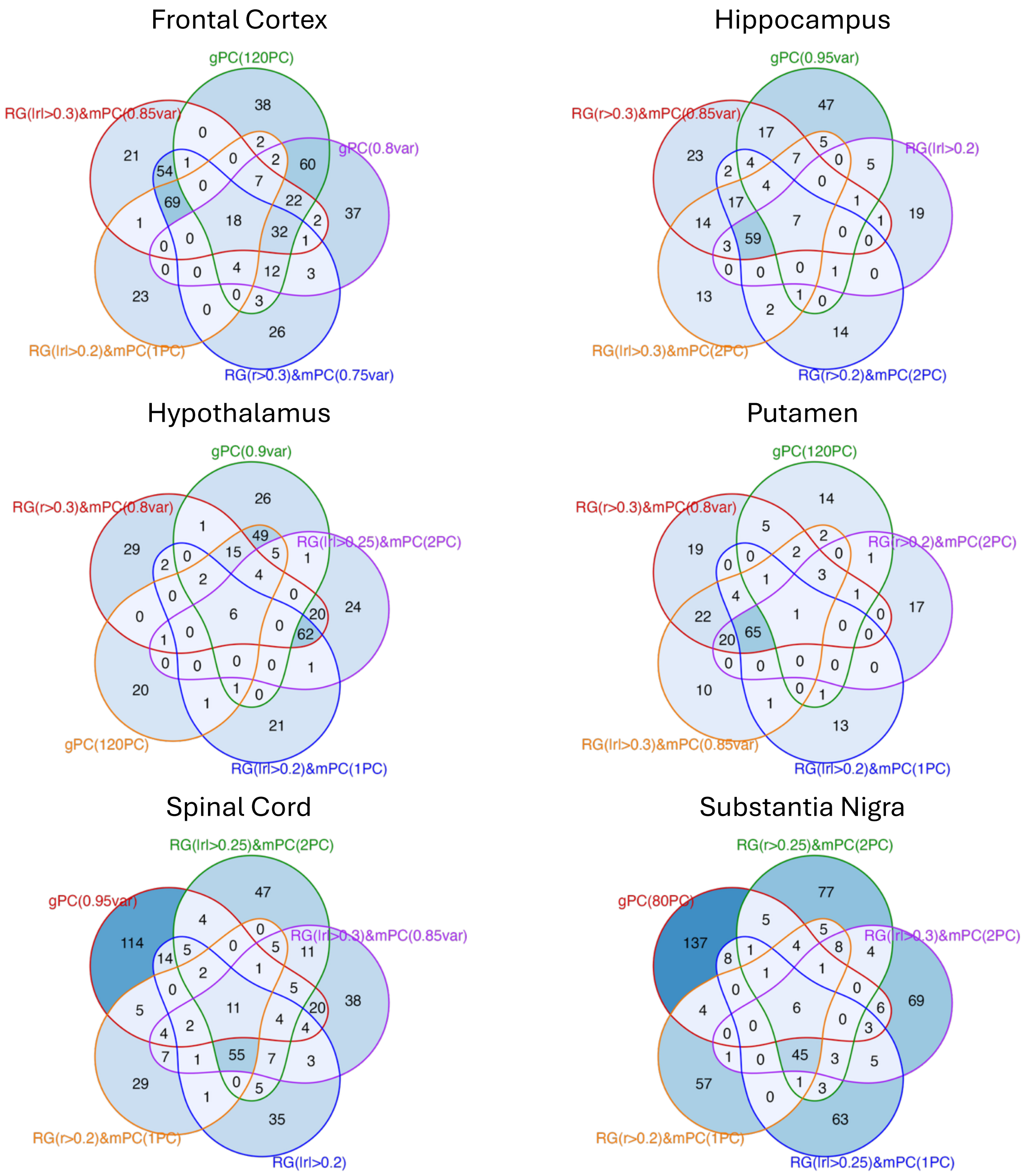

Figure S2. Top 5 feature selection methods based on the number of genes predicted with average CV r>0.5. The method that predicted the most genes is annotated in red, the second in green, the third in purple, the fourth in blue, and the fifth in orange.

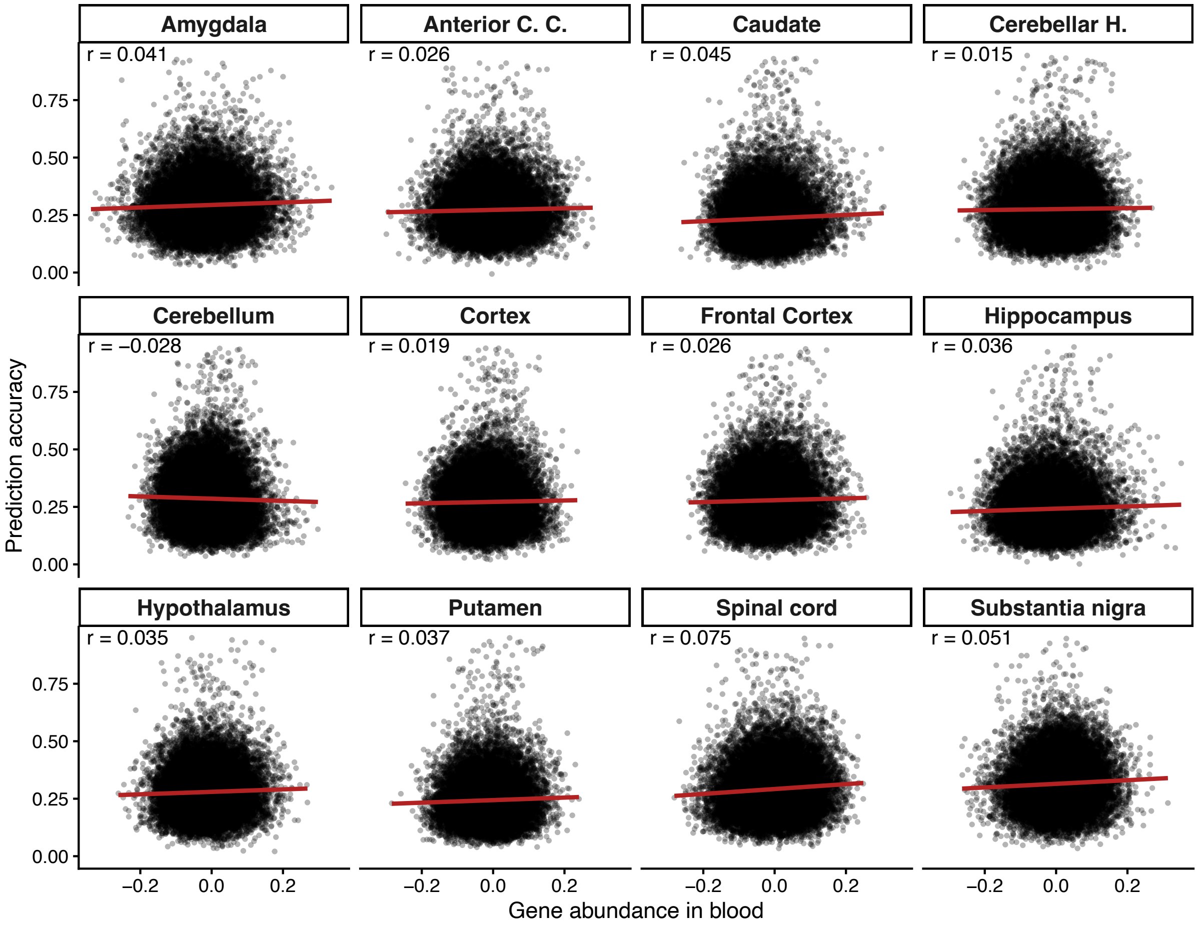

Figure S3. Relationship between blood gene expression abundance and prediction accuracy across brain regions. Scatter plots show the association between gene expression abundance measured in blood (x-axis; mean expression across samples) and prediction accuracy (y-axis; cross-validation correlation coefficient, CV r) for each brain region. Each point represents a gene. A linear regression line with 95% confidence interval is shown in red. The Pearson correlation coefficient between gene abundance and prediction accuracy is displayed in each panel.

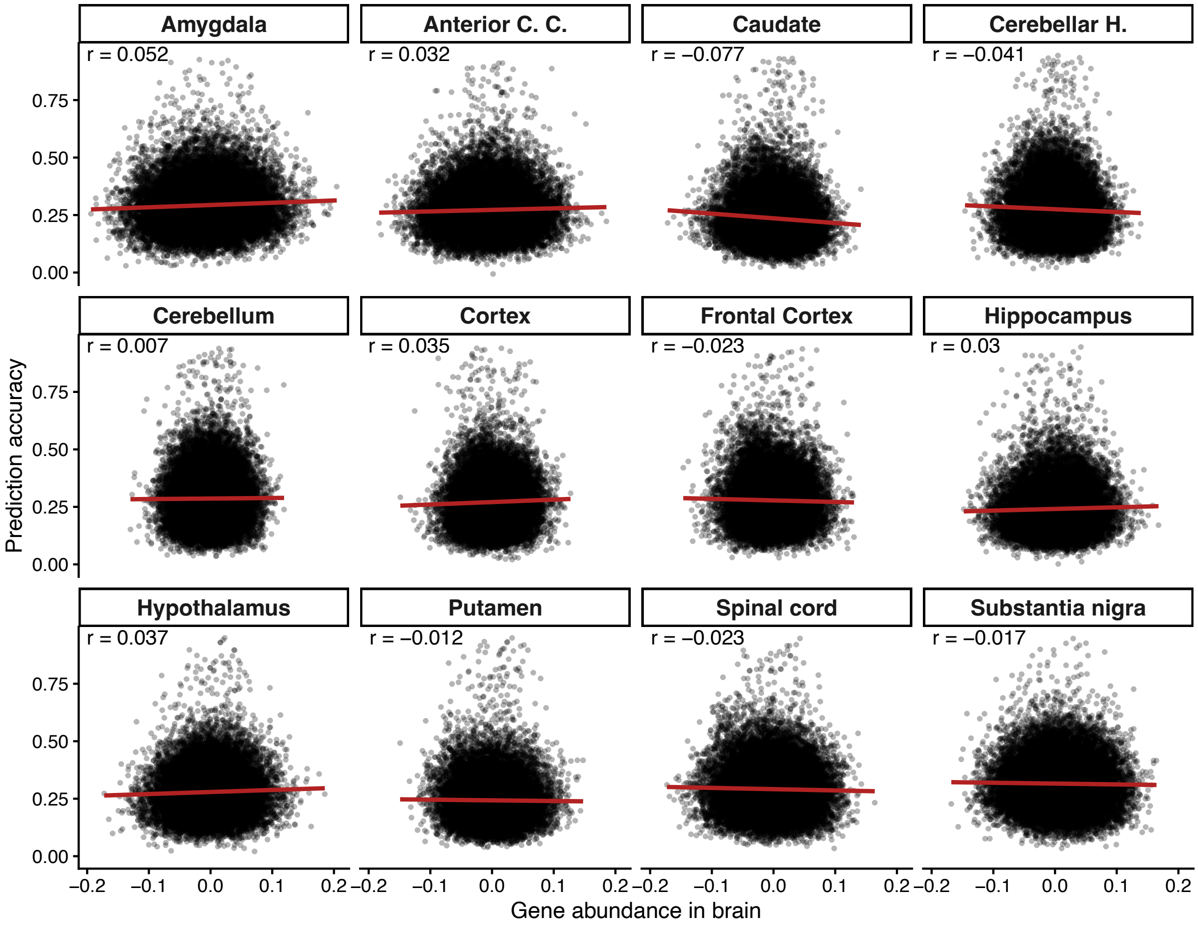

Figure 4. Relationship between brain gene expression abundance and prediction accuracy across brain regions. Scatter plots show the association between gene expression abundance measured in the corresponding brain tissue (x-axis; mean expression across samples) and prediction accuracy (y-axis; cross-validation correlation coefficient, CV r) for each brain region. Each point represents a gene. A linear regression line with 95% confidence interval is shown in red. The Pearson correlation coefficient between gene abundance and prediction accuracy is displayed in each panel.

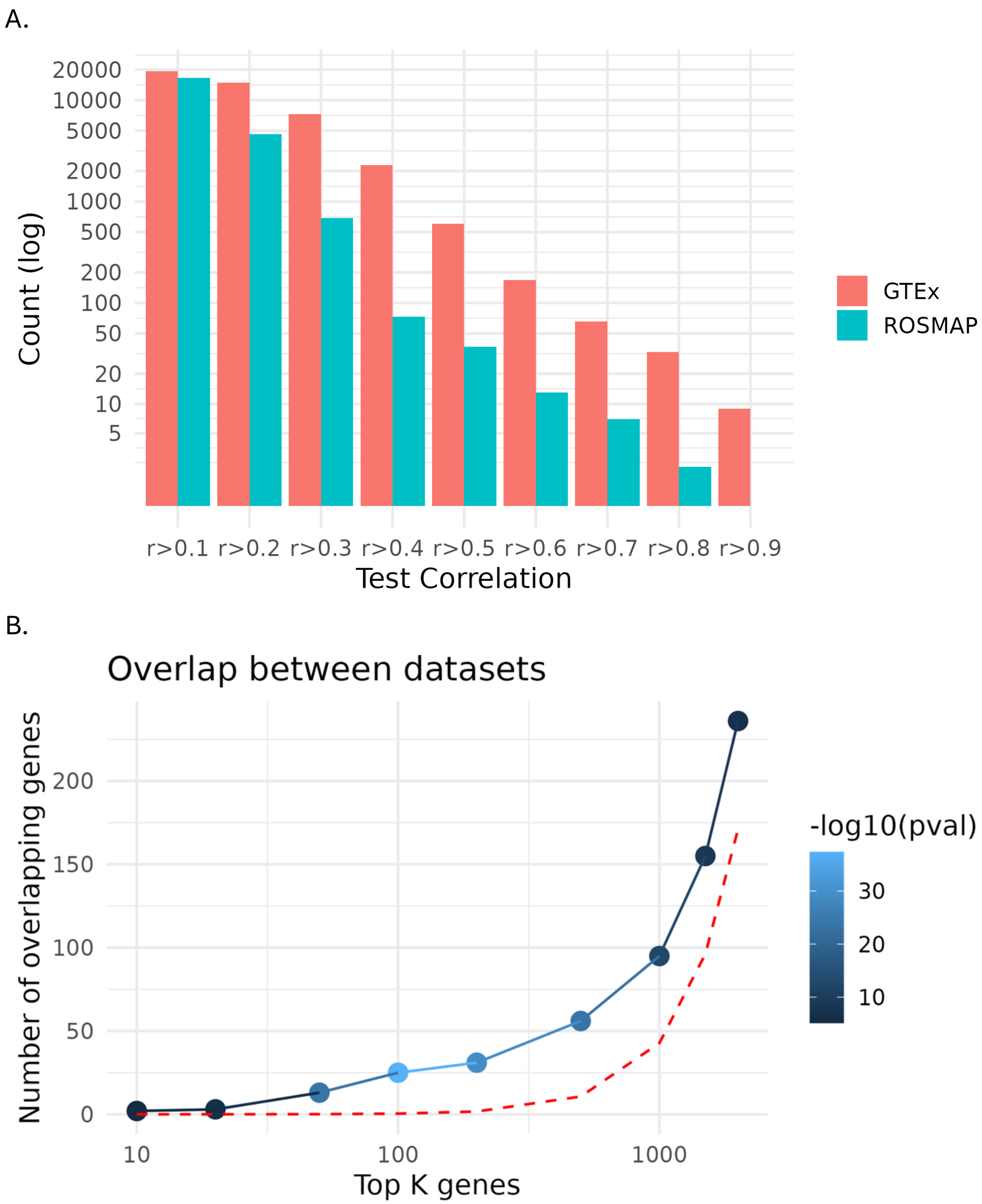

Figure S5. Comparison of gene prediction performance between GTEx and ROSMAP. (A) Number of genes predicted across correlation thresholds ($r > 0.1$ to $r > 0.9$). (B) Overlap of top-ranked genes across datasets at increasing cutoffs; points are colored by $-\log_{10}(p)$, and the red dashed line indicates the expected overlap under the null. Observed overlaps exceed expectation at all thresholds.

BBscore (layer=1)

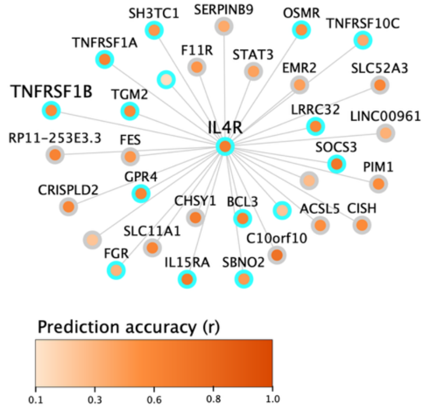

CERAD (layer=1)

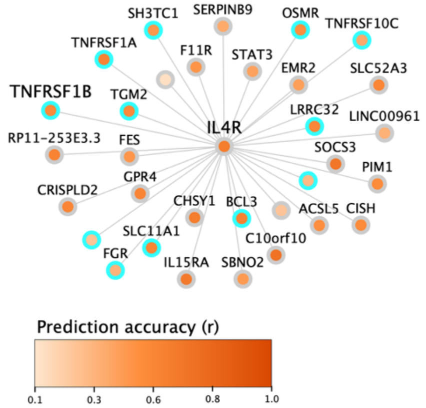

CDR (layer=1)

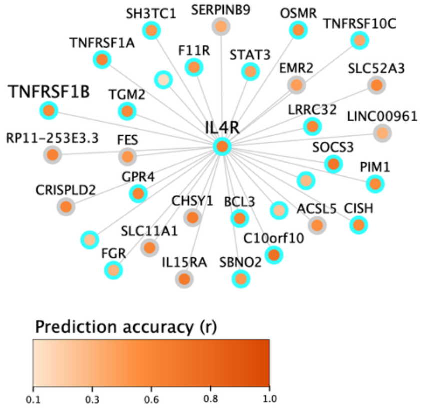

Plaque (layer=1)

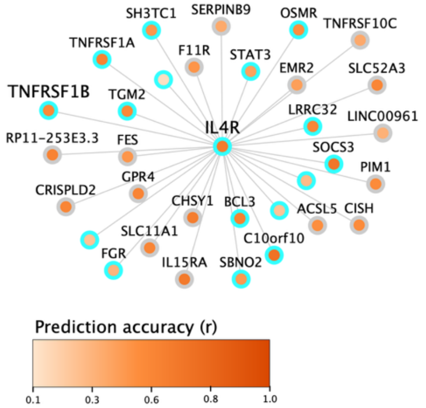

BBscore (layer=2)

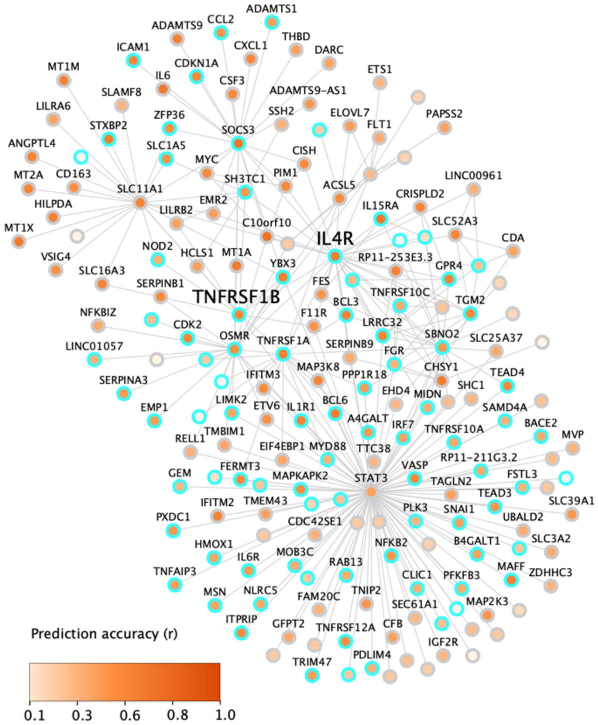

CERAD (layer=2)

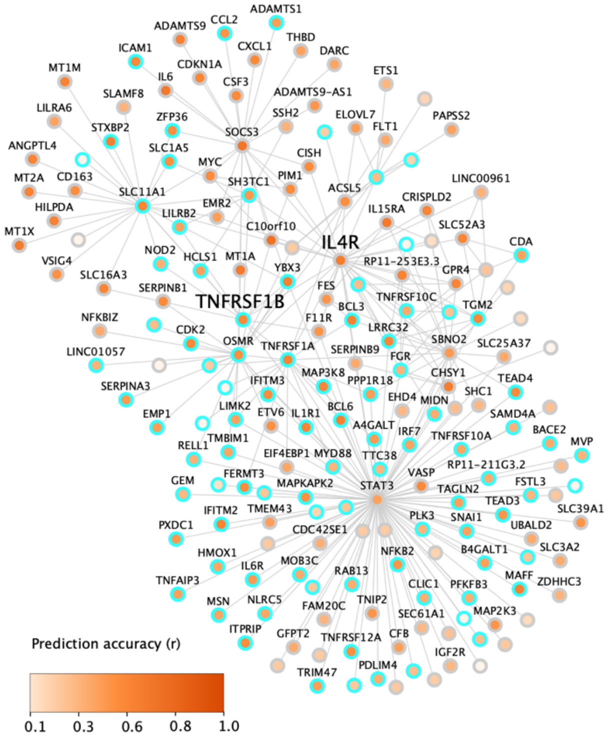

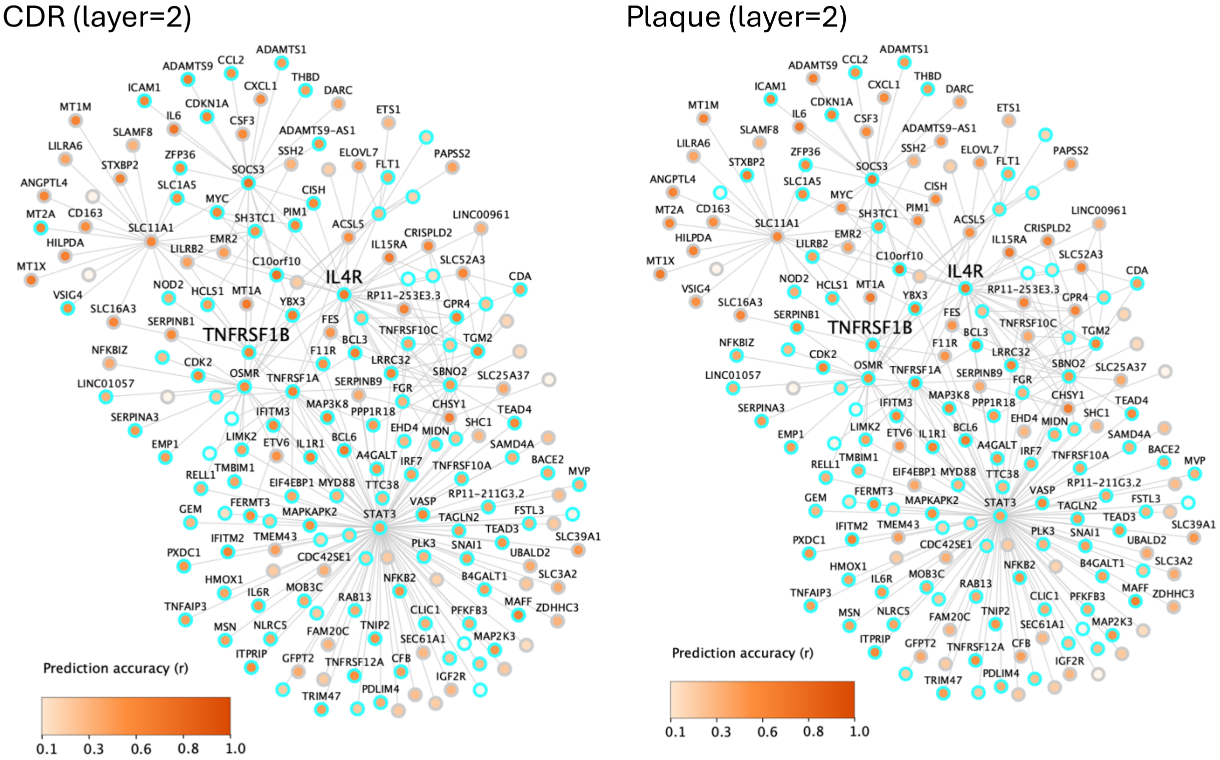

Figure S6. Co-expression networks of *IL4R* in the hippocampus across AD-related traits. Each panel shows a trait-specific co-expression network centered on *IL4R*, with varying network depths (layer=1 and layer=2). Nodes represent genes, node color reflects prediction accuracy (darker indicates higher accuracy), and nodes with cyan borders are associated with AD based on distinct traits, including Clinical Dementia Rating (CDR), Braak & Braak score (bbscore), CERAD score, and plaque density in AD patients versus controls.

BBscore (layer=1)

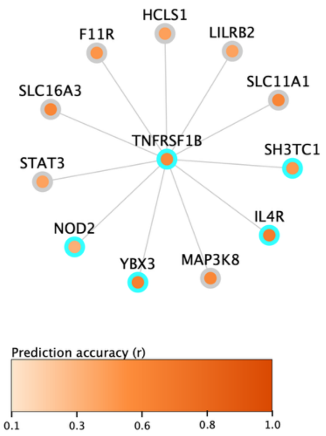

CERAD (layer=1)

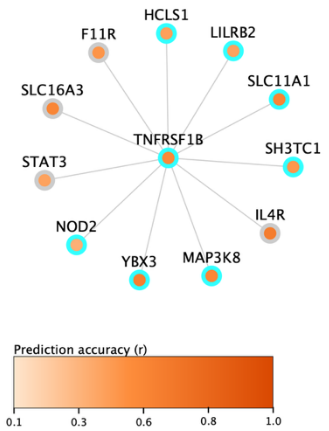

CDR (layer=1)

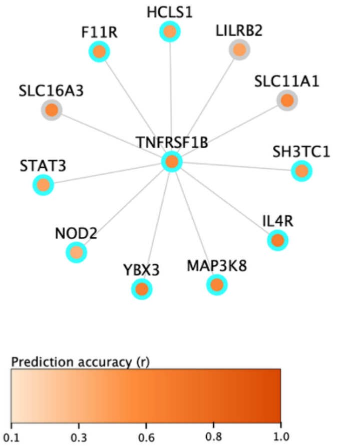

Plaque (layer=1)

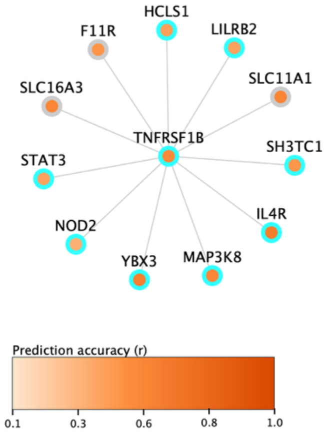

BBscore (layer=2)

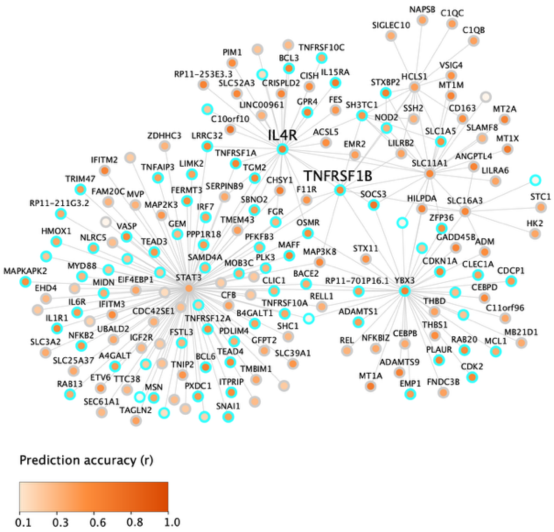

CERAD (layer=2)

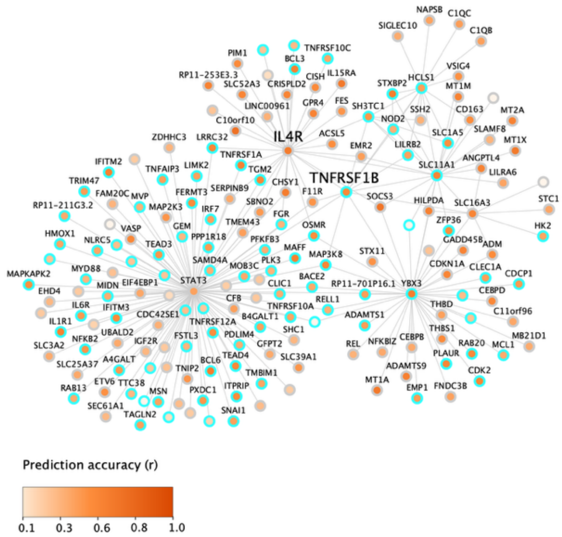

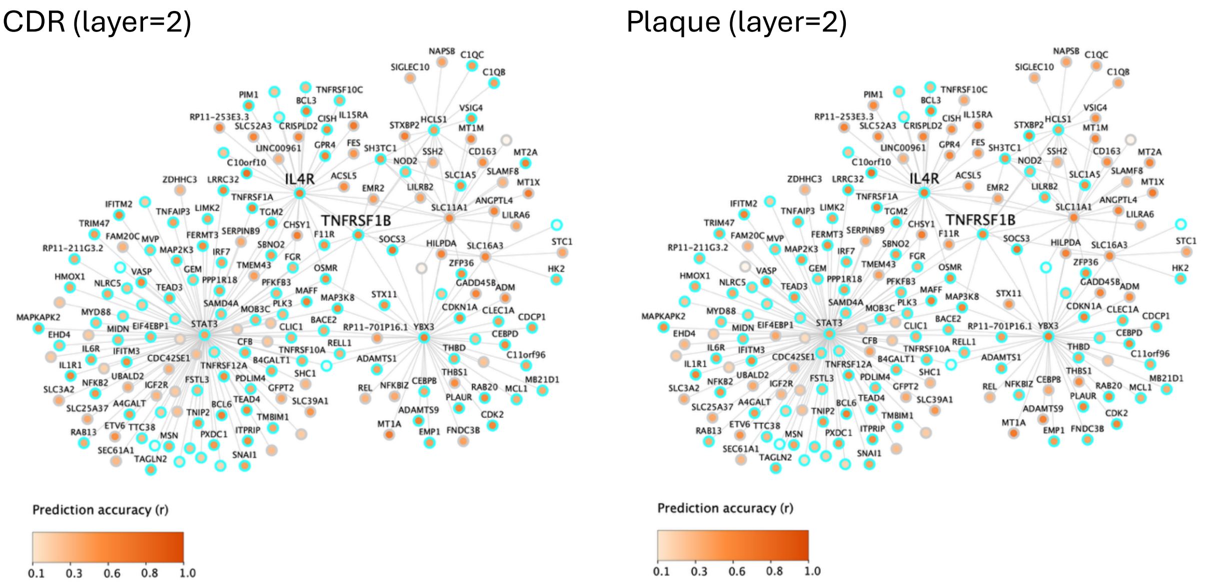

Figure S7. Co-expression networks of *TNFRSF1B* in the hippocampus across AD-related traits. Each panel shows a trait-specific co-expression network centered on *TNFRSF1B*, with varying network depths (layer=1 and layer=2). Nodes represent genes, node color reflects prediction accuracy (darker indicates higher accuracy), and nodes with cyan borders are associated with AD based on distinct traits, including Clinical Dementia Rating (CDR), Braak & Braak score (bbscore), CERAD score, and plaque density in AD patients versus controls.

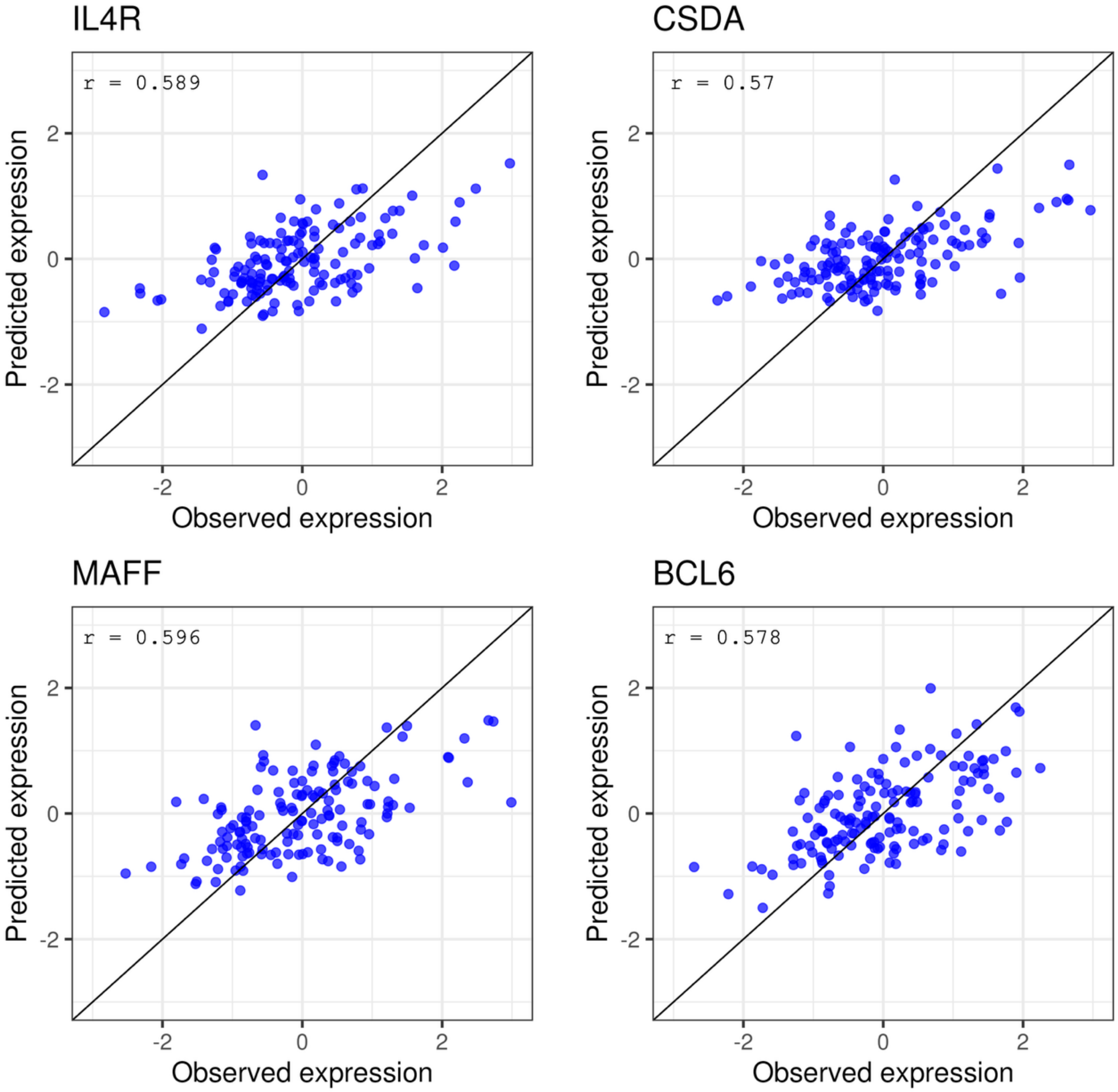

Figure S8. Observed versus predicted gene expression in the hippocampus for the four most predictable genes among the 1,000 AD key drivers: IL4R, CSDA, MAFF, and BCL6. Each panel shows sample-level scatter plots with observed expression (x-axis) and predicted expression (y-axis) from blood expression data across individuals in the GTEx. Pearson correlation coefficients (CV r) are indicated. These results highlight the strong predictive performance of our model for key AD-relevant genes.
