# Supplementary material for: Leveraging machine learning and network biology approaches to predict brain gene expression from blood transcriptomes
Source: Gigascience. 2026 May 18;15:giag058. doi: 10.1093/gigascience/giag058 (PMC13201078; doi:10.1093/gigascience/giag058)

GigaScience

Leveraging Machine Learning and Network Biology to Uncover Blood Biomarkers of Brain Gene Expression

--Manuscript Draft--

**Manuscript Number:**	GIGA-D-25-00434
**Full Title:**	Leveraging Machine Learning and Network Biology to Uncover Blood Biomarkers of Brain Gene Expression
**Article Type:**	Research
**Funding Information:**	National Institute on Aging (UH2AG083258) — Prof Bin Zhang
	National Institute on Aging (R01AG085182) — Prof Bin Zhang
	National Institute on Aging (RF1AG074010) — Prof Bin Zhang
	National Institute on Aging (RF1AG054014) — Prof Bin Zhang
	National Institute on Aging (U01AG046170) — Prof Bin Zhang
	National Institute on Aging (R01AG068030) — Prof Bin Zhang
**Abstract:**	Blood-based biomarkers offer a promising non-invasive strategy for detecting disease-related changes and monitoring tissue and organ health, including brain function. While recent studies have leveraged blood transcriptomic data to predict gene expression in the brain, existing models generally suffer from poor accuracy, limiting their translational utility. Here, we present an integrative prediction system (IPS) that combines machine learning with network biology to predict region-specific brain gene expression from blood transcriptomic data. Our framework integrates global blood transcriptomic signals, co-expression network features, and inter-tissue gene–gene interaction data linking blood genes to their target genes in the brain. Applied to the Genotype-Tissue Expression (GTEx) cohort, IPS substantially outperforms existing approaches in both the number and accuracy of brain genes that can be reliably predicted from blood. Notably, immune-related blood genes emerged as key contributors to model performance, underscoring the systematic interplay between peripheral immune signaling and central nervous system. These findings highlight the potential of blood-based transcriptomic models as scalable, non-invasive tools for studying brain function and developing diagnostic and prognostic biomarkers for neurological and psychiatric disorders.
**Corresponding Author:**	Cigdem Sevim Bayrak<br>Icahn School of Medicine at Mount Sinai<br>New York, UNITED STATES
**Corresponding Author Secondary Information:**	
**Corresponding Author's Institution:**	Icahn School of Medicine at Mount Sinai
**Corresponding Author's Secondary Institution:**	
**First Author:**	Cigdem Sevim Bayrak
**First Author Secondary Information:**	
**Order of Authors:**	Cigdem Sevim Bayrak
	Cigdem Sevim Bayrak
	Qi Zeng
	Marjan Ilkov
	Scott J Russo

Powered by Editorial Manager® and ProduXion Manager® from Aries Systems Corporation

	Minghui Wang
	Bin Zhang
**Order of Authors Secondary Information:**	
**Additional Information:**	
**Question**	**Response**
Are you submitting this manuscript to a special series or article collection?	No
**Experimental design and statistics**<br><br>Full details of the experimental design and statistical methods used should be given in the Methods section, as detailed in our Minimum Standards Reporting Checklist. Information essential to interpreting the data presented should be made available in the figure legends.<br><br>Have you included all the information requested in your manuscript?	Yes
**Resources**<br><br>A description of all resources used, including antibodies, cell lines, animals and software tools, with enough information to allow them to be uniquely identified, should be included in the Methods section. Authors are strongly encouraged to cite Research Resource Identifiers (RRIDs) for antibodies, model organisms and tools, where possible.<br><br>Have you included the information requested as detailed in our Minimum Standards Reporting Checklist?	Yes
**Availability of data and materials**<br><br>All datasets and code on which the conclusions of the paper rely must be either included in your submission or deposited in publicly available repositories (where available and ethically appropriate), referencing such data using	Yes

a unique identifier in the references and in the “Availability of Data and Materials” section of your manuscript.<br><br>Have you have met the above requirement as detailed in our Minimum Standards Reporting Checklist?	
GigaScience has policies and guidelines in place for the use of generative AI-writing tools such as ChatGPT. If you have used such writing tools to assist with writing the manuscript this must be declared and cited in the text. Authors should not list AI-writing tools and other AI-assisted technologies as an author or co-author and should acknowledge that they are fully responsible for text generated or refined by AI-writing tools.<p><br><br>A summary of use (particularly in the introduction or among methods) needs to be included at the end of the paper, and the outputs should also be included as a supplementary file hosted in GigaDB or other open repositories. Please read our guidelines for more information. <p><br><br>By submitting to GigaScience, you are aware of the journal's AI-writing tools policy, and if you have declared use of such tools below, you have acknowledged this where appropriate in your manuscript and have made a summary of use and outputs available. <p><br><b>AI-assisted writing tools have been used in the preparation of this manuscript?	No

Manuscript

Click here to access/download;Manuscript;Maintext_10-15-25.docx

Leveraging Machine Learning and Network Biology to Uncover Blood Biomarkers of Brain Gene Expression

Cigdem Sevim Bayrak[1,2], Qi Zeng[1,2], Marjan Ilkov[1,2], Scott J Russo[3,4,5], Minghui Wang[1,2], Bin Zhang[1,2]

[1] Department of Genetics and Genomic Sciences, Icahn School of Medicine at Mt Sinai, New York, NY, USA

[2] Mount Sinai Center for Transformative Disease Modeling, Icahn School of Medicine at Mount Sinai, New York, NY, USA

[3] Friedman Brain Institute, Icahn School of Medicine at Mount Sinai, New York, NY, USA

[4] Nash Family Department of Neuroscience, Icahn School of Medicine at Mount Sinai, New York, NY, USA

[5] Brain and Body Research Institute, Icahn School of Medicine at Mount Sinai, New York, NY, USA

Abstract

Blood-based biomarkers offer a promising non-invasive strategy for detecting disease-related changes and monitoring tissue and organ health, including brain function. While recent studies have leveraged blood transcriptomic data to predict gene expression in the brain, existing models generally suffer from poor accuracy, limiting their translational utility. Here, we present an integrative prediction system (IPS) that combines machine learning with network biology to predict region-specific brain gene expression from blood transcriptomic data. Our framework integrates global blood transcriptomic signals, co-expression network features, and inter-tissue gene–gene interaction data linking blood genes to their target genes in the brain. Applied to the Genotype-Tissue Expression (GTEx) cohort, IPS substantially outperforms existing approaches in both the number and accuracy of brain genes that can be reliably predicted from blood. Notably, immune-related blood genes emerged as key contributors to model performance, underscoring the systematic interplay between peripheral immune signaling and central nervous system. These findings highlight the potential of blood-based transcriptomic models as scalable, non-invasive tools for studying brain function and developing diagnostic and prognostic biomarkers for neurological and psychiatric disorders.

Introduction

Blood biomarkers are emerging as a minimally invasive approach to investigate both neurodegenerative disease pathology and normal brain function. Neurodegenerative diseases (NDDs), including Alzheimer's disease (AD) and Parkinson's disease (PD), affect millions globally and account for approximately 15% of the population. With the global population aging, the burden of these disorders is expected to rise significantly [1]. Early and precise diagnosis is essential for effective prevention and treatment but remains challenging in clinical settings.

While neuroimaging and cerebrospinal fluid (CSF) biomarkers have advanced in vivo characterization of disease processes, their use is limited by cost, limited accessibility, and invasiveness[2]. Blood-based biomarkers offer a minimally invasive, cost-effective alternative for detecting disease-related changes and monitoring brain health across the lifespan[3], with the potential to transform both clinical practice and research into normal and pathological brain aging.

The brain, the most complex organ in the human body, consists of billions of neurons forming trillions of connections with each other[4]. Characterizing the brain transcriptome is essential for understanding the molecular mechanisms that underpin neurological disorders. However, the limited availability of human brain tissue samples presents a significant challenge[5]. While gene expression patterns in the brain are largely consistent across individuals, distinct transcriptional profiles are observed across different tissues, with tissue-specific characteristics primarily determined by a select group of genes[6, 7]. Notably, studies have shown a strong correlation between gene expression profiles in the brain and blood[8], with co-expression networks of genes maintained across both tissues[9]. This strong correlation, along with the ease of access and cost-effectiveness of blood samples, makes the blood transcriptome a valuable resource for studying neurological disorders. Hence, many research efforts focus on utilizing patient blood samples to identify gene signatures associated with these conditions[10-12]. Predicting brain gene expression using blood transcriptome data could significantly enhance our understanding of brain-specific gene activity and disease-related changes. Moreover, incorporating transcriptional profiles from various tissues could further improve the accuracy of predictions regarding gene expression in the brain.

Recent studies have leveraged blood transcriptome data to develop generalized, transcriptome-wide models for predicting brain expression data[13-15]. Given the high dimensionality of the data, principal component analysis (PCA) has been frequently utilized for feature selection. Although these methods may outperform genotype-based prediction methods for certain genes, their overall predictive accuracy remains limited. More advanced and targeted approaches are required for more accurately capturing the complex, tissue-specific interactions between peripheral and brain gene expression. To improve gene-specific prediction performance, we propose an Integrative Prediction System (IPS), which leverages a diverse set of feature selection algorithms to model the complex, tissue-specific relationships, rather than relying on a singular, genome-wide predictive framework. In this study, we apply both (i) unsupervised feature selection, where features are selected without reference to the target brain gene, and (ii) supervised feature selection, where features are selected based on their relationship with the target brain gene. Leveraging paired blood and brain expression data from the GTEx dataset (v.8)[16], this study seeks to enhance the accuracy of gene-specific predictions across twelve brain tissues by integrating features from both blood transcriptomic data and co-expression network models derived from the MEGENA network[17]. Our comparison of different feature sets and selection strategies demonstrates that different approaches predict distinct gene sets, highlighting the importance of using complementary methods to improve gene-specific predictions. This approach lays the groundwork for more precise, tissue-specific biomarkers and better understanding of gene expression dynamics across tissues. Prediction models based on peripheral tissue could extend their applicability beyond brain tissue and NDDs. When applied to other tissues, such as the heart and lungs, these models have the potential to provide valuable diagnostic and prognostic insights for a wide range of conditions.

Materials and Methods

A general workflow of this study is shown in Figure 1, and the details are explained below.

Dataset

We have downloaded the raw count of RNA-seq data from the GTEx (v8) database and normalized using trimmed mean of M-values normalization (TMM) method to adjust for sequencing library size difference[18]. The normalized gene expressions were then log2 transformed. We applied a linear model to adjust for the covariates including "SMCENTER" (collection sites), "SMRIN" (RNA integrity), "SMTSISCH" (ischemic time), "SMEXNCRT" (exonic rate), "SMRRNART" (rRNA rate), "SMNTERRT" (intergenic rate) and "SEX" (gender) and used the residuals from the regression model for downstream analysis. Next, we have prepared paired blood and brain transcriptome data for each brain tissue. Figure 2A shows the number of individuals with paired blood-brain data across different brain tissues.

Network models

In order to identify groups of closely co-expressed genes, we have generated tissue-specific co-expression networks utilizing the Multiscale Embedded Gene co-Expression Network Analysis (MEGENA)[17] R package. The gene expression matrix was permuted (n = 10) across the samples to calculate the false-positive rate (FPR) and the corresponding false discovery rate (FDR) for each correlation coefficient cutoff. An FDR threshold of 0.05 was then applied to determine the correlation coefficient cutoff that effectively filtered out insignificant correlations. The significant gene pairs were sorted by their absolute Pearson correlation coefficients. These sorted gene pairs were sequentially examined to determine if they could be placed on a three-dimensional topological sphere without intersecting other edges, a process known as the

planarity test. Multiscale clustering analysis (MCA) was applied on the resulting co-expression network, planar filtered network (PFN), to identify network clusters (e.g., gene modules) at various compactness resolutions. MCA divides the parent module into child modules by searching for an optimal partition based on Newman's modularity. Multiscale hub analysis was then performed by to identify nodes with significantly higher network connectivity compared to the randomly permuted planar networks ($P < 0.05$). Finally, PCA was applied on each module to determine module features.

Feature selection

Unsupervised feature selection: To reduce the dimensionality of the blood expression training data, principal component analysis (PCA) was applied using the prcomp() R function. We evaluated different number of principal components (e.g., 40, 80, 100, and 120 PCs) and explored various thresholds for the percentage of explained variance (e.g., 80%, 85%, 90%, and 95%).

Supervised feature selection: To select subsets of features that are highly correlated with the target brain gene expression data, we have applied correlation-based feature selection by Pearson's correlation measure using the cor() R function. We evaluated highly correlated features (e.g., $|\rho| > \{0.2, 0.25, \text{ and } 0.3\}$) as well as positively correlated features (e.g.,$\rho > \{0.2, 0.25, \text{ and } 0.3\}$).

Network features selection: To generate network-based features from the blood modules, eigengenes (the first principal component) of each module were calculated. To further explore the effectiveness of network features, we also explored using the top 2, 5, and 10 PCs and the PCs that explained 70%, 75%, 80% and 85% of the total variance of each module.

Training and prediction

To develop gene-specific prediction models, we applied 5-fold cross-validation, where in each fold, 4/5 of the data were used for training and 1/5 served as a hold-out test set. In each training subset, we select features by choosing (i) top PCs from principal component analysis on blood transcriptome data, (ii) highly correlated blood gene markers with target brain genes, (iii) top PCs from each network module, (iv) union of highly correlated blood gene markers and top PCs of blood gene expression network modules. Then, we performed both linear regression, using the lm() function, and elastic net modeling, using the glmnet() function, on the selected features in R version 4.2.0. To avoid overfitting, linear model was performed only when the number of selected features is less than 1000. For each gene, we have performed 70 different feature selection methods using various cutoffs and approaches as explained above. The performance of each model was evaluated by calculated average cross-validation correlation coefficient, *r*, between predicted and observed expression values in the corresponding hold-out (e.g., test) data from each fold.

Pathway enrichment

For functional analysis of the genes, we used the R package enrichR with the GO-Biological Process and Reactome databases [19-21].

Results

Distinct Information Captured by Supervised and Unsupervised Feature Selection

To identify the most effective feature selection mechanism for brain gene expression, we utilized four different approaches within the training sets from 5-fold cross validation (CV). We first applied principal component analysis (PCA) on the blood transcriptome data and used the top

principal components (PCs) as features for predicting gene expression in the brain tissues (i.e., unsupervised feature selection). The top 40, 80, 100, and 120 PCs as well as the PCs that explain 80%, 85%, 90%, and 95% of the variance (denotated as gPC(.)) were selected as features.

As an alternative approach, we also identified blood genes whose gene expression profiles were correlated with a target brain gene (i.e., supervised feature selection) by Pearson's correlation coefficient thresholds of $|r|>0.2$, 0.25, and 0.3, as well as $r>0.2$, 0.25, 0.3 (denoted as RG(.)). Thirdly, we extracted the module features by selecting the top 1, 2, 5, and 10 PCs, and the PCs that explained 70%, 75%, 80%, and 85% of the variance of each co-expressed gene module (denotated as mPC(.)). Lastly, we combined the module features with blood genes that were highly correlated with a given brain gene.

Prediction accuracy was assessed as the average correlation between the predicted brain gene expression values and the actual brain gene expression values across 5 folds. The number of predicted genes (with an average CV correlation coefficient, $r > 0.1$) varied by brain region and feature selection method. When using the top-performing method for each region, the number of predicted genes ranged from 10,583 in hippocampus to 15,253 in the cerebellum (PAXgene-preserved), with corresponding method-specific configurations noted in Table S1. The number of genes predicted with moderate accuracy ($r \geq 0.6$) ranged from 65 to 134, with the highest count observed in the cerebellum using a combination of correlation-based and module-derived features. High confidence predictions ($r \geq 0.9$) were achieved for 2 to 10 genes per region, again most frequently in the cerebellar hemisphere.

To more systematically evaluate prediction performance across accuracy thresholds, we quantified the number of predicted genes at nine r thresholds (0.1 through 0.9) across brain

regions (Fig. S1). When using only the top-performing feature selection method, the cerebellum (PAXgene-preserved) and cortex (PAXgene-preserved) showed the highest number of predicted genes with r > 0.1 (15,253 and 15,136, respectively), while the putamen and hippocampus showed the lowest (11,323 and 10,583, respectively). At r > 0.5, prediction counts ranged from 363 in the cerebellum to the fewest in the anterior cingulate cortex. At the highest accuracy threshold (r > 0.9), gene counts ranged from 10 in the cerebellar hemisphere to 2 in the anterior cingulate. When aggregating predictions from the top five feature selection methods, these numbers increased overall, with the substantia nigra and hypothalamus showing the most genes at r > 0.1 (18,335 and 18,100, respectively), and the hippocampus showing the least (16,373). For r > 0.5, the cerebellum again had the highest count (653), and the putamen the lowest (202). At r > 0.9, the cerebellar hemisphere had the most genes (13), and the anterior cingulate cortex the fewest (2). One contributing factor to the variation in predictive performance across brain regions may be the number of available paired samples, which is highest for the cortex, cerebellum, caudate, and cerebellar hemisphere (Figure 2A), potentially enhancing model robustness and prediction accuracy in these regions.

Overall, unsupervised principal component-based feature selection predicted a larger number of genes, but with lower accuracy (average CV r between 0.1 and 0.6), whereas correlation-based feature selection predicted fewer genes, but with much higher accuracy (r ≥0.6). Figure 2B shows these trends in the frontal cortex, and Figure 2C how different feature selection strategies contribute across accuracy thresholds, suggesting that integrating supervised and unsupervised approaches can improve predictive power.

When compared with the recently developed BrainGENIE method, which uses the top 40 blood PCs, our IPS approach consistently yielded a higher number of predicted genes (avg CV r >0.1)

across all brain tissues (Figure 3A). For example, in the cerebellum 15,247 genes were predicted by using the top 120 PCs (e.g., gPC(120PC)), whereas BrainGENIE predicted only 10,048 genes.

Blood Network Features Enhance the Accuracy of Predictions for Specific Genes

We have constructed a blood tissue co-expression network using MEGENA and determined the top PCs of each co-expression network module as module features (i.e., mPC(.)). We have determined the module eigengene (the first PC) as well the first 2, 5, and 10 PCs, and the PCs that explained 70%, 75%, 80%, and 85% of the variance of each module. The prediction accuracy for genes, ranging from 2,395 to 3,043, with an average CV correlation $r>0.1$, was increased by at least 5% across different brain regions when module features were used (Table S2). Figure 3B illustrates the genes that showed the most notable improvement in prediction accuracy within the frontal cortex.

We then assessed whether integrating features derived directly from the blood transcriptome (e.g., global features) with those derived from the blood network (e.g., module features) improves predictive accuracy for specific genes. This approach improved the prediction accuracy for a greater number of genes. Specifically, the accuracy of genes, ranging from 6,228 to 8,777, with an average CV $r>0.1$, was increased by at least 5% across all brain regions (Table S3). Figure 3C illustrates the genes that showed the most notable improvement in prediction accuracy within the frontal cortex.

Overall, the combination of module features and global features had the best performance by predicting half of the profiled genes with $r>0.1$, while global features alone accounted for 35% of the genes, and module features alone contributed to 15% across all the brain regions (Figure

4A). Figures 4B show the performance of different gene sets, highlighting the feature sets that yielded the best results for each. Utilizing different feature sets and feature selection approaches capture complementary, orthogonal information, each contributing to the prediction of different genes.

Gene-Specific Power of Different Feature Selection Methods

To evaluate the performance of all proposed feature selection methods, we identified the top 5 methods with the highest number of predicted genes with an average CV r>0.5. For 10/12 brain regions, the feature selection method predicting the most genes with r>0.5 was the combination of module and global features (Figure S1). The unsupervised (PCA) method applied on blood transcriptome was the top method for the remaining 2/12 brain regions (spinal cord and substantia nigra) and was the second best for the 9/12 regions. Interestingly the second-best method was the supervised feature selection for amygdala.

Prediction Performance of the Alzheimer's Disease Related Genes

To assess the predictive capacity of our models for brain expression of AD-related genes, we evaluated the prediction accuracy of the top 1,000 AD key drivers identified in postmortem para-hippocampal gyrus (PHG) samples from the Mount Sinai Brain Bank (MSBB) AD cohort[22]. Overall, 341 genes exhibited an average CV r>0.1, with 14 genes reaching r>0.5 in the hippocampus (Figure 5A-B). Among them, the IL-4 receptor (*IL4R*) gene demonstrated the highest prediction accuracy with average CV r of 0.61. *IL4R* is expressed on microglia and plays a crucial role in regulating microglial phenotype[23, 24]. It has been proposed that IL-4, a ligand for IL4R, may have a protective role in AD by regulating neuroinflammation and amyloid-beta pathology[25].

Another well predicted AD-related gene is the *TNFRSF1B* gene (avg CV r=0.51), which encodes a protein that is a member of the TNF-receptor superfamily. Genetic variants in *TNFRSF1B* have been associated with cognitive resilience in AD[26]. To further explore the functional context of these genes, we generated hierarchical co-expression networks centered on *IL4R* and *TNFRSF1B* in the hippocampus using the MSBB dataset. These networks were constructed at two levels (layer 1 and layer 2 from *IL4R* or *TNFRSF1B*), reflecting immediate and extended co-expression relationships. We assessed the functional relevance of genes withing each network layer by evaluating their predictive performance and association with AD pathology. Specifically, we examined the previously established AD associations of these network genes as identified through prior analyses of differential expression patterns across clinical traits, including Clinical Dementia Rating (CDR), Braak & Braak score (bbscore), CERAD score, and plaque density, in AD patients versus controls from the MSBB dataset[22]. To illustrate the model's performance, we highlighted a representative layer 2 network associated with CDR for *IL4R,* which is also closely connected to *TNFRSF1B,* in Figures 5C. These examples demonstrate that the genes that overlap with AD molecular signatures have good prediction performance (172/182 network genes were predicted with average CV r>0.1). Comprehensive results for all network layers and traits are provided in the Supplementary Figures. Full panels of predictive performance plots for *IL4R* (8 conditions: 2 layers × 4 traits) are shown in Figure S2, and corresponding analyses for *TNFRSF1B* are included in Figure S3. Additionally, to further validate the predictive accuracy of our model at the individual gene level, Figure S4 presents observed versus predicted gene expression values in the hippocampus for the four most predictable genes from the 1,000 AD key drivers list: *IL4R*, *CSDA*, *MAFF*, and BCL6. These plots highlight the model's ability to accurately capture gene-specific expression patterns relevant to AD biology.

Preservation of Age-Associated Expression Patterns in Predicted Gene Profiles

As a representative analysis, we examined whether our predictive models preserve age-associated gene expression patterns in the hippocampus. We first identified highly predictable genes (CV $r > 0.5$) and calculated the correlation between their observed expression and age (denoted as r_1). We then computed the correlation between predicted expression and age (r_2). To evaluate how well the predicted expression reflects age-related transcriptional variation, we calculated the correlation between r_1 and r_2 across genes. We observed a moderate positive correlation (Pearson's $r = 0.496$, $p < 2.2 \times 10^{-16}$), indicating that the model effectively preserves age-associated expression patterns. This suggests that predicted gene expression can serve as a reliable proxy for studying biologically meaningful age-related changes in the brain. The relationship between r_1 and r_2 is visualized in Figure 5E, highlighting the consistency between observed and predicted age associations. While this analysis was conducted in the hippocampus, the same approach can be extended to other brain regions as well as to other phenotypes beyond age, such as disease status.

Immune-related Genes Exhibit Greater Predictive Power

To assess biological functions of the accurately predicted genes ($r>0.5$) in each brain region, we performed pathway enrichment analysis using EnrichR[19]. On average 565 genes were predicted with $r>0.5$ across all brain regions, with the number of predicted genes ranging from 273 in the putamen to 976 in the substantia nigra (Figure 6A). We observed significant enrichment in immune-related pathways, including cytokine-mediated signaling, interleukin signaling, interferon signaling, and response to cytokine, across most brain regions (Figure 6B-C). The enrichment was strongest in the cortex (right cerebral frontal pole cortex, sampled at donor collection site and preserved in PAXgene fixative) and frontal cortex (right cerebral frontal

pole cortex, sampled at Miami Brain Bank and preserved as fresh frozen tissue). One potential explanation for this observation is the interaction between the brain and peripheral immune systems, which share common pathways and mechanisms, thereby leading to correlated changes in gene expression. Additionally, it is known that systemic immune cells, which can mobilize and directly infiltrate the brain parenchyma—influence gene expression in brain resident immune cells. responses.

Top Blood-based Predictors

To better understand the interactions between the blood biomarkers, and the brain genes, we assessed the most informative features of the brain genes with highest prediction accuracy. Initially, the top 10 brain genes exhibiting the highest accuracy in the frontal cortex were identified, including *NPIPB15, GSTM1, ENSG00000213058, RPS14P1, ENSG00000197582, GATD3, RPL13P12, TBC1D3, LINC01291, LOC102724159,* with average cross-validation correlation coefficients (CV r) ranging from 0.89 to 0.94. Similarly, the top 10 genes with the highest accuracy in the hippocampus were determined, namely *GSTM1, NPIPB15, GATD3, ENSG00000213058, RPS14P1, LINC01291, TBC1D3, ENSG00000262539, RPL13P12, LOC102724023,* with average CV r values between 0.86 to 0.95. Subsequently, for each gene, the top blood biomarkers were identified based on their correlation with the target brain gene ($|r|>0.2$) within each cross-validation fold. The top blood-based predictors were defined as those selected in at least two out of five CV folds. Pathway enrichment analysis was then performed on these top features (Figure 7). The results showed that the blood predictors for the genes *GATD3* and *LOC102724159* were significantly enriched in immune response-related pathways, including immunoglobulin-mediated immune response and antigen processing, which might suggest a role in immune response. The blood predictors for *GSTM1* and *NPIPB15* were enriched in pathways

associated with muscle contraction. Additionally, the blood predictors of the gene *TBC1D3*, a gene promoting dendritic arborization and protracting the pace of synaptogenesis [27], were enriched in the synaptic signaling pathways, while those for *RPL13P12* were enriched in biosynthetic process. Finally, the blood predictors for *RPS14P1* were enriched in pathways related to neutrophil degranulation and innate immune system. These results highlight the complex interactions between peripheral biomarkers and brain functions.

Discussion

Understanding the molecular processes in the brain is crucial for improving our knowledge of brain disorders, as gene expression in the brain plays an important role in the pathogenesis of both neurological (i.e., neurodegenerative) and psychiatric diseases. However, direct investigation of brain tissue remains limited due to the invasive nature of sample collection. Consequently, there is a pressing need for non-invasive biomarkers that can serve as reliable proxies for brain gene expression.

In this study, we explored the potential of blood-derived transcriptomic signatures to predict brain gene expression. Blood samples offer a minimally invasive and widely accessible source, making them an attractive alternative for large-scale studies and longitudinal monitoring. By integrating blood transcriptome data with a variety of feature selection techniques, we developed gene-specific predictive models. This gene-specific approach enables the identification of blood biomarkers that are closely tied to specific molecular processes in the brain, offering improved interpretability and potential clinical utility.

Despite promising results, several challenges remain. One key challenge is the heterogeneity of whole blood. The complex interactions between different cell types could dilute or conceal the

signals that are informative of brain gene activity. Incorporating cell-type deconvolution methods may help improve the signal-to-noise ratio and enhance model performance.

Another major limitation is the lack of paired transcriptome data from brain and blood tissues within the same individuals. The availability of such matched samples would enable more robust validation of the predictive models and offer a clearer understanding of cross-tissue gene expression relationships. While we leveraged large-scale, publicly available datasets for model training and evaluation, future efforts should prioritize collecting matched multi-tissue datasets, particularly in specific disease contexts, to support more rigorous benchmarking.

Additionally, although immune-related genes emerged as strong predictors of brain gene expression, the directionality and causality of these associations remain to be clarified. It is plausible that peripheral immune activity reflects or influences neuroinflammatory processes, which are increasingly recognized as key contributors to neurodegenerative disease. Indeed, controlled studies in rodents have now shown the dynamic interactions between peripheral immune compartments and the brain which support brain-body directionality and causality (PMID: 38326622). Further investigation into the shared regulatory mechanisms between the immune system and the brain will be essential to untangle these relationships.

Our findings underscore the feasibility of using blood-based transcriptomic models to infer brain gene expression and lay the groundwork for future diagnostic and prognostic tools. If validated in clinical cohorts, these models could be employed to identify early molecular signatures of neurological disorders such as AD, PD, and major depressive disorders. Moreover, they may enable non-invasive monitoring of disease progression or response to therapy, offering valuable insights for precision medicine.

Looking ahead, integrating additional layers of omics data—such as epigenetic modifications, proteomics, and metabolomics—may further refine prediction accuracy and enhance biological relevance. Incorporating longitudinal blood samples could also reveal dynamic transcriptomic changes associated with disease progression or treatment. Ultimately, expanding our understanding of peripheral-brain tissue interactions has the potential to bridge a gap in neurodegenerative research and improve clinical outcomes through earlier and more precise intervention.

Availability and requirements

Project name: Blood Biomarkers of Brain Gene Expression

Project home page: https://gitlab.com/csbayrak/ips

Operating system(s): Platform independent

Programming language: R

Other requirements: R 4.2 or higher

License: MIT

Data and code availability

The gene expression data and the associated sample information are publicly available at the GTEx portal (https://www.gtexportal.org/home/datasets). Specifically, we used bulk tissue expression from GTEx Analysis V8, including the gene read counts file GTEx_Analysis_2017-06-05_v8_RNASeQCv1.1.9_gene_reads.gct.gz (https://www.gtexportal.org/home/downloads/adult-gtex/bulk_tissue_expression#bulk_tissue_expression-gtex_analysis_v8-rna-seq) and the associated meta files (https://www.gtexportal.org/home/downloads/adult-

gtex/metadata#metadata-gtex_analysis_v8-metadata_files) [16]. The code and input files for an illustrative example are available on GitLab (https://gitlab.com/csbayrak/ips) and a snapshot of the project has been archived on Zenodo https://doi.org/10.5281/zenodo.17477449.

Acknowledgments

This work was financially supported in parts by grants from the National Institutes of Health (NIH)/National Institute on Aging (UH2AG083258, R01AG085182, RF1AG074010, RF1AG054014, U01AG046170, and R01AG068030).

This work was supported in part through the computational and data resources and staff expertise provided by Scientific Computing and Data at the Icahn School of Medicine at Mount Sinai and supported by the Clinical and Translational Science Awards (CTSA) grant UL1TR004419 from the National Center for Advancing Translational Sciences. Research reported in this publication was also supported by the Office of Research Infrastructure of the National Institutes of Health under award number S10OD026880 and S10OD030463. The content is solely the responsibility of the authors and does not necessarily represent the official views of the National Institutes of Health.

We gratefully acknowledge Yusra Chowdhury and Sophia DeGregorio (Stuyvesant High School, New York, NY), and Efe Guner (Walt Whitman High School, Bethesda, MD) for their contributions to both the preliminary and downstream stages of data analysis.

Author contributions

C.S.B. (Conceptualization, Investigation, Formal Analysis, Software, Methodology, Validation, Data Curation, Writing - Original Draft Preparation, Visualization), Q.Z. (Formal Analysis), M.I. (Data Curation), M.H.W. (Data Curation), S.J.R. (Supervision, Writing - Review & Editing), B.Z.

(Conceptualization, Project Administration, Funding Acquisition, Writing - Review & Editing). All authors reviewed and approved the final manuscript.

Declaration of interests

The authors declare no competing interests.

Figure Captions

Figure1. General framework of the Integrative Prediction System (IPS) for predicting brain gene expression using paired blood and brain transcriptome data from the GTEx dataset. The prediction models have been trained on paired blood and brain expression data from the GTEx dataset for each brain tissue via 5-fold cross validation. Predictive features have been generated (i) taking the top principal components, (ii) taking the highly correlated features with the target brain gene, (iii) taking the top principal components of the MEGENA modules of blood tissue. For training, linear regression and elastic models were used. The model prediction accuracy was estimated by calculating the average correlation between predicted and observed brain gene expression values over five folds.

Figure2. Sample overlap and gene prediction performance across feature selection methods. (A) Upset plot showing the number of individuals with paired whole blood and brain tissue samples in GTEx. The plot indicates that more than 100 subjects have both whole blood and brain tissue sample across all brain regions. **(B)** Number of genes predicted at various accuracy cutoffs using different feature selection methods. The x-axis shows the average correlation between the actual and predicted frontal cortex expression derived from blood tissue. gPC(.) represents the features selected by PCA from the blood transcriptome data, mPC(.) represents the module features selected by PCA, and RG(.) represents the features selected based on correlation with target brain

gene. IPS indicates the number of genes when best feature selection method was considered per gene. **(C)** Number of predicted genes by different feature selection strategies. The y-axis indicates the number of predicted genes in the frontal cortex. Each plot shows the number of predicted genes at varying levels of prediction accuracy, measured by the average cross-validation correlation, r.

Figure3. Comparative performance and accuracy gains of feature selection strategies for brain gene expression prediction. (A) Comparison of the performance of the proposed method, IPS, with existing approach. The x-axis shows the number of genes predicted with an average CV r>0.1 when using the top 120 PCs from blood transcriptome, BrainGenie (top 40 PCs), and when integrating the results from all 70 feature selection approaches proposed in this study (i.e., IPS). **(B)** Genes showing the greatest improvement in prediction accuracy (>500%) when using only module features compared to using only global features in the frontal cortex. **(C)** Genes showing the greatest improvement in prediction accuracy (>1000%) when using a combination of global and module features versus global features alone in the frontal cortex.

Figure4. Performance of different feature sets. (A) The percentage of genes for which each feature set performed best across different brain regions. **(B)** Performance of different feature selection strategies in the frontal cortex, measured by correlation, for various genes.

Figure5. Prediction performance and functional relevance of AD key driver genes in the hippocampus. (A) Cumulative number of top 1,000 Alzheimer's disease (AD) key driver genes achieving varying levels of prediction accuracy in the hippocampus, measured as the average cross-validated correlation (r) between observed and predicted expression. **(B)** Distribution of gene counts across defined prediction accuracy thresholds, highlighting how many genes fall within each performance range. **(C)** Co-expression network of IL4R in the hippocampus. Node color reflects prediction accuracy (darker indicates higher accuracy), and nodes with cyan borders are

associated with AD based on Clinical Dementia Rating (CDR). **(D)** Correlation between age-association patterns based on observed vs. predicted gene expression for genes with prediction accuracy CV $r > 0.5$ in the hippocampus. Each point represents a gene; the x-axis shows correlation with age based on observed expression, and the y-axis shows correlation with age based on predicted expression.

Figure6. Analysis of accurately predicted brain genes (r>0.5). (A) Number of genes predicted with r>0.5 accuracy across different brain genes. UpSet plot highlights the number of genes that are accurately predicted in multiple brain regions. **(B)** Top canonical pathways of the accurately predicted genes in each brain region. The shade of the color indicates the significance of enrichment (Pvalue). **(C)** Top gene ontology biological processes of the accurately predicted genes in each brain region.

Figure7. Analysis of the top blood-based predictors for brain genes selected based on the top 10 highest prediction accuracies. Pathway enrichment analysis of the top blood biomarkers for genes **(A)** in the frontal cortex and **(B)** in the hippocampus. Blood-based predictors were selected based on their correlation with target brain genes (|r|>0.2) selection in at least 2 out of 5 CV folds. The color intensity represents the significance of the enrichment.

Supplemental information

Table S1. Number of predicted genes at various accuracy cutoffs

Table S2. Number of genes with greater prediction accuracy (>5%) when using only module features compared to global features

Table S3. Number of genes with greater prediction accuracy (>5%) when using combined features compared to global features

Figure S1. Prediction performance across brain regions and feature selection combinations. Each panel displays the number of predicted genes (y-axis) at increasing prediction accuracy thresholds (x-axis, cross-validation correlation coefficient r) for a given number of combined feature selection methods (from 1 to 5). Each line represents a different brain region.

Figure S2. Top 5 feature selection methods based on the number of genes predicted with average CV r>0.5. The method that predicted the most genes is annotated in red, the second in green, the third in purple, the fourth in blue, and the fifth in orange.

Figure S3. Co-expression networks of IL4R in the hippocampus across AD-related traits

Figure S4. Co-expression networks of TNFRSF1B in the hippocampus across AD-related traits

Figure S5. Observed versus predicted gene expression in the hippocampus for the four most predictable genes among the 1,000 AD key drivers: IL4R, CSDA, MAFF, and BCL6

References

1. Feigin, V.L., et al., *The global burden of neurological disorders: translating evidence into policy.* Lancet Neurol, 2020. **19**(3): p. 255-265.
2. Alcolea, D., et al., *Blood Biomarkers in Neurodegenerative Diseases: Implications for the Clinical Neurologist.* Neurology, 2023. **101**(4): p. 172-180.
3. Oh, H.S., et al., *Organ aging signatures in the plasma proteome track health and disease.* Nature, 2023. **624**(7990): p. 164-172.
4. Hawrylycz, M.J., et al., *An anatomically comprehensive atlas of the adult human brain transcriptome.* Nature, 2012. **489**(7416): p. 391-399.
5. Danner, B., et al., *Brain banking in the United States and Europe: Importance, challenges, and future trends.* J Neuropathol Exp Neurol, 2024. **83**(4): p. 219-229.
6. Mele, M., et al., *Human genomics. The human transcriptome across tissues and individuals.* Science, 2015. **348**(6235): p. 660-5.
7. Sonawane, A.R., et al., *Understanding Tissue-Specific Gene Regulation.* Cell Rep, 2017. **21**(4): p. 1077-1088.
8. Tylee, D.S., D.M. Kawaguchi, and S.J. Glatt, *On the outside, looking in: a review and evaluation of the comparability of blood and brain "-omes".* Am J Med Genet B Neuropsychiatr Genet, 2013. **162B**(7): p. 595-603.

9. Hess, J.L., et al., *Transcriptome-wide mega-analyses reveal joint dysregulation of immunologic genes and transcription regulators in brain and blood in schizophrenia.* Schizophr Res, 2016. **176**(2-3): p. 114-124.
10. Zaman, S., et al., *A Search for Blood Biomarkers for Autism: Peptoids.* Sci Rep, 2016. **6**: p. 19164.
11. Leuzy, A., et al., *Blood-based biomarkers for Alzheimer's disease.* EMBO Mol Med, 2022. **14**(1): p. e14408.
12. Chahine, L.M., M.B. Stern, and A. Chen-Plotkin, *Blood-based biomarkers for Parkinson's disease.* Parkinsonism Relat Disord, 2014. **20 Suppl 1**(0 1): p. S99-103.
13. Basu, M., et al., *Predicting tissue-specific gene expression from whole blood transcriptome.* Sci Adv, 2021. **7**(14).
14. Xu, W., et al., *Blood-based multi-tissue gene expression inference with Bayesian ridge regression.* Bioinformatics, 2020. **36**(12): p. 3788-3794.
15. Hess, J.L., et al., *BrainGENIE: The Brain Gene Expression and Network Imputation Engine.* Transl Psychiatry, 2023. **13**(1): p. 98.
16. Consortium, G.T., *The GTEx Consortium atlas of genetic regulatory effects across human tissues.* Science, 2020. **369**(6509): p. 1318-1330.
17. Song, W.M. and B. Zhang, *Multiscale Embedded Gene Co-expression Network Analysis.* PLoS Comput Biol, 2015. **11**(11): p. e1004574.
18. Robinson, M.D., D.J. McCarthy, and G.K. Smyth, *edgeR: a Bioconductor package for differential expression analysis of digital gene expression data.* Bioinformatics, 2010. **26**(1): p. 139-40.
19. Kuleshov, M.V., et al., *Enrichr: a comprehensive gene set enrichment analysis web server 2016 update.* Nucleic Acids Res, 2016. **44**(W1): p. W90-7.
20. Gene Ontology, C., et al., *The Gene Ontology knowledgebase in 2023.* Genetics, 2023. **224**(1).
21. Milacic, M., et al., *The Reactome Pathway Knowledgebase 2024.* Nucleic Acids Res, 2024. **52**(D1): p. D672-D678.
22. Wang, M., et al., *Transformative Network Modeling of Multi-omics Data Reveals Detailed Circuits, Key Regulators, and Potential Therapeutics for Alzheimer's Disease.* Neuron, 2021. **109**(2): p. 257-272 e14.
23. Gadani, S.P., et al., *IL-4 in the brain: a cytokine to remember.* J Immunol, 2012. **189**(9): p. 4213-9.
24. Kiyota, T., et al., *CNS expression of anti-inflammatory cytokine interleukin-4 attenuates Alzheimer's disease-like pathogenesis in APP+PS1 bigenic mice.* FASEB J, 2010. **24**(8): p. 3093-102.
25. Tang, R.H., R.Q. Qi, and H.Y. Liu, *Interleukin-4 affects microglial autophagic flux.* Neural Regen Res, 2019. **14**(9): p. 1594-1602.
26. Pillai, J.A., et al., *TNFRSF1B Gene Variants and Related Soluble TNFR2 Levels Impact Resilience in Alzheimer's Disease.* Front Aging Neurosci, 2021. **13**: p. 638922.
27. Dong, J., et al., *A hominoid-specific signaling axis regulating the tempo of synaptic maturation.* Cell Rep, 2024. **43**(8): p. 114548.

Figure1

Click here to access/download;Figure;Figure1.tiff

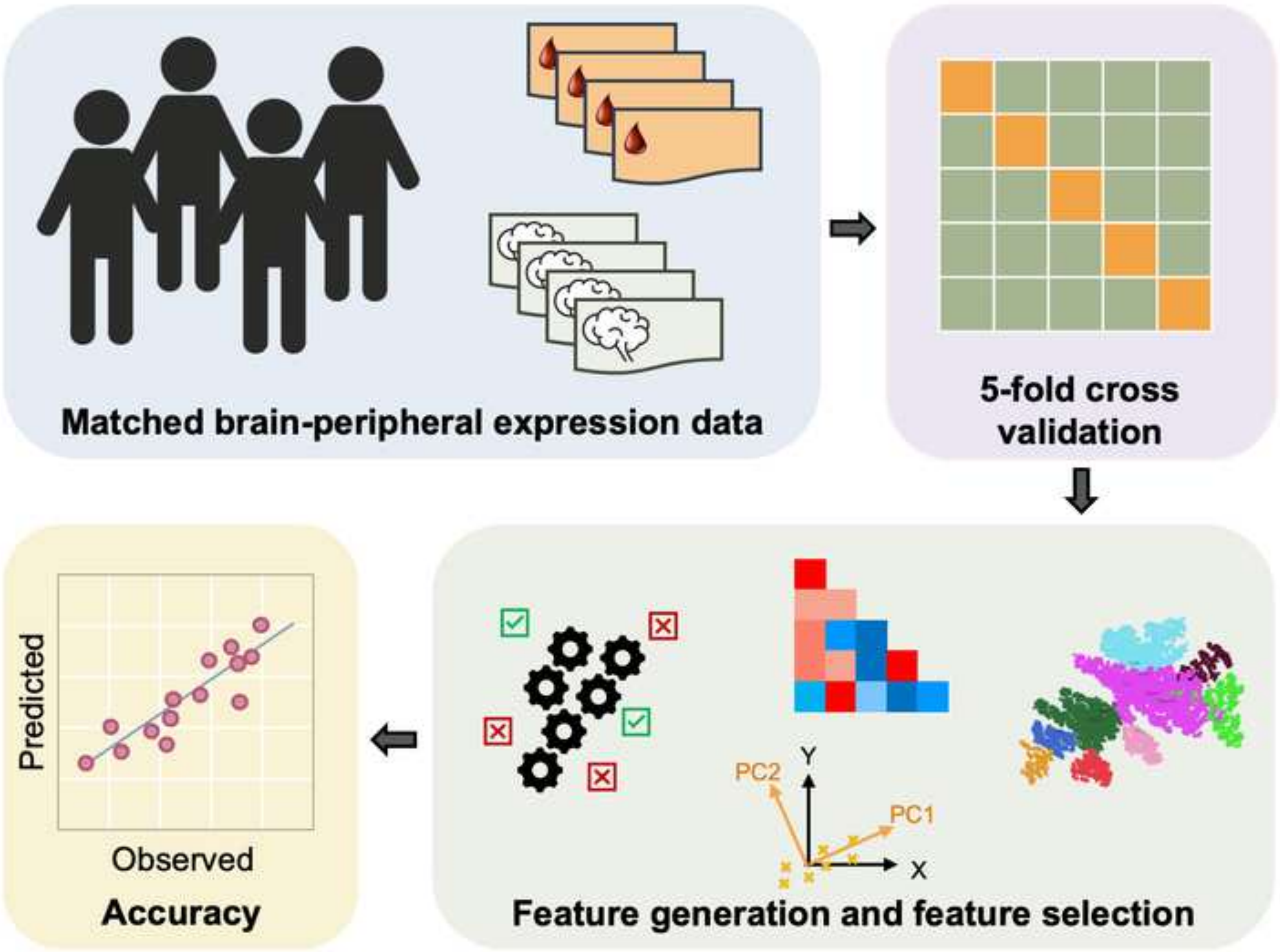

Figure2 Click here to access/download;Figure;Figure2.png

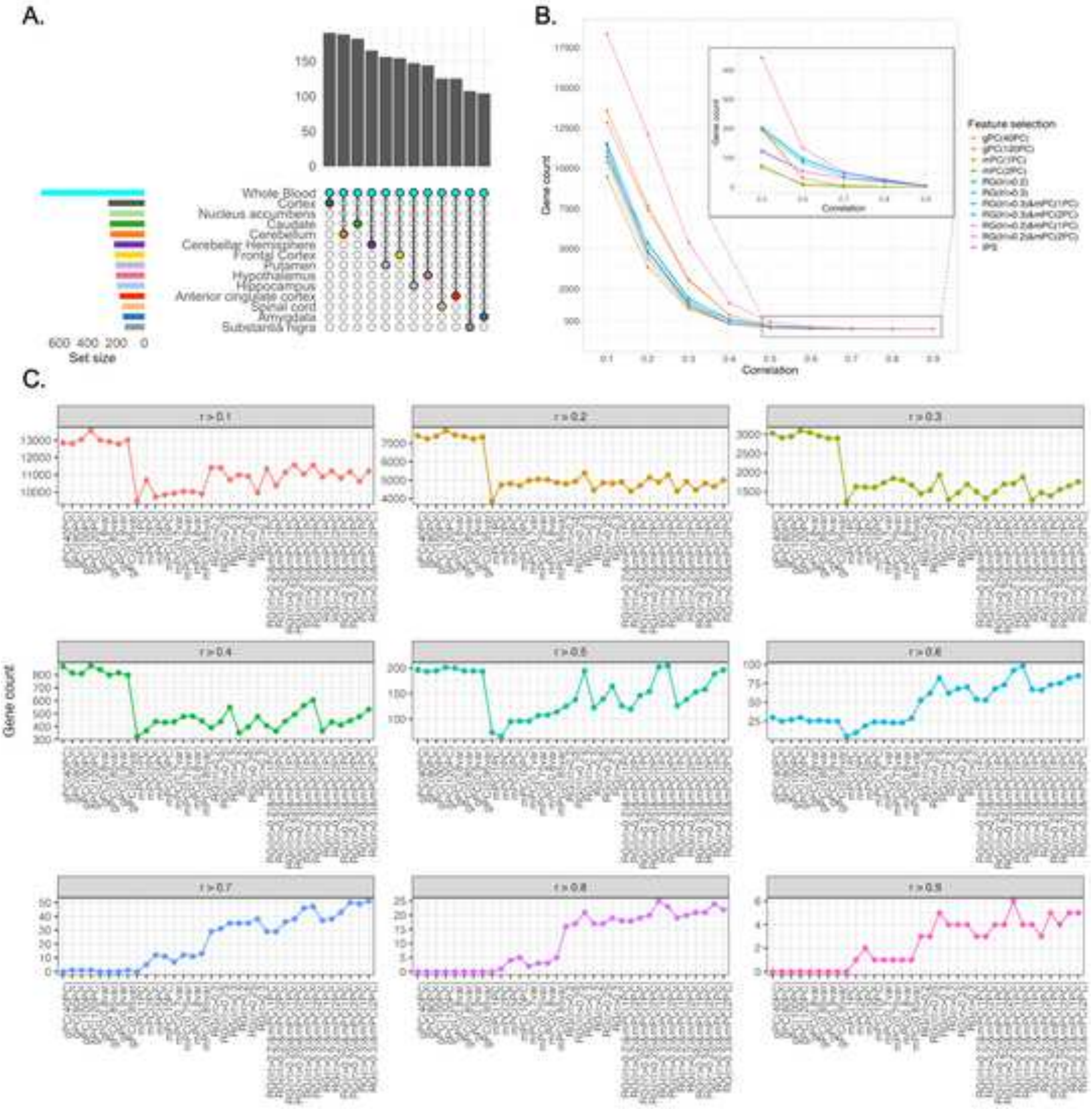

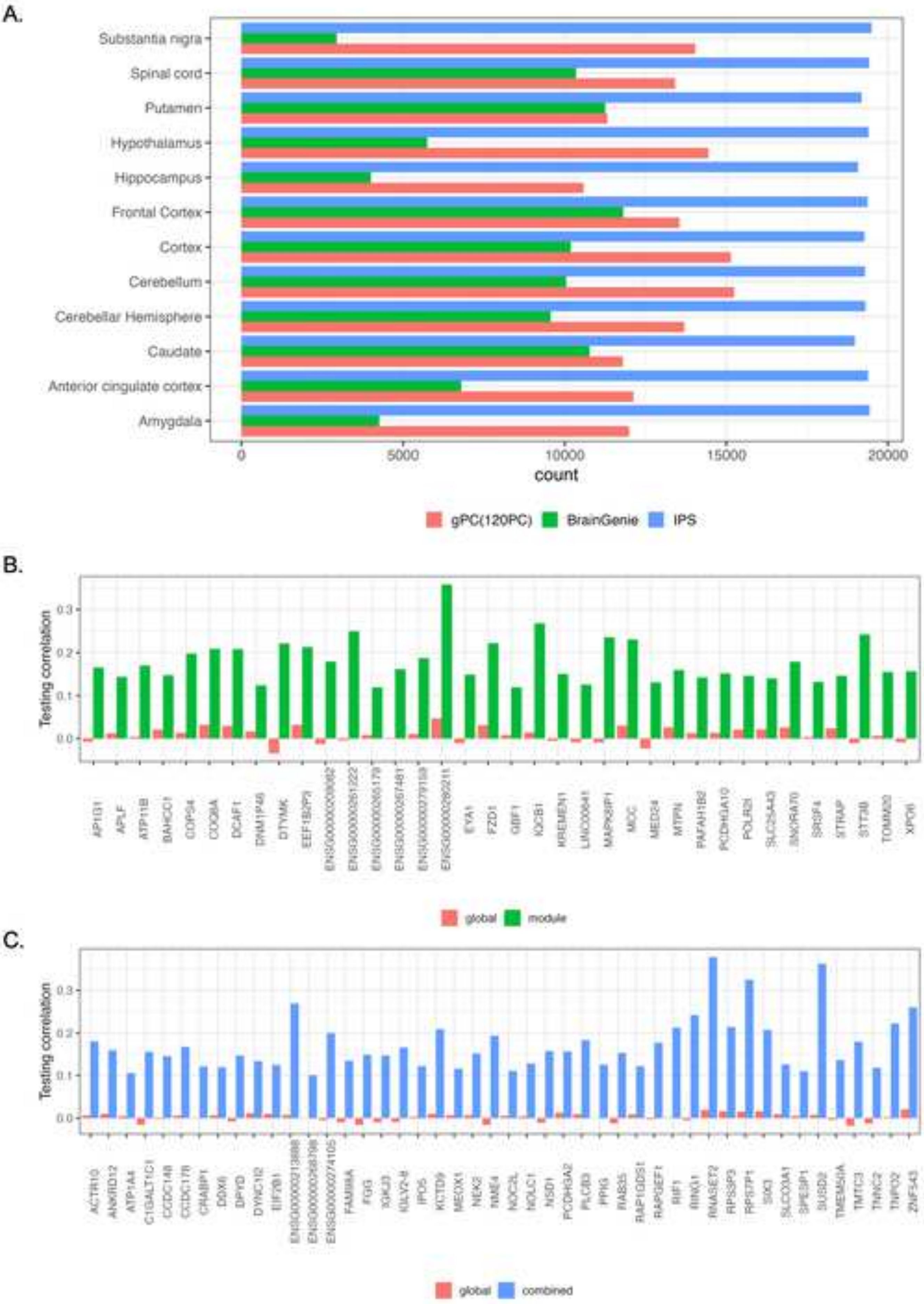
A.
Substantia nigra
Spinal cord
Putamen
Hypothalamus
Hippocampus
Frontal Cortex
Cortex
Cerebellum
Cerebellar Hemisphere
Caudate
Anterior cingulate cortex
Amygdala
0
5000
10000
15000
20000
count
gPC(120PC)
BrainGenie
IPS
B.
Testing correlation
0.0
0.1
0.2
0.3
global
module
C.
Testing correlation
0.0
0.1
0.2
0.3
global
combined

Figure4

Click here to access/download;Figure;Figure4.png

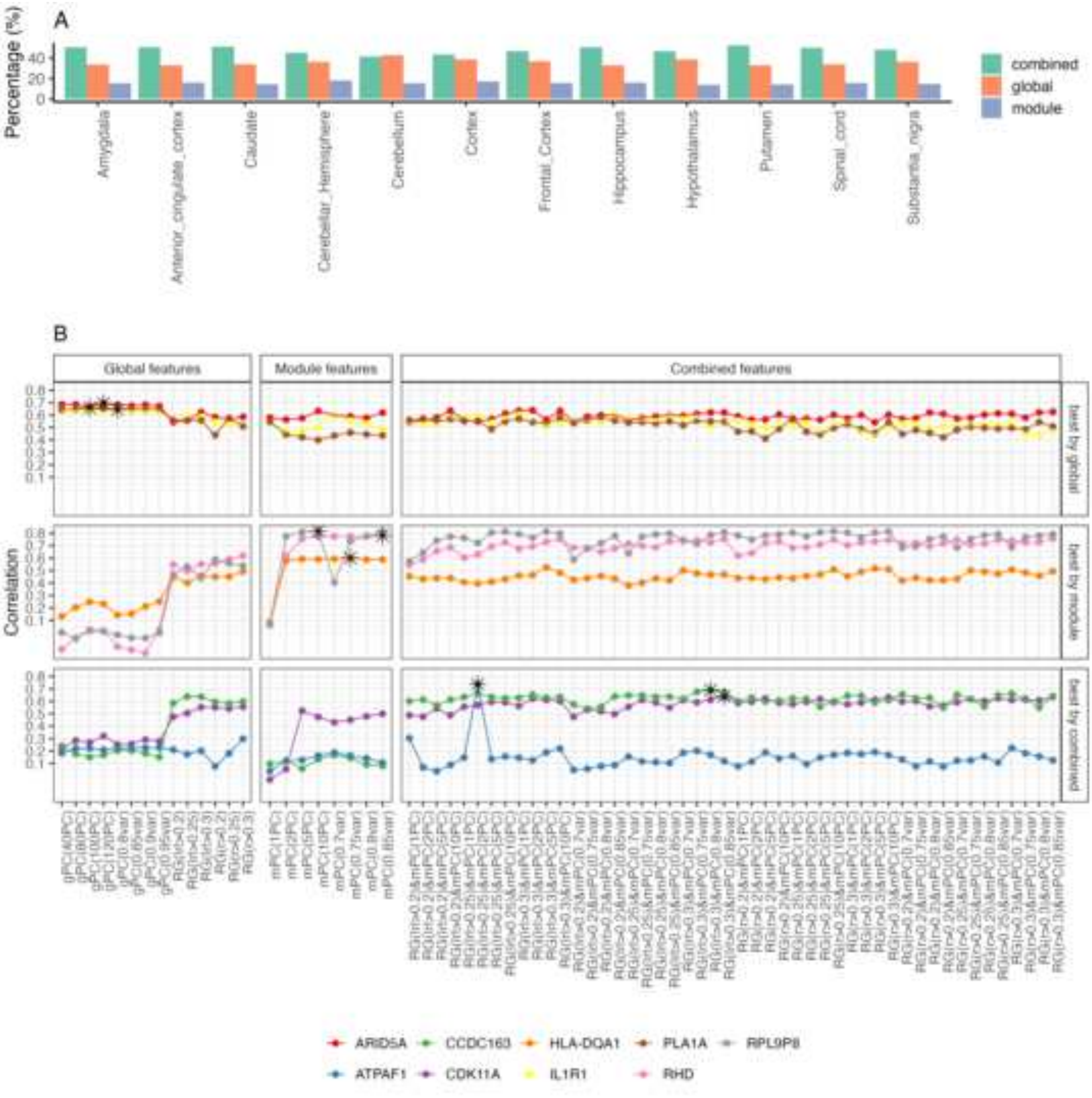

Figure5 Click here to access/download;Figure;Figure5.png

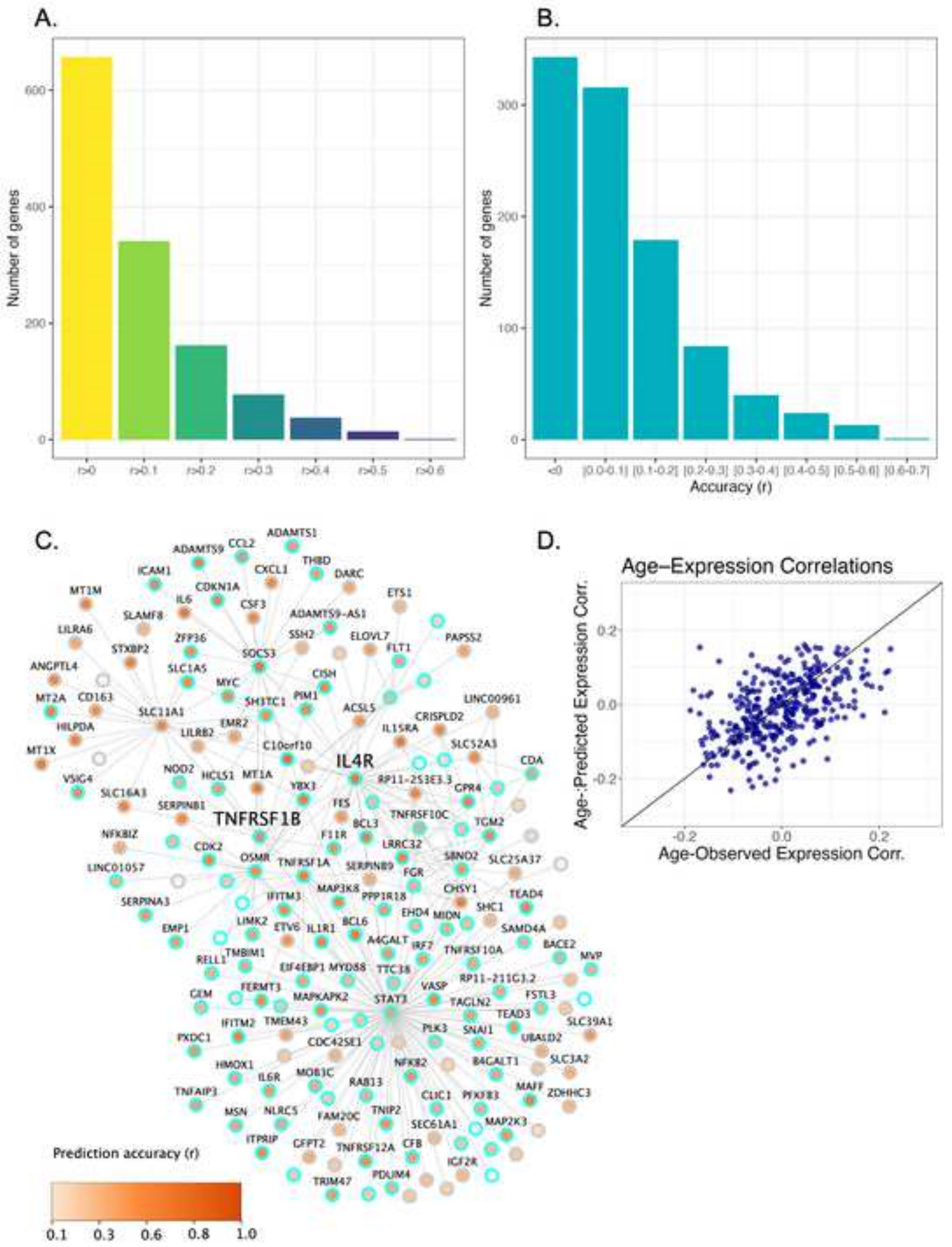

Click here to access/download;Figure;Figure6.png

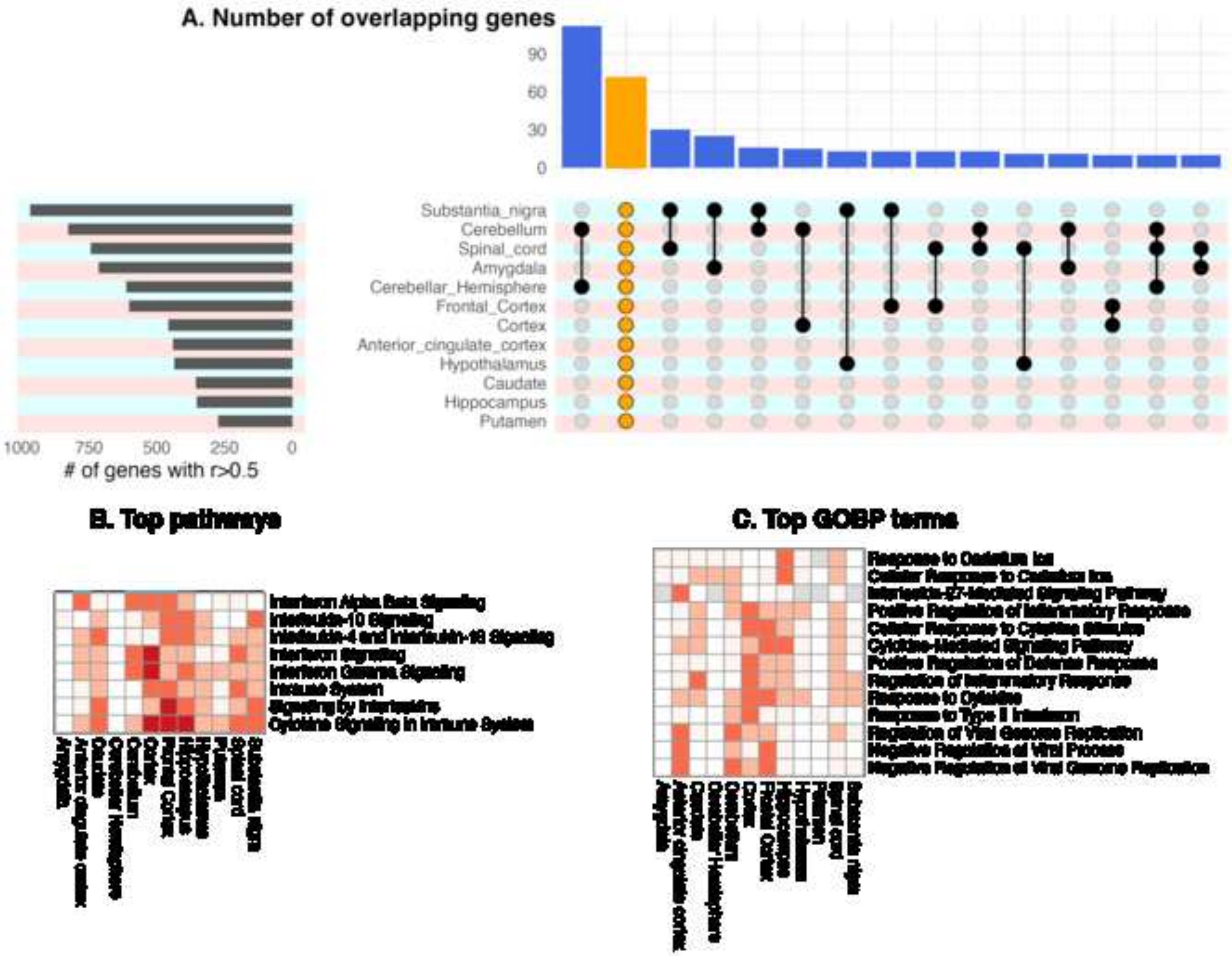

Figure7

Click here to access/download;Figure;Figure7.png

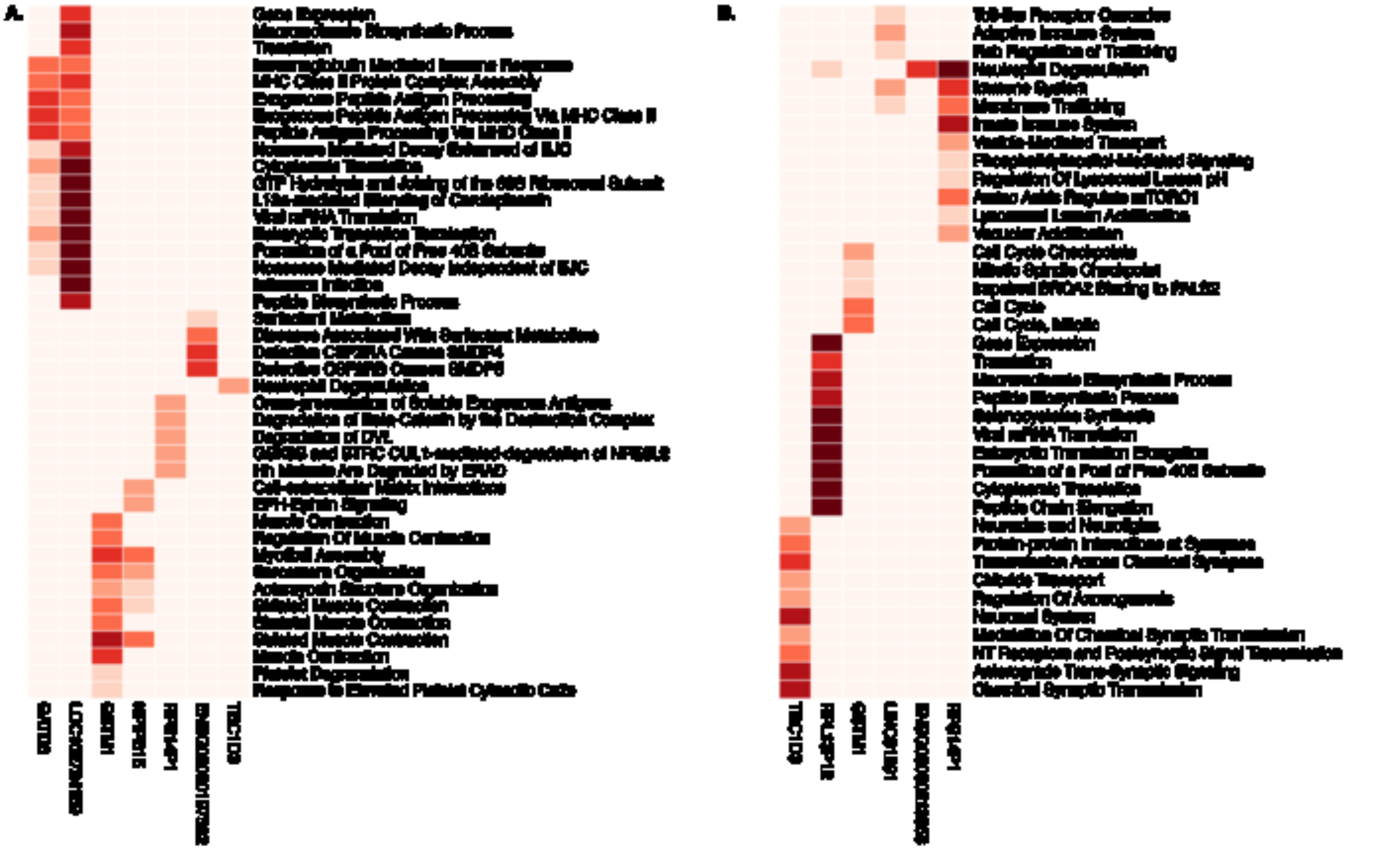

Supplementary Tables

Click here to access/download
Supplementary Material
SupplTables.xlsx

Supplementary Figures

Click here to access/download
Supplementary Material
SupplFigs.docx

Personal Cover

Click here to access/download;Personal Cover;CoverLetter_Octt15th.docx

Icahn School of Medicine at Mount Sinai

Bin Zhang, PhD
Willard T.C. Johnson Research Professor of Neurogenetics
Professor, Departments of Genetics & Genomic Sciences, Pharmacological Sciences, and Artificial Intelligence and Human Health
Director, Mount Sinai Center for Transformative Disease Modeling
Icahn Institute of Genomics, Icahn School of Medicine at Mount Sinai

1425 Madison Avenue (Icahn 3-43)
Box 1498
New York, NY 10029
Phone: 212-659-1726
Fax: 212-659-5507
Email: bin.zhang@mssm.edu

Oct 16th, 2025

Dear Editors,

We are pleased to submit our manuscript, entitled " **Leveraging Machine Learning and Network Biology to Uncover Blood Biomarkers of Brain Gene Expression** ", for consideration at *GigaScience*.

Understanding gene regulation in the human brain is critical for studying neurological and psychiatric disorders, yet direct access to brain tissue is limited. Our study introduces an integrative prediction system (IPS) that combines machine learning with systems biology to predict brain gene expression from blood transcriptomic data. This represents a conceptual and methodological advance in leveraging accessible peripheral data to study molecular processes in the brain.

Our biologically informed, machine learning-based framework offers a scalable, non-invasive tool that will help advance biomarker discovery for neurodegenerative diseases such as Alzheimer's and Parkinson's. Additionally, it uncovers fundamental systems-level relationships between peripheral and central tissues.

Our work aligns with *GigaScience*'s mission to promote data-driven, open, and reproducible research. We believe this manuscript will be of strong interest to the *GigaScience* readership, particularly in the fields of computational biology, neurogenomics, and systems medicine. This integrative model not only expands the potential of non-invasive transcriptomic monitoring but also provides a scalable tool for biomarker discovery in neurological and psychiatric disorders.

We confirm that this manuscript has not been published elsewhere and that all authors have approved its content.

We appreciate your consideration and welcome the opportunity to contribute to your journal.

Sincerely,

Bin Zhang, PhD
Willard T.C. Johnson Research Professor of Neurogenetics
Professor, Genetics & Genomic Sciences, Pharmacological Sciences, AI & Human Health
Director, Mount Sinai Center for Transformative Disease Modeling
Icahn School of Medicine at Mount Sinai

Cigdem Sevim Bayrak, PhD
Assistant Professor, Genetics & Genomic Sciences
Icahn School of Medicine at Mount Sinai

Graphical Abstract

Click here to access/download;Graphical Abstract;graphical_abstract.tiff

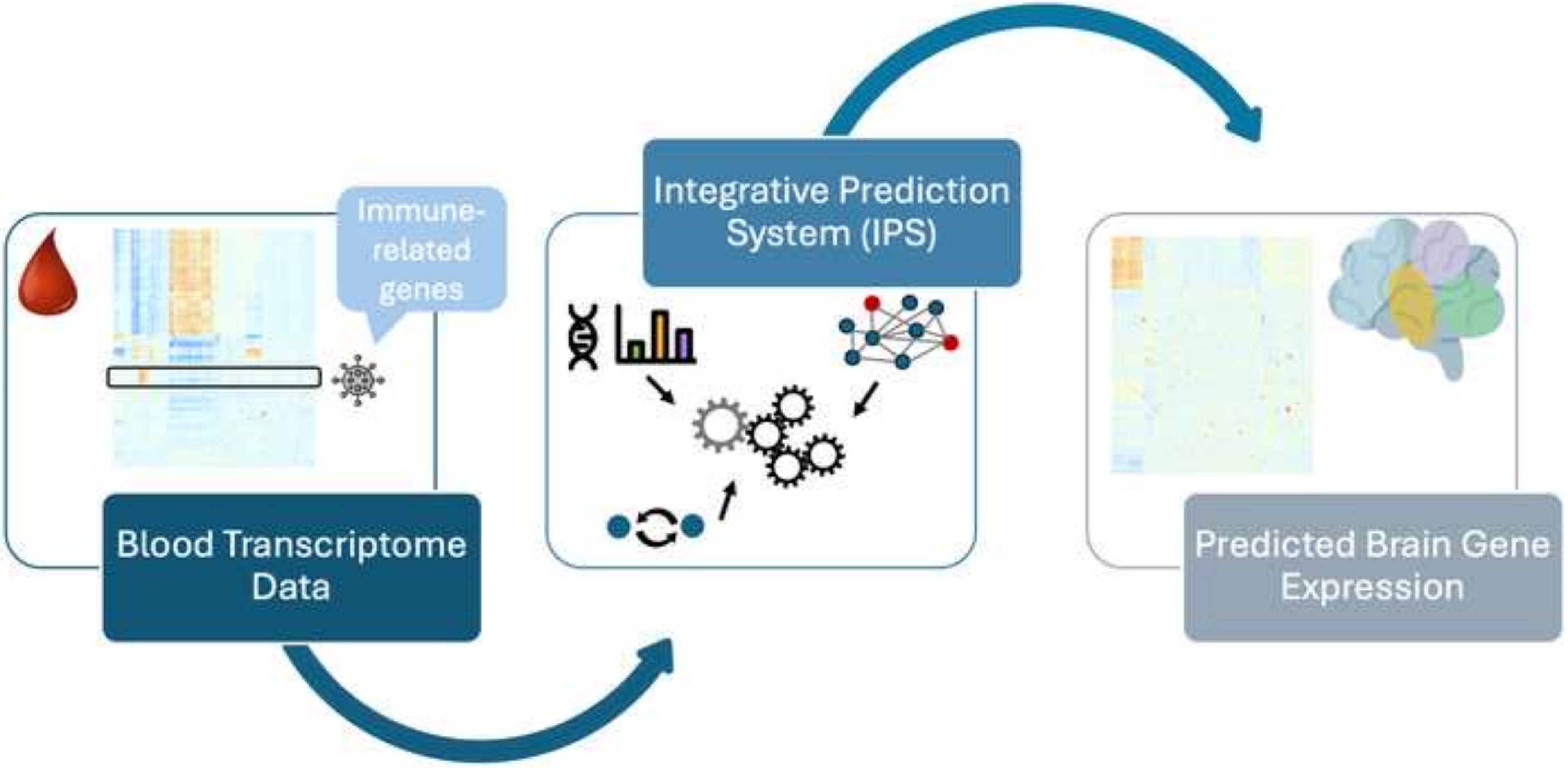

Supplement: giag058_GIGA-D-25-00434_original_submission [file giag058_giga-d-25-00434_original_submission.pdf]
