# Supplementary material for: Leveraging machine learning and network biology approaches to predict brain gene expression from blood transcriptomes
Source: Gigascience. 2026 May 18;15:giag058. doi: 10.1093/gigascience/giag058 (PMC13201078; doi:10.1093/gigascience/giag058)

GigaScience

Leveraging Machine Learning and Network Biology Approaches to Predict Brain Gene Expression from Blood Transcriptomes

--Manuscript Draft--

**Manuscript Number:**	GIGA-D-25-00434R1
**Full Title:**	Leveraging Machine Learning and Network Biology Approaches to Predict Brain Gene Expression from Blood Transcriptomes
**Article Type:**	Research
**Funding Information:**	National Institute on Aging (UH2AG083258) — Prof Bin Zhang<br>National Institute on Aging (R01AG085182) — Prof Bin Zhang<br>National Institute on Aging (RF1AG074010) — Prof Bin Zhang<br>National Institute on Aging (RF1AG054014) — Prof Bin Zhang<br>National Institute on Aging (U01AG046170) — Prof Bin Zhang<br>National Institute on Aging (R01AG068030) — Prof Bin Zhang
**Abstract:**	Blood-based biomarkers offer a promising non-invasive strategy for detecting disease-related changes and monitoring tissue and organ health, including brain function. While recent studies have leveraged blood transcriptomic data to predict gene expression in the brain, existing models generally suffer from poor accuracy, limiting their translational utility. Here, we present an integrative prediction system (IPS) that combines machine learning with network biology to predict region-specific brain gene expression from blood transcriptomic data. Our framework integrates global blood transcriptomic signals, co-expression network features, and inter-tissue gene–gene interaction data linking blood genes to their target genes in the brain. Applied to the Genotype-Tissue Expression (GTEx) cohort, IPS substantially outperforms existing approaches in both the number and accuracy of brain genes that can be reliably predicted from blood. Notably, immune-related blood genes emerged as key contributors to model performance, underscoring the systematic interplay between peripheral immune signaling and central nervous system. These findings highlight the potential of blood-based transcriptomic models as scalable, non-invasive tools for studying brain function and developing diagnostic and prognostic biomarkers for neurological and psychiatric disorders.
**Corresponding Author:**	Cigdem Sevim Bayrak<br>Icahn School of Medicine at Mount Sinai<br>New York, UNITED STATES
**Corresponding Author Secondary Information:**	
**Corresponding Author's Institution:**	Icahn School of Medicine at Mount Sinai
**Corresponding Author's Secondary Institution:**	
**First Author:**	Cigdem Sevim Bayrak
**First Author Secondary Information:**	
**Order of Authors:**	Cigdem Sevim Bayrak<br>Cigdem Sevim Bayrak<br>Qi Zeng<br>Marjan Ilkov<br>Scott J Russo

Powered by Editorial Manager® and ProduXion Manager® from Aries Systems Corporation

	Minghui Wang
	Bin Zhang
**Order of Authors Secondary Information:**	
**Response to Reviewers:**	Responses to Reviewers’ Comments<br><br>We sincerely appreciate the thoughtful comments from the reviewers, which provided us with valuable insights and opportunities to greatly improve the quality of the manuscript. We have revised the manuscript according to the reviewers’ comments and provided a point-by-point response to each suggestion below.<br><br>Responses to the reviewers’ comments are provided below with our responses given in blue.<br><br>Reviewer #1:<br><br>General comments:<br><br>In Bayrak et al's work, the authors build machine learning models to predict brain gene expression based on the paired blood data leveraging network biology and different feature selection method. Some efforts were made in the work; however, it consists of some claims with exaggeration and not supported by the contents. Substantial improvements would be needed for it to get published.<br><br>We thank the reviewer for their overall assessment of our work and for highlighting concerns regarding potential overstatement of certain claims. We have carefully reviewed the manuscript and revised the text to ensure that our conclusions are fully supported by the presented results. In particular, we have moderated language in the abstract and discussion sections and clarified the scope and limitations of our approach. We believe these revisions address the concerns raised.<br><br>Major Comments:<br><br>1.It seems the authors used a simple linear regression (and its variants) model for this whole framework. Is there a justification for that? Of course, sometimes simple model outperforms any of the fancy modern methods, but at least discussions should be added, or other methods should be evaluated/explored for comparison.<br><br>Response #1: We thank the reviewer for this important and constructive comment. The use of linear regression and elastic net models in our framework was a deliberate choice, guided by both prior literature and our own empirical evaluation.<br><br>First, prior studies on predicting tissue-specific gene expression from high-dimensional genomic features (e.g., BrainGenie, TEEBoT, and Bayesian ridge regression–based frameworks) have predominantly employed linear or regularized linear models. This is largely due to their robustness when sample sizes are limited and predictors are highly correlated.<br><br>Second, we evaluated multiple nonlinear machine learning methods, including random forest, support vector machines, and gradient boosting (XGBoost), using the same training and validation framework. As now shown in Supplementary Table 3, these more complex models did not provide consistent improvement in predictive performance compared to linear regression or elastic net. Notably, for moderate-to-high prediction thresholds (e.g., $r > 0.5$), linear and elastic models achieved comparable or better performance in terms of the number of well-predicted genes. Based on these results, we selected linear models for the main analyses to balance accuracy and interpretability.<br><br>Importantly, we would like to emphasize that the proposed framework is model-agnostic and flexible. While linear models performed well in the current setting, particularly at higher correlation thresholds, the pipeline can readily incorporate alternative modeling approaches depending on the research context. The primary contribution of our work is not tied to a specific modeling choice, but rather to the

Powered by Editorial Manager® and ProduXion Manager® from Aries Systems Corporation

demonstration that gene-specific feature selection and model selection can improve predictive performance for individual genes.

To address the reviewer's concern, we have now included a comparison of these alternative methods in the Supplementary Table 3, and we have expanded the Discussion to explicitly comment on model choice. (Methods, page 7, lines 151-155; Results, page 12, lines 262-268; Discussion, pages 21-22, lines 497-504)

2.How is the generalizability of the models? Other than internal training/validation, is there any external validation on any of the models? On page 11, last paragraph: "To assess the predictive capacity of our models for brain expression of AD-related genes, we evaluated the prediction accuracy of the top 1,000 AD key drivers identified in postmortem parahippocampal gyrus (PHG) samples from the Mount Sinai Brain Bank (MSBB) AD cohort…", but this doesn't seem to be applying the model to an external dataset. If the models are built based on data from GTEx, one would expect the they would not be so informative in regard to disease state such as Alzheimer's disease. If the authors' intention is to evaluate the genes implicated in the disease, the models probably wouldn't perform so well since the GTEx data is essentially the comprehensive baseline for "normal" human gene expression.

Response #2: We thank the reviewer for raising this important point regarding model generalizability and external validation. We agree that independent external validation is the gold standard for assessing generalizability. However, at present, there are no publicly available datasets with matched blood and brain expression data in independent cohorts, which precludes direct external validation of our framework.

The analysis involving AD–related genes was not intended as an external validation of the models across datasets or disease states. Rather, it was designed to assess whether genes previously implicated in AD (which were previously identified independently in the MSBB cohort) are among those that can be reliably predicted by our models trained on GTEx data.

Consistent with the reviewer's observation, our models are trained on GTEx samples representing largely non-diseased baseline expression, and we do not expect them to capture disease-specific expression changes. Instead, the AD-related gene analysis evaluates the baseline predictability of genes with known relevance to AD.

We have revised the manuscript to clarify this distinction. We also now emphasize the lack of external matched datasets as a limitation. (Results, page 15, lines 337-340, Discussion, page 22, lines 509-510)

3.How is age modeled in the whole framework? The authors claim that sex had been regressed out in the dataset prior to model, and on page 13 there is a section "Preservation of Age-Associated Expression Patterns in Predicted Gene Profiles", does it indicate that age is just left out in the models? In the section, they state that "We first identified highly predictable genes (CV r > 0.5)" (how many are there? It would be helpful to be specific) and computed the correlation before/after prediction. If the genes are highly correlated in the first place, r1/r2 would be intuitively correlated as well. I would argue many of the age-related changes are actually present within those genes the models didn't predict well, which contradict the conclusion "the model effectively preserves age-associated expression patterns". It would be helpful to compare the genes the model predicted well and poorly to see if age plays a role, or, to include age in the model in the first place to see if it makes any impact.

Response #3: We thank the reviewer for the thoughtful and detailed comments regarding age modeling and the interpretation of the age-associated expression analysis. We clarify that age was not regressed out prior to model training; only technical covariates and sex were adjusted for, as described in the Methods, subsection "Dataset".

The analysis in the section "Preservation of Age-Associated Expression Patterns in Predicted Gene Profiles" was intended as a post hoc evaluation of whether age-related transcriptional signals present in the observed brain expression are retained in the predicted expression profiles, rather than as a test of whether the models explicitly

Powered by Editorial Manager® and ProduXion Manager® from Aries Systems Corporation

model age effects.

In the hippocampus, 353 genes met the criterion of high predictability (CV r > 0.5). For these genes, we computed the correlation between observed expression and age (r_1) and compared it to the correlation between predicted expression and age (r_2). This correspondence was moderate (r = 0.496), indicating that age-associated expression patterns are partially preserved for well-predicted genes.
We agree with the reviewer that preservation of age-associated effects should not be assumed for all genes. To address this, we extended the analysis by stratifying genes based on prediction accuracy. Among poorly predicted genes (CV r<0.1; N=536 genes), the correspondence between r_1 and r_2 was substantially weaker (r = 0.195). This comparison demonstrates that preservation of age-associated expression patterns depends on prediction quality and is not a trivial consequence of correlation structure alone.

We have revised the manuscript to explicitly report the number of genes in each group and to refine the interpretation of the results. The text now emphasizes that predicted expression preserves age-associated patterns for a subset of well-predicted genes, rather than across the entire transcriptome. We included the stratified analysis in Supplementary Table S7. (Results, page 17, lines 376-391)

4.In general, blood-brain cross-tissue gene expression prediction is weak, and the current models do not demonstrate strong or reliable performance. Therefore, the statement in the last paragraph on page 16 regarding the feasibility of using blood-based transcriptomic models as diagnostic or prognostic tools, substantially overstates what the current results support. At present, the models lack external validation and their performance in actual disease cohorts is unknown. The ROSMAP dataset, which contains paired blood and brain RNA-seq (albeit with some time lag), could serve as a valuable resource for assessing generalizability and helping determine whether these models can meaningfully predict brain expression in independent samples.

Response #4: We thank the reviewer for suggesting the use of the ROSMAP dataset to assess generalizability. In response, we applied the same prediction pipeline to ROSMAP using monocyte RNA-seq as predictors and dorsolateral prefrontal cortex expression as the outcome. While this does not constitute a formal external validation due to differences in cell type, cohort composition, and sample collection timing, it provides a useful comparison across datasets. We observed that fewer genes were predictable in ROSMAP than in GTEx across all thresholds; however, the overlap of top predicted genes was significantly greater than expected by chance. These results are presented in the newly added Supplementary Figure S8. (Methods, page 9, lines 183-187, Results, pages 20-21 lines 460-482)

We agree that external validation in an appropriately matched cohort would be valuable; however, at present, no such dataset is available. Accordingly, we have revised the Discussion to temper the claims regarding diagnostic or prognostic applications, emphasizing that these models are currently best suited for research use and that their performance in clinical cohorts remains to be established. (Discussion, page 22, lines 519-528)

5. How are the models transferable between different brain regions? There are some statistics on the percentage of genes for which each feature set performed best across different brain regions (Figure 4), have the authors tried to assess how one model trained from one region to predict related regions?

Response #5: We thank the reviewer for the suggestion regarding cross-region model transfer. In our framework, each gene in each brain region is modeled separately using blood gene expression as input. As a result, each model captures the blood-to-brain relationships and regulatory structure that are specific to that tissue.

While it is theoretically possible to apply a model trained in one brain region to predict expression in another, we anticipate performance would be limited due to region-specific differences in gene regulation, co-expression networks, and blood–brain correlations. Therefore, we focus on optimizing predictions for each tissue separately. We have clarified this point in the manuscript to emphasize that each model is intended

Powered by Editorial Manager® and ProduXion Manager® from Aries Systems Corporation

for the specific brain region in which it was trained. (Results, page 15, lines 329-332)

Minor Comments:

1.The workflow chart (Figure 1) is too abstract. It would benefit the readers to have a more concrete description of the actual work done in the manuscript. e.g. the specific feature selection methods explored, how final models are picked for evaluation, what kind of evaluations have been done etc. More texts in the picture would help.

Response #6: We thank the reviewer for this helpful suggestion. In the revised manuscript, we have substantially updated Figure 1 to provide a more concrete and detailed representation of the workflow. We believe the revised workflow now provides a clearer and more informative overview of the design and analysis pipeline.

2.BrainGENIE will need a citation and some introductions in the introduction section since it has been used in the manuscript for comparison.

Response #7: We thank the reviewer for the suggestion. We have revised the Introduction to provide a clearer description of BrainGENIE. (page 4, lines 63-67)

3. In the title "to Uncover Blood Biomarkers" suggests that the goal of the work is for feature section. However, the contents of the manuscript are more focused on model building and evaluation based on different feature section method, since at least two methods are based purely based on correlation. I would revise the title to reflect the facts.

Response 8: We thank the reviewer for this note. While the term “biomarker” in our current title refers to blood gene expression features that can predict brain expression, we agree that the manuscript primarily focuses on building and evaluating predictive models rather than validating biomarkers in clinical cohorts. To more accurately reflect the scope of the work, we propose revising the title to:
“Leveraging Machine Learning and Network Biology Approaches to Predict Brain Gene Expression from Blood Transcriptomes”

4. There is no legend for the heatmaps in Figure 6 & 7.

Response #9: We thank the reviewer for this comment. We have revised Figures 6 and 7 to include labeled legends for each heatmap.

Reviewer #2:

General comments:

The manuscript presents an integrative framework that combines machine learning and network biology to predict brain gene expression from blood transcriptomic data. The topic is important. However, several issues related to clarity, methodological description, and result presentation need to be addressed to improve the rigor and reproducibility of the study.

We thank the reviewer for recognizing the importance of our work and for the constructive feedback. In response, we have carefully revised the manuscript to improve the clarity of the methodology, providing additional details where necessary to facilitate reproducibility. We have also revised the presentation of results to improve readability and ensure that the key findings are clearly conveyed. We believe these revisions address the concerns raised and strengthen the overall quality of the manuscript.

Major Comments:

1.It lacks sufficient clarity in the description of the prediction models. The Methods section states that linear regression and elastic net models were used, but the exact formulation of the linear model is not clearly described. It is unclear:
What is the dependent variable and what features are included in the regression?
An explicit mathematical formulation of the regression model (e.g., an equation) should

be provided to clearly illustrate how the features enter the model and how predictions are generated.

Response #10: We thank the reviewer for highlighting the need for clarity in model description. For each brain gene, we trained linear regression and elastic net models using normalized blood gene expression as features. The dependent variable is the residualized expression of a single brain gene in a specific brain region, after regressing out sex and sequencing-related covariates. Linear regression models predict brain expression as a weighted sum of blood gene expression, while elastic net models include L1/L2 penalties to stabilize coefficient estimates in the presence of highly correlated predictors.

Predictions were generated for training and test sets, and performance was evaluated using Pearson correlation between predicted and observed expression. An explicit mathematical formulation of both models has been added to the Methods section, and all code is available via GitLab. We believe this revision fully clarifies the modeling framework for readers. (Methods, page 8, lines 164-178)

2.While the manuscript reports that IPS outperforms BrainGENIE, the comparison focuses primarily on the number of predicted genes. It would be more informative to also: Show the overall distribution of average cross-validation correlation coefficients (CV r) for IPS versus BrainGENIE. Provide a direct comparison of prediction accuracy distributions rather than only gene counts above a threshold.

Response #11: We thank the reviewer for this helpful suggestion. In response, we have now examined the full distribution of average cross-validation correlation coefficients (CV r) for both IPS and BrainGENIE. As background, BrainGENIE uses the top 40 PCs with a linear modeling framework to predict all genes, which is one of the approaches we already include in our integrated framework. To ensure a fair and controlled comparison, rather than rerunning BrainGENIE, we compared IPS directly to a linear model trained on the top 40 PCs across all genes using identical training/testing splits. Specifically, we generated density plots showing the overall distribution of prediction accuracies across all genes and performed a direct comparison of the two methods' CV r distributions. These results are now presented in newly added Figure 3B. The figure demonstrates that IPS shows a consistently shifted distribution toward higher CV r values relative to BrainGENIE, supporting the conclusion that IPS achieves improved prediction performance beyond differences in gene counts above threshold. (Results, page 11, lines 237-245)

3.The manuscript compares IPS mainly with BrainGENIE. However, previous methods such as TEEBoT (ref. 13) and Bayesian ridge regression-based approaches (ref. 14) are cited but not systematically benchmarked. A more comprehensive comparison with these methods would strengthen the claims of improved performance.

Response #12: We thank the reviewer for this important suggestion. We agree that benchmarking against previously published approaches strengthens the evaluation of IPS.
TEEBoT and B-GEX were both trained on earlier versions of GTEx that included substantially fewer samples than the version used in our study. Because these methods were trained on earlier releases, direct retraining under identical conditions was not feasible.
Available TEEBoT results were limited to two brain regions (caudate and cerebellum), and B-GEX results were available only for cerebellum. To enable comparison, we evaluated the number of genes predicted at increasing correlation thresholds ($r = 0.0–0.9$) and compared these distributions to TEEBoT. For cerebellum, B-GEX reported a mean gene-level correlation of 0.044, which was lower than that achieved by our integrated IPS approach. Across available regions and thresholds, IPS consistently predicted a greater number of genes at comparable or higher accuracy levels than both TEEBoT and B-GEX.

We have now incorporated this comparison and its limitations into the Results section. (Results, pages 11-12, pages 246-261).

4. It remains unclear whether prediction accuracy is influenced by gene expression

Powered by Editorial Manager® and ProduXion Manager® from Aries Systems Corporation

	abundance in blood or brain. Specifically: Are highly expressed genes in blood generally better predicted? Is there a correlation between gene expression level and prediction performance? An explicit analysis addressing this potential confounding factor would improve the biological interpretation of the results.<br><br>Response #13: We thank the reviewer for raising this important point. To evaluate whether prediction performance is confounded by gene expression abundance, we explicitly examined the relationship between gene expression level and prediction accuracy. For each brain region, we computed the correlation between gene expression abundance and prediction accuracy. This analysis was performed separately using (i) gene expression levels in blood (Supp Figure S6) and (ii) gene expression levels in brain tissue (Supp Figure S7). Across all regions, the correlation between abundance and prediction accuracy was negligible ($\|r\| < 0.1$). This indicates that prediction performance is not systematically driven by highly expressed genes. The top-performing genes showed that they exhibit moderate expression levels in both blood and brain tissues. We have included this new analysis in the Results section. (Results, page 19, lines 450-459).<br><br>Minor:<br><br>1.While Figure 2A shows the number of paired blood-brain samples across tissues, the manuscript does not clearly state: The exact number of samples used for each brain region in model training. Whether the same individuals are used across different brain regions. A comprehensive summary table describing sample size, tissue type, preservation method (e.g., PAXgene vs. frozen), and relevant covariates should be provided as a supplementary table.<br><br>Response #14: We thank the reviewer for this suggestion. We have added a comprehensive summary table (Supplementary Table S1) showing, for each brain tissue, the preservation method, the number of individuals with paired whole blood samples, mean age, and sex distribution).<br><br>2. Figure 2A does not include a clear y-axis label. The metric being displayed (e.g., number of samples or individuals) should be explicitly labeled to avoid ambiguity.<br><br>Response #15: We thank the reviewer for this comment. We have revised Figure 2A to include clear axis labels.<br><br>3. Figure S1 presents prediction performance across different feature combinations, but the details of these combinations are not adequately explained in the main text or figure legend. The authors should clearly describe: What each feature combination represents.<br><br>Response #16: We thank the reviewer for this suggestion. To clarify the feature combinations used in the prediction analysis, we have now included a comprehensive supplementary table (Supplementary Table S6) that lists, for each correlation cutoff and brain region, the number of genes predicted using 1–5 top methods combined. This table also specifies the combined top methods, allowing readers to see exactly which features were included in each set. (Results, page 14, lines 310-328)<br><br>4. Figures 6B and 6C present pathway and GO enrichment heatmaps, but no color scale is shown. A color bar indicating the meaning of the colors (e.g., -log10 P value or adjusted P value) should be added.<br><br>Response #17: We thank the reviewer for this comment. We have revised Figures 6 and 7 to include labeled legends for each heatmap. The heatmaps are plotted using -log10(P-value). We have also edited the legends for clarity.
**Additional Information:**	
**Question**	**Response**
Are you submitting this manuscript to a special series or article collection?	No

**Experimental design and statistics**<br><br>Full details of the experimental design and statistical methods used should be given in the Methods section, as detailed in our Minimum Standards Reporting Checklist. Information essential to interpreting the data presented should be made available in the figure legends.<br><br>Have you included all the information requested in your manuscript?	Yes
**Resources**<br><br>A description of all resources used, including antibodies, cell lines, animals and software tools, with enough information to allow them to be uniquely identified, should be included in the Methods section. Authors are strongly encouraged to cite Research Resource Identifiers (RRIDs) for antibodies, model organisms and tools, where possible.<br><br>Have you included the information requested as detailed in our Minimum Standards Reporting Checklist?	Yes
**Availability of data and materials**<br><br>All datasets and code on which the conclusions of the paper rely must be either included in your submission or deposited in publicly available repositories (where available and ethically appropriate), referencing such data using a unique identifier in the references and in the "Availability of Data and Materials" section of your manuscript.<br><br>Have you have met the above requirement as detailed in our Minimum Standards Reporting Checklist?	Yes

GigaScience has policies and guidelines in place for the use of generative AI-writing tools such as ChatGPT. If you have used such writing tools to assist with writing the manuscript this must be declared and cited in the text. Authors should not list AI-writing tools and other AI-assisted technologies as an author or co-author and should acknowledge that they are fully responsible for text generated or refined by AI-writing tools.<p><br><br>A summary of use (particularly in the introduction or among methods) needs to be included at the end of the paper, and the outputs should also be included as a supplementary file hosted in GigaDB or other open repositories. Please read our guidelines for more information. <p><br><br>By submitting to GigaScience, you are aware of the journal's AI-writing tools policy, and if you have declared use of such tools below, you have acknowledged this where appropriate in your manuscript and have made a summary of use and outputs available. <p><br><b>AI-assisted writing tools have been used in the preparation of this manuscript?	No

Powered by Editorial Manager® and ProduXion Manager® from Aries Systems Corporation

Manuscript
Click here to access/download;Manuscript;Maintext_05-12-26addDOI.docx

Leveraging Machine Learning and Network Biology Approaches to Predict Brain Gene Expression from Blood Transcriptomes

Cigdem Sevim Bayrak[1,2], Qi Zeng[1,2], Marjan Ilkov[1,2], Scott J Russo[3,4,5], Minghui Wang[1,2], Bin Zhang[1,2]

[1] Department of Genetics and Genomic Sciences, Icahn School of Medicine at Mt Sinai, New York, NY, USA

[2] Mount Sinai Center for Transformative Disease Modeling, Icahn School of Medicine at Mount Sinai, New York, NY, USA

[3] Friedman Brain Institute, Icahn School of Medicine at Mount Sinai, New York, NY, USA

[4] Nash Family Department of Neuroscience, Icahn School of Medicine at Mount Sinai, New York, NY, USA

[5] Brain and Body Research Institute, Icahn School of Medicine at Mount Sinai, New York, NY, USA

Cigdem Sevim Bayrak [0000-0002-3883-5535]; Qi Zeng [0009-0003-2226-5122]; Marjan Ilkov [0000-0002-1464-9252]; Scott Russo [0000-0002-6470-1805]; Minghui Wang [0000-0001-9171-4962]; Bin Zhang [0000-0002-9549-5653]

Abstract

Blood-based biomarkers offer a promising non-invasive strategy for detecting disease-related changes and monitoring tissue and organ health, including brain function. While recent studies have leveraged blood transcriptomic data to predict gene expression in the brain, existing models generally suffer from poor accuracy, limiting their translational utility. Here, we present an integrative prediction system (IPS) that combines machine learning with network biology to predict region-specific brain gene expression from blood transcriptomic data. Our framework integrates global blood transcriptomic signals, co-expression network features, and inter-tissue gene–gene interaction data linking blood genes to their target genes in the brain. Applied to the Genotype-Tissue Expression (GTEx) cohort, IPS substantially outperforms existing approaches in both the number and accuracy of brain genes that can be reliably predicted from blood. Notably, immune-related blood genes emerged as key contributors to model performance, underscoring the systematic interplay between peripheral immune signaling and central nervous system. These findings highlight the potential of blood-based transcriptomic models as scalable, non-invasive tools for studying brain function and developing diagnostic and prognostic biomarkers for neurological and psychiatric disorders.

Introduction

Blood biomarkers are emerging as a minimally invasive approach to investigate both neurodegenerative disease pathology and normal brain function. Neurodegenerative diseases (NDDs), including Alzheimer's disease (AD) and Parkinson's disease (PD), affect millions globally and account for approximately 15% of the population. With the global population aging, the burden of these disorders is expected to rise significantly [1]. Early and precise diagnosis is essential for effective prevention and treatment but remains challenging in clinical settings.

While neuroimaging and cerebrospinal fluid (CSF) biomarkers have advanced in vivo characterization of disease processes, their use is limited by cost, limited accessibility, and invasiveness[2]. Blood-based biomarkers offer a minimally invasive, cost-effective alternative for detecting disease-related changes and monitoring brain health across the lifespan[3], with the potential to transform both clinical practice and research into normal and pathological brain aging.

The brain, the most complex organ in the human body, consists of billions of neurons forming trillions of connections with each other[4]. Characterizing the brain transcriptome is essential for understanding the molecular mechanisms that underpin neurological disorders. However, the limited availability of human brain tissue samples presents a significant challenge[5]. While gene expression patterns in the brain are largely consistent across individuals, distinct transcriptional profiles are observed across different tissues, with tissue-specific characteristics primarily determined by a select group of genes[6, 7]. Notably, studies have shown a strong correlation between gene expression profiles in the brain and blood[8], with co-expression networks of genes maintained across both tissues[9]. This strong correlation, along with the ease of access and cost-effectiveness of blood samples, makes the blood transcriptome a valuable resource for studying neurological disorders. Hence, many research efforts focus on utilizing patient blood samples to identify gene signatures associated with these conditions[10-12]. Predicting brain gene expression using blood transcriptome data could significantly enhance our understanding of brain-specific gene activity and disease-related changes. Moreover, incorporating transcriptional profiles from various tissues could further improve the accuracy of predictions regarding gene expression in the brain.

Recent studies have leveraged blood transcriptome data to develop generalized, transcriptome-wide models for predicting brain expression data[13-15]. Among these, BrainGENIE is a computational framework that uses peripheral blood gene expression profiles to impute brain tissue-specific expression levels across multiple brain regions. BrainGENIE employs principal component analysis (PCA) for feature selection to address the high dimensionality of the data, and applies regression-based modeling to predict brain expression patterns. While such approaches have demonstrated improved performance over genotype-based prediction models for subsets of genes, their overall predictive accuracy remains limited. More advanced and targeted approaches are required for more accurately capturing the complex, tissue-specific interactions between peripheral and brain gene expression. To improve gene-specific prediction performance, we propose an Integrative Prediction System (IPS), which leverages a diverse set of feature selection algorithms to model the complex, tissue-specific relationships, rather than relying on a singular, genome-wide predictive framework. In this study, we apply both (i) unsupervised feature selection, where features are selected without reference to the target brain gene, and (ii) supervised feature selection, where features are selected based on their relationship with the target brain gene. Leveraging paired blood and brain expression data from the GTEx dataset (v.8)[16], this study seeks to enhance the accuracy of gene-specific predictions across twelve brain tissues by integrating features from both blood transcriptomic data and co-expression network models derived from the MEGENA network[17]. Our comparison of different feature sets and selection strategies demonstrates that different approaches predict distinct gene sets, highlighting the importance of using complementary methods to improve gene-specific predictions. This approach lays the groundwork for more precise, tissue-specific biomarkers and better understanding of gene expression dynamics across tissues. Prediction

models based on peripheral tissue could extend their applicability beyond brain tissue and NDDs. When applied to other tissues, such as the heart and lungs, these models have the potential to provide valuable diagnostic and prognostic insights for a wide range of conditions.

Materials and Methods

A general workflow of this study is shown in Figure 1, and the details are explained below.

Dataset

We have downloaded the raw count of RNA-seq data from the GTEx (v8) database and normalized using trimmed mean of M-values normalization (TMM) method to adjust for sequencing library size difference[18]. The normalized gene expressions were then log2 transformed. We applied a linear model to adjust for the covariates including “SMCENTER” (collection sites), “SMRIN” (RNA integrity), “SMTSISCH” (ischemic time), “SMEXNCRT” (exonic rate), “SMRRNART” (rRNA rate), “SMNTERRT” (intergenic rate) and “SEX” (gender) and used the residuals from the regression model for downstream analysis. Next, we have prepared paired blood and brain transcriptome data for each brain tissue. Figure 2A shows the number of individuals with paired blood-brain data across different brain tissues. A detailed summary of the corresponding sample characteristics is provided in Supplementary Table S1, including the preservation method, number of individuals with paired whole blood samples, mean age, and sex distribution for each tissue.

Network models

In order to identify groups of closely co-expressed genes, we have generated tissue-specific co-expression networks utilizing the Multiscale Embedded Gene co-Expression Network Analysis (MEGENA)[17] R package. The gene expression matrix was permuted (n = 10) across the

samples to calculate the false-positive rate (FPR) and the corresponding false discovery rate (FDR) for each correlation coefficient cutoff. An FDR threshold of 0.05 was then applied to determine the correlation coefficient cutoff that effectively filtered out insignificant correlations. The significant gene pairs were sorted by their absolute Pearson correlation coefficients. These sorted gene pairs were sequentially examined to determine if they could be placed on a three-dimensional topological sphere without intersecting other edges, a process known as the planarity test. Multiscale clustering analysis (MCA) was applied on the resulting co-expression network, planar filtered network (PFN), to identify network clusters (e.g., gene modules) at various compactness resolutions. MCA divides the parent module into child modules by searching for an optimal partition based on Newman's modularity. Multiscale hub analysis was then performed by to identify nodes with significantly higher network connectivity compared to the randomly permuted planar networks ($P < 0.05$). Finally, PCA was applied on each module to determine module features.

Feature selection

Unsupervised feature selection: To reduce the dimensionality of the blood expression training data, principal component analysis (PCA) was applied using the prcomp() R function. We evaluated different number of principal components (e.g., 40, 80, 100, and 120 PCs) and explored various thresholds for the percentage of explained variance (e.g., 80%, 85%, 90%, and 95%).

Supervised feature selection: To select subsets of features that are highly correlated with the target brain gene expression data, we have applied correlation-based feature selection by Pearson’s correlation measure using the cor() R function. We evaluated highly correlated features

(e.g., $|\rho| > \{0.2, 0.25, \text{ and } 0.3\}$) as well as positively correlated features (e.g.,$\rho > \{0.2, 0.25, \text{ and } 0.3\}$).

Network features selection: To generate network-based features from the blood modules, eigengenes (the first principal component) of each module were calculated. To further explore the effectiveness of network features, we also explored using the top 2, 5, and 10 PCs and the PCs that explained 70%, 75%, 80% and 85% of the total variance of each module.

Training and prediction

To develop gene-specific prediction models, we applied 5-fold cross-validation, where in each fold, 4/5 of the data were used for training and 1/5 served as a hold-out test set. In each training subset, we select features by choosing (i) top PCs from principal component analysis on blood transcriptome data, (ii) highly correlated blood gene markers with target brain genes, (iii) top PCs from each network module, (iv) union of highly correlated blood gene markers and top PCs of blood gene expression network modules. Then, we performed both linear regression, using the lm() function, and elastic net modeling, using the glmnet() function, on the selected features in R (version 4.2.0). In addition to these linear approaches, we evaluated several nonlinear machine learning methods within a unified training framework implemented via the caret package. Specifically, we trained random forest (method = “rf”), support vector machines (method = “svmLinear2”), and gradient boosting models (method = “xgbTree”), with model tuning and evaluation conducted using cross-validation and optimization of the R-squared metric. To avoid overfitting, linear regression models were fitted only when the number of selected features was less than 1000. For each gene, we have performed 70 different feature selection methods using various cutoffs and approaches as explained above. The performance of each model was

evaluated by calculated average cross-validation correlation coefficient, *r*, between predicted and observed expression values in the corresponding hold-out (e.g., test) data from each fold.

Prediction Models

We trained models to predict expression of individual brain genes from blood gene expression. The dependent variable y is the TMM-normalized, $\log_2$-transformed expression of a single brain gene, and residualized for sex and sequencing covariates (SMCENTER, SMRIN, SMTSISCH, SMEXNCRT, SMRRNART, SMNTERRT). The features X are normalized expression levels of all blood genes.

Linear regression models were defined as:

$$y_{brain} = \beta_0 + \sum_{i=1}^{p} \beta_i x_{blood,i} + \epsilon$$

where β_i are regression coefficient, p is the number of blood features, and ϵ is residual error.

Elastic net models extended this formulation with L1/L2 regularization to improve prediction stability for high correlated features:

$$\hat{\beta} = \arg\min_{\beta} \left\{ \frac{1}{2n} \sum_{j=1}^{n} \left(y_j - \beta_0 - \sum_{i=1}^{p} \beta_i x_{ji} \right)^2 + \lambda \left(\alpha \sum_{i=1}^{p} |\beta_i| + \frac{1-\alpha}{2} \sum_{i=1}^{p} \beta_i^2 \right) \right\}$$

where λ controls regularization strength and $\alpha \in [0,1]$ balances L1 (lasso) and L2 (ridge) penalties. Optimal α and λ were selected using 10-fold cross-validation, testing α values from 0 to 1 in increments of 0.1. If fitting failed, the model was refit as ridge regression ($\alpha = 0$).

Pathway enrichment

For functional analysis of the genes, we used the R package enrichR with the GO-Biological Process and Reactome databases [19-21].

ROSMAP data

Gene expression data from the dorsolateral prefrontal cortex in the ROSMAP cohort were obtained from RNA-seq and corrected for postmortem interval (PMI), RNA integrity number (RIN), sex, study, and batch effects. Monocyte gene expression data were corrected for exonic rate, sex, study, and batch effects, with 279 individuals having paired monocyte and brain expression data [22].

Results

Distinct Information Captured by Supervised and Unsupervised Feature Selection

To identify the most effective feature selection mechanism for brain gene expression, we utilized four different approaches within the training sets from 5-fold cross validation (CV). We first applied principal component analysis (PCA) on the blood transcriptome data and used the top principal components (PCs) as features for predicting gene expression in the brain tissues (i.e., unsupervised feature selection). The top 40, 80, 100, and 120 PCs as well as the PCs that explain 80%, 85%, 90%, and 95% of the variance (denotated as gPC(.)) were selected as features.

As an alternative approach, we also identified blood genes whose gene expression profiles were correlated with a target brain gene (i.e., supervised feature selection) by Pearson's correlation coefficient thresholds of $|r|>0.2$, 0.25, and 0.3, as well as $r>0.2$, 0.25, 0.3 (denoted as RG(.)). Thirdly, we extracted the module features by selecting the top 1, 2, 5, and 10 PCs, and the PCs that explained 70%, 75%, 80%, and 85% of the variance of each co-expressed gene module

(denotated as mPC(.)). Lastly, we combined the module features with blood genes that were highly correlated with a given brain gene.

Prediction accuracy was assessed as the average correlation between the predicted brain gene expression values and the actual brain gene expression values across 5 folds. The number of predicted genes (with an average CV correlation coefficient, $r > 0.1$) varied by brain region and feature selection method. When using the top-performing method for each region, the number of predicted genes ranged from 10,583 in hippocampus to 15,253 in the cerebellum (PAXgene-preserved), with corresponding method-specific configurations noted in Table S2. The number of genes predicted with moderate accuracy ($r \geq 0.6$) ranged from 65 to 134, with the highest count observed in the cerebellum using a combination of correlation-based and module-derived features. High confidence predictions ($r \geq 0.9$) were achieved for 2 to 10 genes per region, again most frequently in the cerebellar hemisphere.

To more systematically evaluate prediction performance across accuracy thresholds, we quantified the number of predicted genes at nine r thresholds (0.1 through 0.9) across brain regions (Fig. S1). When using only the top-performing feature selection method, the cerebellum (PAXgene-preserved) and cortex (PAXgene-preserved) showed the highest number of predicted genes with $r > 0.1$ (15,253 and 15,136, respectively), while the putamen and hippocampus showed the lowest (11,323 and 10,583, respectively). At $r > 0.5$, prediction counts ranged from 363 in the cerebellum to 130 in the anterior cingulate cortex. At the highest accuracy threshold ($r > 0.9$), gene counts ranged from 10 in the cerebellar hemisphere to 2 in the anterior cingulate. When aggregating predictions from the top five feature selection methods, these numbers increased overall, with the substantia nigra and hypothalamus showing the most genes at $r > 0.1$ (18,335 and 18,100, respectively), and the hippocampus showing the least (16,373). For $r > 0.5$,

the cerebellum again had the highest count (653), and the putamen the lowest (202). At $r > 0.9$, the cerebellar hemisphere had the most genes (13), and the anterior cingulate cortex the fewest (2). One contributing factor to the variation in predictive performance across brain regions may be the number of available paired samples, which is highest for the cortex, cerebellum, caudate, and cerebellar hemisphere (Figure 2A), potentially enhancing model robustness and prediction accuracy in these regions.

Overall, unsupervised principal component-based feature selection predicted a larger number of genes, but with lower accuracy (average CV r between 0.1 and 0.6), whereas correlation-based feature selection predicted fewer genes, but with much higher accuracy ($r \geq 0.6$). Figure 2B shows these trends in the frontal cortex, and Figure 2C how different feature selection strategies contribute across accuracy thresholds, suggesting that integrating supervised and unsupervised approaches can improve predictive power.

To ensure a direct comparison with the recently developed BrainGENIE method[15], which uses the top 40 blood PCs within a linear modeling framework for all genes, we trained a linear model using the top 40 PCs and identical training and testing splits as those used for IPS. Under this matched setting, IPS consistently yielded a higher number of predicted genes (avg CV $r > 0.1$) across all brain tissues (Figure 3A). For example, in the cerebellum 15,247 genes were predicted by using the top 120 PCs (e.g., gPC(120PC)), whereas BrainGENIE predicted only 10,048 genes. We further compared the distribution of cross-validation correlation coefficients (Figure 3B), which shows that IPS is consistently shifted toward higher CV r values relative to BrainGENIE, demonstrating improved prediction performance across genes.

In addition to this direct comparison, we examined previously published approaches, including TEEBoT[13] and B-GEX[14]. Both methods were trained on earlier GTEx releases with smaller

sample sizes, and direct retraining under identical conditions was not feasible. Available results were limited to two regions for TEEBoT (caudate and cerebellum) and one region for B-GEX (cerebellum). Across all evaluated accuracy thresholds ($r > 0.0$–0.9), IPS consistently predicted substantially more genes than TEEBoT in both caudate and cerebellum. For example, at a moderate threshold ($r > 0.3$), IPS predicted 4,153 vs. 344 genes in caudate and 7,930 vs. 1,241 in cerebellum, with this advantage persisting even at stringent thresholds (e.g., $r > 0.8$). For cerebellum, B-GEX reported a mean gene-level correlation of 0.044, which was substantially lower than that achieved by our integrated IPS framework (0.286). While these comparisons are constrained by differences in training data versions and available outputs, they further support the improved performance of IPS.

While more complex nonlinear models can theoretically capture higher-order interactions, our comparisons showed no performance advantage over linear or elastic net models. We evaluated these approaches using hippocampus data under a simplified setting that excluded network module features to reduce both model and computational complexity. As shown in Table S3, these comparisons did not demonstrate a performance advantage over linear or elastic net models. Given the limited sample size and correlated transcriptomic features, linear models provided a stable solution in this context.

Blood Network Features Enhance the Accuracy of Predictions for Specific Genes

We have constructed a blood tissue co-expression network using MEGENA and determined the top PCs of each co-expression network module as module features (i.e., mPC(.)). We have determined the module eigengene (the first PC) as well the first 2, 5, and 10 PCs, and the PCs that explained 70%, 75%, 80%, and 85% of the variance of each module. The prediction accuracy for genes, ranging from 2,395 to 3,043, with an average CV correlation $r>0.1$, was

increased by at least 5% across different brain regions when module features were used (Table S4). Figure 3C illustrates the genes that showed the most notable improvement in prediction accuracy within the frontal cortex.

We then assessed whether integrating features derived directly from the blood transcriptome (e.g., global features) with those derived from the blood network (e.g., module features) improves predictive accuracy for specific genes. This approach improved the prediction accuracy for a greater number of genes. Specifically, the accuracy of genes, ranging from 6,228 to 8,777, with an average CV $r>0.1$, was increased by at least 5% across all brain regions (Table S5). Figure 3D illustrates the genes that showed the most notable improvement in prediction accuracy within the frontal cortex.

Overall, the combination of module features and global features had the best performance by predicting half of the profiled genes with $r >0.1$, while global features alone accounted for 35% of the genes, and module features alone contributed to 15% across all the brain regions (Figure 4A). Figures 4B show the performance of different gene sets, highlighting the feature sets that yielded the best results for each. Utilizing different feature sets and feature selection approaches capture complementary, orthogonal information, each contributing to the prediction of different genes.

Gene-Specific Power of Different Feature Selection Methods

To evaluate the performance of all proposed feature selection methods, we identified the top 5 methods with the highest number of predicted genes with an average CV $r>0.5$. Rather than relying on a single feature selection strategy, we assessed whether combining multiple approaches would improve predictive coverage. Specifically, for each gene we selected the best-

performing method among the top five feature selection approaches. This combined strategy increased the number of well-predicted genes (r>0.5) compared to using any single method alone. The effect of combining an increasing number of feature selection methods (from 1 to 5) on the number of predicted genes across brain regions is shown in Figure S1. For 10/12 brain regions, the feature selection method predicting the most genes with r>0.5 was the combination of module and global features (Figure S2). The unsupervised (PCA) method applied on blood transcriptome was the top method for the remaining 2/12 brain regions (spinal cord and substantia nigra) and was the second best for the 9/12 regions. Interestingly the second-best method was the supervised feature selection for amygdala.

Overall, the ranking of the top five methods varied across brain regions, indicating that no single feature selection strategy consistently dominated across tissues. Instead, different approaches captured complementary predictive signals. Supplementary Figure S2 summarizes these results using a Venn diagram representation of the five top-performing feature selection methods, illustrating their overlap and relative ranking across brain regions. In the figure, the method predicting the largest number of genes with average CV r>0.5 is highlighted in red, followed by the second-best in green, the third in purple, the fourth in blue, and the fifth in orange.

Supplementary Table S6 provides a detailed breakdown of the feature combinations and gene prediction performance across brain tissues. For each tissue and correlation threshold (accuracy cutoff; mean CV r), the table reports the number of genes predicted above the specified cutoff when combining different numbers of top-performing feature selection methods (1–5 methods). For each combination level, predictions were generated by selecting, for each gene, the best-performing method among the top-ranked methods for that tissue. The table also lists the specific feature selection method(s) included in each combination.

Consistent with the results summarized in Supplementary Figures S1 and S2, these results highlight that different feature selection strategies capture complementary predictive signals. As a result, combining multiple top-performing approaches increases the number of genes that can be accurately predicted (r>0.5) compared to relying on a single method alone.

Notably, each gene-brain region model was trained using paired blood and brain expression data specific to that tissue, which optimizes performance for the corresponding region. Because regulatory relationships and co-expression patterns differ across brain regions, applying models trained in one region to another is not recommended.

Preservation of Age-Associated Expression Patterns in Predicted Gene Profiles

As a representative analysis, we examined whether our predictive models preserve age-associated gene expression patterns in the hippocampus. To evaluate how preservation of age associated expression patterns depends on prediction accuracy, we stratified genes into three groups based on cross-validated prediction performance (CV r): poorly predicted (CV r < 0.1; n=536), moderately predicted (0.1 ≤ CV r ≤ 0.5; n=18,753), and well-predicted (CV r > 0.5, n=353). For each gene, we computed the correlation between observed expression and age (r_1) and between predicted expression and age (r_2).

Correspondence between observed and predicted age associations increased with prediction accuracy: poorly predicted genes had r = 0.195, moderately predicted genes r = 0.296, and well-predicted genes r = 0.496 (Supplementary Table S7). Similarly, the mean absolute difference between r_1 and r_2 decreased from 0.10 in poorly predicted genes to 0.06 in well-predicted genes. These results demonstrate that predicted expression preserves age-associated patterns primarily for the genes with higher prediction accuracy, while preservation is weaker for poorly predicted

genes. The relationship between r_1 and r_2 is visualized in Figure 5A, highlighting the consistency between observed and predicted age associations for well-predicted (CV r> 0.5) genes. This analysis supports the interpretation that age-related variation is partially retained in predicted expression and highlights the dependency on prediction quality. While this analysis was conducted in the hippocampus, the same approach can be extended to other brain regions as well as to other phenotypes beyond age, such as disease status.

Immune-related Genes Exhibit Greater Predictive Power

To assess biological functions of the accurately predicted genes (r>0.5) in each brain region, we performed pathway enrichment analysis using EnrichR[19]. On average 565 genes were predicted with r>0.5 across all brain regions, with the number of predicted genes ranging from 273 in the putamen to 976 in the substantia nigra (Figure 5B). We observed significant enrichment in immune-related pathways, including cytokine-mediated signaling, interleukin signaling, interferon signaling, and response to cytokine, across most brain regions (Figure 5C-D). The enrichment was strongest in the cortex (right cerebral frontal pole cortex, sampled at donor collection site and preserved in PAXgene fixative) and frontal cortex (right cerebral frontal pole cortex, sampled at Miami Brain Bank and preserved as fresh frozen tissue). One potential explanation for this observation is the interaction between the brain and peripheral immune systems, which share common pathways and mechanisms, thereby leading to correlated changes in gene expression. Additionally, it is known that systemic immune cells, which can mobilize and directly infiltrate the brain parenchyma—influence gene expression in brain resident immune cells responses.

Top Blood-based Predictors

To better understand the interactions between the blood biomarkers, and the brain genes, we assessed the most informative features of the brain genes with highest prediction accuracy. Initially, the top 10 brain genes exhibiting the highest accuracy in the frontal cortex were identified, including *NPIPB15, GSTM1, ENSG00000213058, RPS14P1, ENSG00000197582, GATD3, RPL13P12, TBC1D3, LINC01291, LOC102724159,* with average cross-validation correlation coefficients (CV r) ranging from 0.89 to 0.94. Similarly, the top 10 genes with the highest accuracy in the hippocampus were determined, namely *GSTM1, NPIPB15, GATD3, ENSG00000213058, RPS14P1, LINC01291, TBC1D3, ENSG00000262539, RPL13P12, LOC102724023,* with average CV r values between 0.86 to 0.95. Subsequently, for each gene, the top blood biomarkers were identified based on their correlation with the target brain gene ($|r|>0.2$) within each cross-validation fold. The top blood-based predictors were defined as those selected in at least two out of five CV folds. Pathway enrichment analysis was then performed on these top features (Figure 6). The results showed that the blood predictors for the genes *GATD3* and *LOC102724159* were significantly enriched in immune response-related pathways, including immunoglobulin-mediated immune response and antigen processing, which might suggest a role in immune response. The blood predictors for *GSTM1* and *NPIPB15* were enriched in pathways associated with muscle contraction. Additionally, the blood predictors of the gene *TBC1D3*, a gene promoting dendritic arborization and protracting the pace of synaptogenesis [23], were enriched in the synaptic signaling pathways, while those for *RPL13P12* were enriched in biosynthetic process. Finally, the blood predictors for *RPS14P1* were enriched in pathways related to neutrophil degranulation and innate immune system. These results highlight the complex interactions between peripheral biomarkers and brain functions.

Prediction Performance Is Not Driven by Gene Expression Abundance

To assess whether prediction performance was influenced by gene expression abundance, we examined the relationship between gene expression level and prediction accuracy across brain regions. For each region, we computed the correlation between gene expression abundance (mean expression level across samples) and cross-validation performance (CV r), separately using gene expression levels measured in blood and in the corresponding brain tissue (Supplementary Figures S3 and S4). Across all regions, the correlation between expression abundance and prediction accuracy was negligible ($|r| < 0.1$), indicating that predictive performance is not systematically driven by highly expressed genes. Notably, top-performing genes exhibited moderate expression levels in both blood and brain tissues.

Application of the Prediction Pipeline to the ROSMAP Cohort

To assess the behavior of our framework in an independent dataset with a different experimental context, we applied the same pipeline to the ROSMAP cohort using monocyte RNA-seq data as predictors [22]. Brain expression in ROSMAP was derived from the dorsolateral prefrontal cortex, while in GTEx we used frontal cortex (BA9, snap frozen) samples. These regions are anatomically similar but not perfectly matched. Importantly, this analysis does not constitute an external validation, as the predictor modality (monocytes vs whole blood), cohort characteristics (AD and controls vs healthy individuals), and potential differences in tissue sampling and processing remain.

Using identical feature selection procedures and 5 fold CV, we evaluated prediction performance across correlation thresholds (Figure S5-A). Across all thresholds, the number of predictable genes was constantly higher in GTEx than in ROSMAP. These differences likely reflect a combination of factors, including the broader cellular composition captured in whole blood relative to monocytes. The results highlight the sensitivity of prediction performance to both

biological context and study design, while demonstrating that the proposed pipeline can be flexibly applied across datasets.

To further evaluate the consistency of gene-level predictions across datasets, we examined the overlap between the top-ranked predicted genes in GTEx and ROSMAP. Across multiple cutoffs (top 10 to top 2000 genes), the number of overlapping genes exceeded expectations under a null model, with statistically significant enrichment observed at all thresholds (Figure S5-B). For example, 13 genes overlapped among the top 50 ($p=1.19 \times 10^{-24}$), and 236 genes overlapped among the top 2000 ($p=1.26 \times 10^{-7}$). This enrichment indicates that, despite differences in datasets, a subset of genes is consistently well-predicted across both datasets.

Prediction Performance of the Alzheimer's Disease Related Genes

To assess the predictive capacity of our models for brain expression of AD-related genes, we evaluated the prediction accuracy of the top 1,000 AD key drivers previously identified in postmortem para-hippocampal gyrus (PHG) samples from the Mount Sinai Brain Bank (MSBB) AD cohort[24]. The analysis was intended to assess the baseline predictability of AD-related genes, rather than to evaluate disease-specific expression changes or external cohort generalizability.

Among those genes, 341 genes exhibited an average CV $r>0.1$, with 14 genes reaching $r>0.5$ in the hippocampus (Figure 7A-B). The IL-4 receptor (*IL4R*) gene demonstrated the highest prediction accuracy with average CV r of 0.61. *IL4R* is expressed on microglia and plays a crucial role in regulating microglial phenotype[25, 26]. It has been proposed that IL-4, a ligand for IL4R, may have a protective role in AD by regulating neuroinflammation and amyloid-beta pathology[27].

Another well predicted AD-related gene is the *TNFRSF1B* gene (avg CV r=0.51), which encodes a protein that is a member of the TNF-receptor superfamily. Genetic variants in *TNFRSF1B* have been associated with cognitive resilience in AD[28]. To further explore the functional context of these genes, we generated hierarchical co-expression networks centered on *IL4R* and *TNFRSF1B* in the hippocampus using the MSBB dataset. These networks were constructed at two levels (layer 1 and layer 2 from *IL4R* or *TNFRSF1B*), reflecting immediate and extended co-expression relationships. We assessed the functional relevance of genes withing each network layer by evaluating their predictive performance and association with AD pathology. Specifically, we examined the previously established AD associations of these network genes as identified through prior analyses of differential expression patterns across clinical traits, including Clinical Dementia Rating (CDR), Braak & Braak score (bbscore), CERAD score, and plaque density, in AD patients versus controls from the MSBB dataset[24]. To illustrate the model's performance, we highlighted a representative layer 2 network associated with CDR for *IL4R,* which is also closely connected to *TNFRSF1B,* in Figure 7C. These examples demonstrate that the genes that overlap with AD molecular signatures have good prediction performance (172/182 network genes were predicted with average CV r>0.1). Comprehensive results for all network layers and traits are provided in the Supplementary Figures. Full panels of predictive performance plots for *IL4R* (8 conditions: 2 layers × 4 traits) are shown in Figure S6, and corresponding analyses for *TNFRSF1B* are included in Figure S7. Additionally, to further validate the predictive accuracy of our model at the individual gene level, Figure S8 presents observed versus predicted gene expression values in the hippocampus for the four most predictable genes from the 1,000 AD key drivers list: *IL4R*, *CSDA*, *MAFF*, and BCL6. These plots highlight the model's ability to accurately capture gene-specific expression patterns relevant to AD biology.

Discussion

Understanding the molecular processes in the brain is crucial for improving our knowledge of brain disorders, as gene expression in the brain plays an important role in the pathogenesis of both neurological (i.e., neurodegenerative) and psychiatric diseases. However, direct investigation of brain tissue remains limited due to the invasive nature of sample collection. Consequently, there is a pressing need for non-invasive biomarkers that can serve as reliable proxies for brain gene expression.

In this study, we explored the potential of blood-derived transcriptomic signatures to predict brain gene expression. Blood samples offer a minimally invasive and widely accessible source, making them an attractive alternative for large-scale studies and longitudinal monitoring. By integrating blood transcriptome data with a variety of feature selection techniques, we developed gene-specific predictive models. This gene-specific approach enables the identification of blood biomarkers that are closely tied to specific molecular processes in the brain, offering improved interpretability and potential clinical utility.

The proposed framework is intentionally model-agnostic and flexible, allowing for the integration of diverse machine learning approaches depending on the specific research context. Although linear models performed well in our analyses, particularly at higher correlation thresholds, we do not advocate for a single modeling strategy. Rather, the central contribution of this study is the demonstration that gene-specific feature selection and model selection can substantially improve predictive accuracy compared to conventional genome-wide, one-size-fits-all approaches. This flexibility enables the pipeline to accommodate alternative models, which may further enhance performance for specific genes or datasets.

Despite promising results, several challenges remain. One key challenge is the heterogeneity of whole blood. The complex interactions between different cell types could dilute or conceal the signals that are informative of brain gene activity. Incorporating cell-type deconvolution methods may help improve the signal-to-noise ratio and enhance model performance.

Another major limitation is the lack of independent cohorts with matched brain and blood expression data, which prevents direct external validation of the proposed models. The availability of such matched samples would enable more robust validation of the predictive models and offer a clearer understanding of cross-tissue gene expression relationships. While we leveraged large-scale, publicly available datasets for model training and evaluation, future efforts should prioritize collecting matched multi-tissue datasets, particularly in specific disease contexts, to support more rigorous benchmarking. More broadly, these results highlight the importance of accounting for dataset shift and avoiding over-reliance on performance within a single dataset, as cross-validation alone does not guarantee generalizability to independent or clinical cohorts [29].

Despite this limitation, our findings demonstrate that blood-based transcriptomic models can provide a research tool for inferring brain gene expression, highlighting their potential utility for studying neurological disorders. While these models show promise, their performance in independent or clinical cohorts remains to be established, and external validation in appropriately matched blood and brain samples will be necessary before they can be considered for diagnostic or prognostic applications. Future studies may explore their use in identifying early molecular signatures of disorders such as AD, PD, and major depressive disorder, or for non-invasive monitoring of disease progression, but such applications remain speculative at this stage.

Additionally, although immune-related genes emerged as strong predictors of brain gene expression, the directionality and causality of these associations remain to be clarified. It is plausible that peripheral immune activity reflects or influences neuroinflammatory processes, which are increasingly recognized as key contributors to neurodegenerative disease. Indeed, controlled studies in rodents have now shown the dynamic interactions between peripheral immune compartments and the brain which support brain-body directionality and causality [30]. Further investigation into the shared regulatory mechanisms between the immune system and the brain will be essential to untangle these relationships.

Our findings underscore the feasibility of using blood-based transcriptomic models to infer brain gene expression and lay the groundwork for future diagnostic and prognostic tools. If validated in clinical cohorts, these models could be employed to identify early molecular signatures of neurological disorders such as AD, PD, and major depressive disorders. Moreover, they may enable non-invasive monitoring of disease progression or response to therapy, offering valuable insights for precision medicine.

Looking ahead, integrating additional layers of omics data—such as epigenetic modifications, proteomics, and metabolomics—may further refine prediction accuracy and enhance biological relevance. Incorporating longitudinal blood samples could also reveal dynamic transcriptomic changes associated with disease progression or treatment. Ultimately, expanding our understanding of peripheral-brain tissue interactions has the potential to bridge a gap in neurodegenerative research and improve clinical outcomes through earlier and more precise intervention.

Availability of source code and requirements

Project name: Blood Biomarkers of Brain Gene Expression

Project home page: https://gitlab.com/csbayrak/ips

Operating system(s): Platform independent

Programming language: R

Other requirements: R 4.2 or higher

License: Data files for examples are distributed under the CC0 1.0 Universal (CC0 1.0) Public Domain Dedication, all code is distributed under the MIT license.

RRID: SCR_027608

Data availability

The gene expression data and the associated sample information are publicly available through the GTEx Consortium portal. Specifically, we used GTEx Analysis V8 bulk tissue RNA-seq gene read count data and the corresponding meta files [16]. The code and input files for an illustrative example are available on GitLab (https://gitlab.com/csbayrak/ips) and a snapshot of the project has been archived on Zenodo under doi:10.5281/zenodo.17477449 [31]. The IPS workflow is registered in WorkflowHub doi:10.48546/workflowhub.workflow.2139.1 [32]. ROSMAP gene expression data were obtained from the Religious Orders Study and Memory and Aging Project cohort and are available through the AMP-AD Knowledge Portal, under controlled access (Synapse accession: syn2580853). DOME-ML annotations can be accessed via the DOME registry (accession xrf3z08hoe) [33].

Acknowledgments

This work was financially supported in parts by grants from the National Institutes of Health (NIH)/National Institute on Aging (UH2AG083258, R01AG085182, RF1AG074010, RF1AG054014, U01AG046170, and R01AG068030).

This work was supported in part through the computational and data resources and staff expertise provided by Scientific Computing and Data at the Icahn School of Medicine at Mount Sinai and supported by the Clinical and Translational Science Awards (CTSA) grant UL1TR004419 from the National Center for Advancing Translational Sciences. Research reported in this publication was also supported by the Office of Research Infrastructure of the National Institutes of Health under award number S10OD026880 and S10OD030463. The content is solely the responsibility of the authors and does not necessarily represent the official views of the National Institutes of Health.

We gratefully acknowledge Yusra Chowdhury and Sophia DeGregorio (Stuyvesant High School, New York, NY), and Efe Guner (Walt Whitman High School, Bethesda, MD) for their contributions to both the preliminary and downstream stages of data analysis.

Author contributions

C.S.B. (Conceptualization, Investigation, Formal Analysis, Software, Methodology, Validation, Data Curation, Writing - Original Draft Preparation, Visualization), Q.Z. (Formal Analysis), M.I. (Data Curation), M.H.W. (Data Curation), S.J.R. (Supervision, Writing - Review & Editing), B.Z. (Conceptualization, Project Administration, Funding Acquisition, Writing - Review & Editing). All authors reviewed and approved the final manuscript.

Declaration of interests

The authors declare no competing interests.

Figure Captions

Figure1. General framework of the Integrative Prediction System (IPS) for predicting brain gene expression using paired blood and brain transcriptome data from the GTEx dataset. The prediction models have been trained on paired blood and brain expression data from the GTEx dataset for each brain tissue via 5-fold cross validation. Predictive features have been generated (i) taking the top principal components, (ii) taking the highly correlated features with the target brain gene, (iii) taking the top principal components of the MEGENA modules of blood tissue. For training, linear regression and elastic models were used. The model prediction accuracy was estimated by calculating the average correlation between predicted and observed brain gene expression values over five folds.

Figure2. Sample overlap and gene prediction performance across feature selection methods. (A) Upset plot showing the number of individuals with paired whole blood and brain tissue samples in GTEx. The plot indicates that more than 100 subjects have both whole blood and brain tissue sample across all brain regions. **(B)** Number of genes predicted at various accuracy cutoffs using different feature selection methods. The x-axis shows the average correlation between the actual and predicted frontal cortex expression derived from blood tissue. gPC(.) represents the features selected by PCA from the blood transcriptome data, mPC(.) represents the module features selected by PCA, and RG(.) represents the features selected based on correlation with target brain gene. IPS indicates the number of genes when best feature selection method was considered per gene. **(C)** Number of predicted genes by different feature selection strategies. The y-axis indicates the number of predicted genes in the frontal cortex. Each plot shows the number of predicted genes at varying levels of prediction accuracy, measured by the average cross-validation correlation, r.

Figure3. Comparative performance and accuracy gains of feature selection strategies for brain gene expression prediction. (A) Comparison of the performance of the proposed method, IPS, with existing approach. The x-axis shows the number of genes predicted with an average CV r>0.1 when using the top 120 PCs from blood transcriptome, BrainGenie (top 40 PCs), and when integrating the results from all 70 feature selection approaches proposed in this study (i.e., IPS). **(B)** Density distributions of CV r across genes for each brain region. Mean differences are reported as mean ± 95% CI. **(C)** Genes showing the greatest improvement in prediction accuracy (>500%) when using only module features compared to using only global features in the frontal cortex. **(D)** Genes showing the greatest improvement in prediction accuracy (>1000%) when using a combination of global and module features versus global features alone in the frontal cortex.

Figure4. Performance of different feature sets. (A) The percentage of genes for which each feature set performed best across different brain regions. **(B)** Performance of different feature selection strategies in the frontal cortex, measured by correlation, for various genes.

Figure5. Analysis of accurately predicted brain genes (r>0.5). (A) Correlation between age-association patterns based on observed vs. predicted gene expression for genes with prediction accuracy CV r > 0.5 in the hippocampus. Each point represents a gene; the x-axis shows correlation with age based on observed expression, and the y-axis shows correlation with age based on predicted expression. **(B)** Number of genes predicted with r>0.5 accuracy across different brain genes. UpSet plot highlights the number of genes that are accurately predicted in multiple brain regions. **(C)** Top canonical pathways of the accurately predicted genes in each brain region. The color intensity indicates the significance of enrichment (-log10 Pvalue). **(D)** Top gene ontology biological processes of the accurately predicted genes in each brain region. The color intensity indicates the significance of enrichment (-log10 Pvalue).

Figure6. Analysis of the top blood-based predictors for brain genes selected based on the top 10 highest prediction accuracies. Pathway enrichment analysis of the top blood biomarkers for genes **(A)** in the frontal cortex and **(B)** in the hippocampus. Blood-based predictors were selected based on their correlation with target brain genes (|r|>0.2) selection in at least 2 out of 5 CV folds. The color intensity represents the significance of the enrichment (-log10 Pvalue).

Figure7. Prediction performance and functional relevance of AD key driver genes in the hippocampus. (A) Cumulative number of top 1,000 Alzheimer's disease (AD) key driver genes achieving varying levels of prediction accuracy in the hippocampus, measured as the average cross-validated correlation (r) between observed and predicted expression. **(B)** Distribution of gene counts across defined prediction accuracy thresholds, highlighting how many genes fall within each performance range. **(C)** Co-expression network of IL4R in the hippocampus. Node color reflects prediction accuracy (darker indicates higher accuracy), and nodes with cyan borders are associated with AD based on Clinical Dementia Rating (CDR).

Supplemental information

Table S1. Demographic characteristics of individuals with paired whole blood and brain tissue samples included in the analysis.

Table S2. Number of predicted genes at various accuracy cutoffs.

Table S3. Number of genes predicted in the hippocampus by different machine learning models across varying accuracy thresholds. Feature selection included principal component analysis (PCA) and correlation-based filtering, excluding network-based features.

Table S4. Number of genes with greater prediction accuracy (>5%) when using only module features compared to global features.

Table S5. Number of genes with greater prediction accuracy (>5%) when using combined features compared to global features.

Table S6. Number of genes predicted above correlation thresholds using combinations of 1–5 methods per brain region.

Table S7. Analysis of age-associated expression patterns. Genes were binned by prediction accuracy (CV r), and the correlation between observed (r_1) and predicted (r_2) age associations was computed for each bin.

Figure S1. Prediction performance across brain regions and feature selection combinations. Each panel displays the number of predicted genes (y-axis) at increasing prediction accuracy thresholds (x-axis, cross-validation correlation coefficient r) for a given number of combined feature selection methods (from 1 to 5). Each line represents a different brain region.

Figure S2. Top 5 feature selection methods based on the number of genes predicted with average CV r>0.5. The method that predicted the most genes is annotated in red, the second in green, the third in purple, the fourth in blue, and the fifth in orange.

Figure S3. Relationship between blood gene expression abundance and prediction accuracy across brain regions. Scatter plots show the association between gene expression abundance measured in blood (x-axis; mean expression across samples) and prediction accuracy (y-axis; cross-validation correlation coefficient, CV r) for each brain region. Each point represents a gene. A linear regression line with 95% confidence interval is shown in red. The Pearson

correlation coefficient between gene abundance and prediction accuracy is displayed in each panel.

Figure S4. Relationship between brain gene expression abundance and prediction accuracy across brain regions. Scatter plots show the association between gene expression abundance measured in the corresponding brain tissue (x-axis; mean expression across samples) and prediction accuracy (y-axis; cross-validation correlation coefficient, CV r) for each brain region. Each point represents a gene. A linear regression line with 95% confidence interval is shown in red. The Pearson correlation coefficient between gene abundance and prediction accuracy is displayed in each panel.

Figure S5. Comparison of gene prediction performance between GTEx and ROSMAP. (A) Number of genes predicted across correlation thresholds ($r > 0.1$ to $r > 0.9$). (B) Overlap of top-ranked genes across datasets at increasing cutoffs; points are colored by $-\log_{10}(p)$, and the red dashed line indicates the expected overlap under the null. Observed overlaps exceed expectation at all thresholds.

Figure S6. Co-expression networks of IL4R in the hippocampus across AD-related traits.

Figure S7. Co-expression networks of TNFRSF1B in the hippocampus across AD-related traits.

Figure S8. Observed versus predicted gene expression in the hippocampus for the four most predictable genes among the 1,000 AD key drivers: IL4R, CSDA, MAFF, and BCL6.

References

1. Feigin, V.L., et al., *The global burden of neurological disorders: translating evidence into policy.* Lancet Neurol, 2020. **19**(3): p. 255-265. https://doi.org/10.1016/S1474-4422(19)30411-9.

2. Alcolea, D., et al., *Blood Biomarkers in Neurodegenerative Diseases: Implications for the Clinical Neurologist.* Neurology, 2023. **101**(4): p. 172-180. https://doi.org/10.1212/WNL.0000000000207193.
3. Oh, H.S., et al., *Organ aging signatures in the plasma proteome track health and disease.* Nature, 2023. **624**(7990): p. 164-172. https://doi.org/10.1038/s41586-023-06802-1.
4. Hawrylycz, M.J., et al., *An anatomically comprehensive atlas of the adult human brain transcriptome.* Nature, 2012. **489**(7416): p. 391-399. https://doi.org/10.1038/nature11405.
5. Danner, B., et al., *Brain banking in the United States and Europe: Importance, challenges, and future trends.* J Neuropathol Exp Neurol, 2024. **83**(4): p. 219-229. https://doi.org/10.1093/jnen/nlae014
6. Mele, M., et al., *Human genomics. The human transcriptome across tissues and individuals.* Science, 2015. **348**(6235): p. 660-5. https://doi.org/10.1126/science.aaa0355.
7. Sonawane, A.R., et al., *Understanding Tissue-Specific Gene Regulation.* Cell Rep, 2017. **21**(4): p. 1077-1088. https://doi.org/10.1016/j.celrep.2017.10.001.
8. Tylee, D.S., D.M. Kawaguchi, and S.J. Glatt, *On the outside, looking in: a review and evaluation of the comparability of blood and brain "-omes".* Am J Med Genet B Neuropsychiatr Genet, 2013. **162B**(7): p. 595-603. https://doi.org/10.1002/ajmg.b.32150.
9. Hess, J.L., et al., *Transcriptome-wide mega-analyses reveal joint dysregulation of immunologic genes and transcription regulators in brain and blood in schizophrenia.* Schizophr Res, 2016. **176**(2-3): p. 114-124. https://doi.org/10.1016/j.schres.2016.07.006.
10. Zaman, S., et al., *A Search for Blood Biomarkers for Autism: Peptoids.* Sci Rep, 2016. **6**: p. 19164. https://doi.org/10.1038/srep19164.
11. Leuzy, A., et al., *Blood-based biomarkers for Alzheimer's disease.* EMBO Mol Med, 2022. **14**(1): p. e14408. *Blood-based biomarkers for Alzheimer's disease.*
12. Chahine, L.M., M.B. Stern, and A. Chen-Plotkin, *Blood-based biomarkers for Parkinson's disease.* Parkinsonism Relat Disord, 2014. **20 Suppl 1**(0 1): p. S99-103. https://doi.org/10.1016/S1353-8020(13)70025-7.
13. Basu, M., et al., *Predicting tissue-specific gene expression from whole blood transcriptome.* Sci Adv, 2021. **7**(14). https://doi.org/10.1126/sciadv.abd6991.
14. Xu, W., et al., *Blood-based multi-tissue gene expression inference with Bayesian ridge regression.* Bioinformatics, 2020. **36**(12): p. 3788-3794. https://doi.org/10.1093/bioinformatics/btaa239.
15. Hess, J.L., et al., *BrainGENIE: The Brain Gene Expression and Network Imputation Engine.* Transl Psychiatry, 2023. **13**(1): p. 98. https://doi.org/10.1038/s41398-023-02390-w.
16. Consortium, G.T., *The GTEx Consortium atlas of genetic regulatory effects across human tissues.* Science, 2020. **369**(6509): p. 1318-1330. https://doi.org/10.1126/science.aaz1776.
17. Song, W.M. and B. Zhang, *Multiscale Embedded Gene Co-expression Network Analysis.* PLoS Comput Biol, 2015. **11**(11): p. e1004574. https://doi.org/10.1371/journal.pcbi.1004574.

18. Robinson, M.D., D.J. McCarthy, and G.K. Smyth, *edgeR: a Bioconductor package for differential expression analysis of digital gene expression data.* Bioinformatics, 2010. **26**(1): p. 139-40. https://doi.org/10.1093/bioinformatics/btp616.
19. Kuleshov, M.V., et al., *Enrichr: a comprehensive gene set enrichment analysis web server 2016 update.* Nucleic Acids Res, 2016. **44**(W1): p. W90-7. https://doi.org/10.1093/nar/gkw377.
20. Gene Ontology, C., et al., *The Gene Ontology knowledgebase in 2023.* Genetics, 2023. **224**(1). https://doi.org/10.1093/genetics/iyad031.
21. Milacic, M., et al., *The Reactome Pathway Knowledgebase 2024.* Nucleic Acids Res, 2024. **52**(D1): p. D672-D678. https://doi.org/10.1093/nar/gkad1025.
22. Bennett, D.A., et al., *Religious Orders Study and Rush Memory and Aging Project.* J Alzheimers Dis, 2018. **64**(s1): p. S161-S189. https://doi.org/10.3233/JAD-179939.
23. Dong, J., et al., *A hominoid-specific signaling axis regulating the tempo of synaptic maturation.* Cell Rep, 2024. **43**(8): p. 114548. https://doi.org/10.1016/j.celrep.2024.114548.
24. Wang, M., et al., *Transformative Network Modeling of Multi-omics Data Reveals Detailed Circuits, Key Regulators, and Potential Therapeutics for Alzheimer's Disease.* Neuron, 2021. **109**(2): p. 257-272 e14. https://doi.org/10.1016/j.neuron.2020.11.002.
25. Gadani, S.P., et al., *IL-4 in the brain: a cytokine to remember.* J Immunol, 2012. **189**(9): p. 4213-9. https://doi.org/10.4049/jimmunol.1202246.
26. Kiyota, T., et al., *CNS expression of anti-inflammatory cytokine interleukin-4 attenuates Alzheimer's disease-like pathogenesis in APP+PS1 bigenic mice.* FASEB J, 2010. **24**(8): p. 3093-102. https://doi.org/10.1096/fj.10-155317.
27. Tang, R.H., R.Q. Qi, and H.Y. Liu, *Interleukin-4 affects microglial autophagic flux.* Neural Regen Res, 2019. **14**(9): p. 1594-1602. https://doi.org/10.4103/1673-5374.255975.
28. Pillai, J.A., et al., *TNFRSF1B Gene Variants and Related Soluble TNFR2 Levels Impact Resilience in Alzheimer's Disease.* Front Aging Neurosci, 2021. **13**: p. 638922. https://doi.org/10.3389/fnagi.2021.638922.
29. Dockes, J., G. Varoquaux, and J.B. Poline, *Preventing dataset shift from breaking machine-learning biomarkers.* Gigascience, 2021. **10**(9). https://doi.org/10.1093/gigascience/giab055.
30. Cathomas, F., et al., *Circulating myeloid-derived MMP8 in stress susceptibility and depression.* Nature, 2024. **626**(8001): p. 1108-1115. https://doi.org/10.1038/s41586-023-07015-2.
31. Sevim Bayrak, C. *Integrative Prediction Strategy (IPS): Blood Biomarkers of Brain Gene Expression*. 2025; Available from: https://doi.org/10.5281/zenodo.17477450.
32. Sevim Bayrak, C. *Integrative Prediction Strategy (IPS): Blood-based Prediction of Brain Gene Expression*. 2026; Available from: https://doi.org/10.48546/workflowhub.workflow.2139.1.
33. Cigdem Sevim Bayrak, Q.Z., Marjan Ilkov, Scott J Russo, Minghui Wang, Bin Zhang. *Leveraging Machine Learning and Network Biology to Uncover Blood*

Biomarkers of Brain Gene Expression. GigaScience 2026; Available from: https://registry.dome-ml.org/review/xrf3z08hoe.

Figure

Click here to access/download;Figure;Figure1_updated.tiff

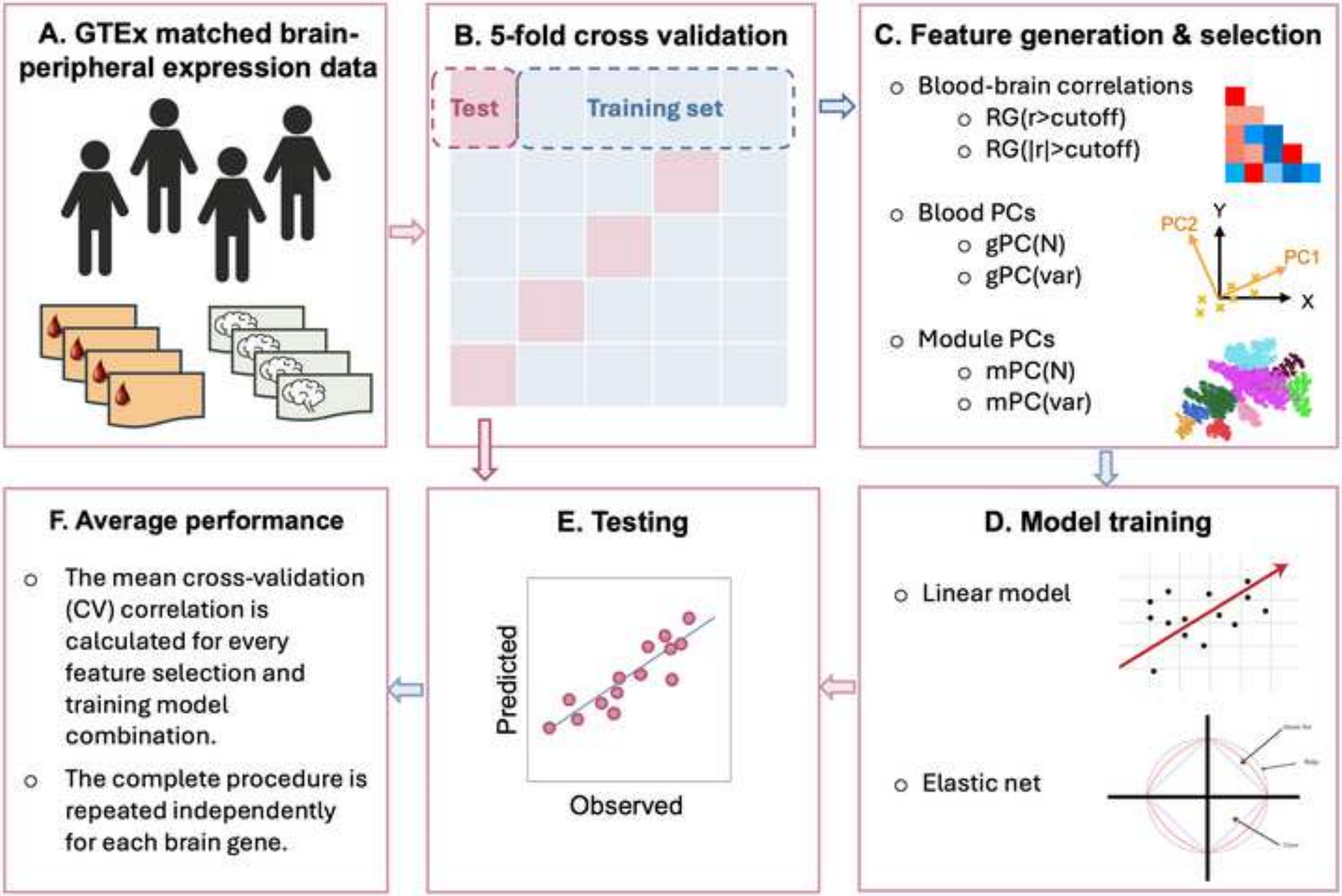

Figure

Click here to access/download;Figure;Figure2.png

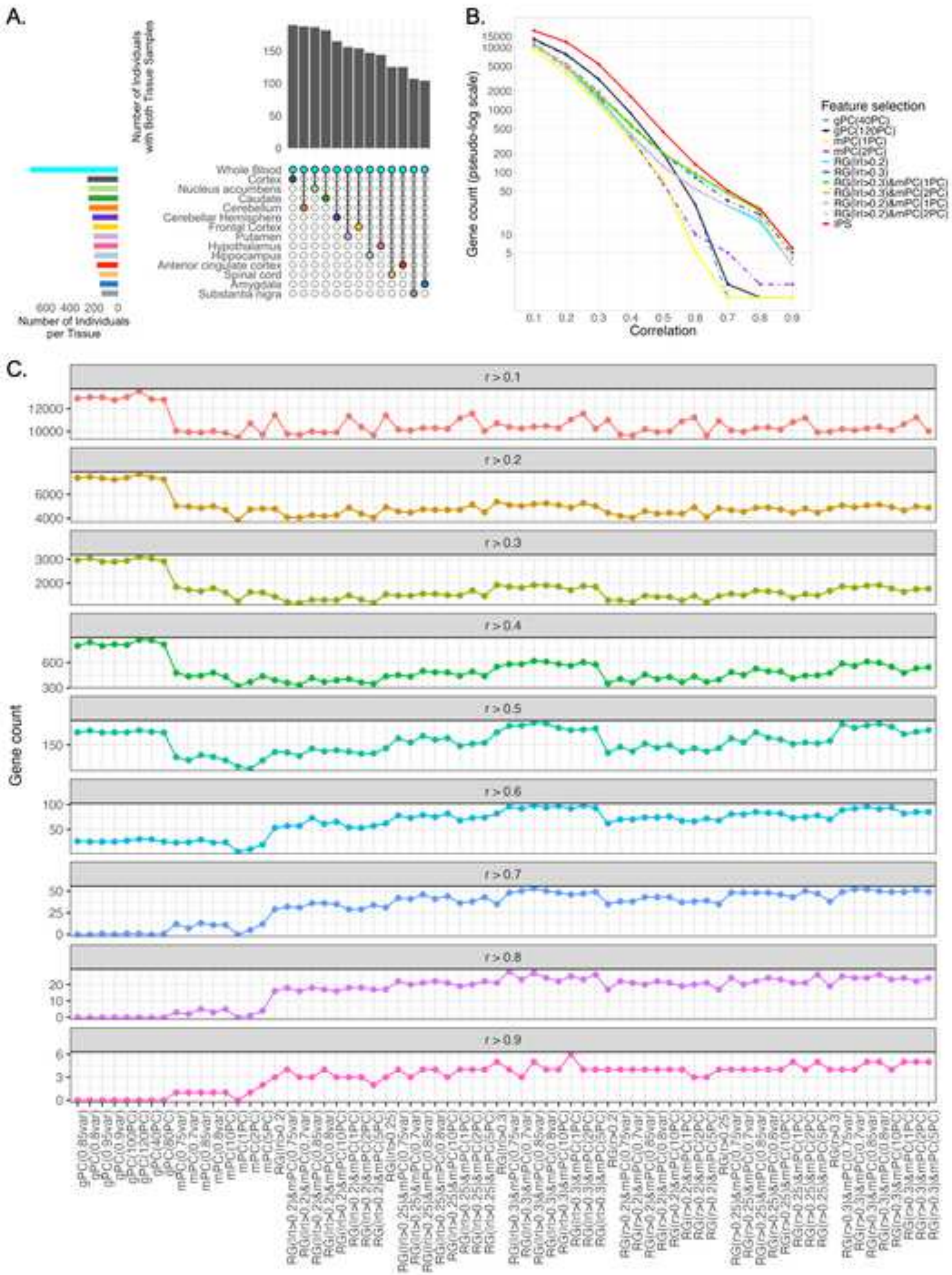

Figure

Click here to access/download;Figure;Figure3.png

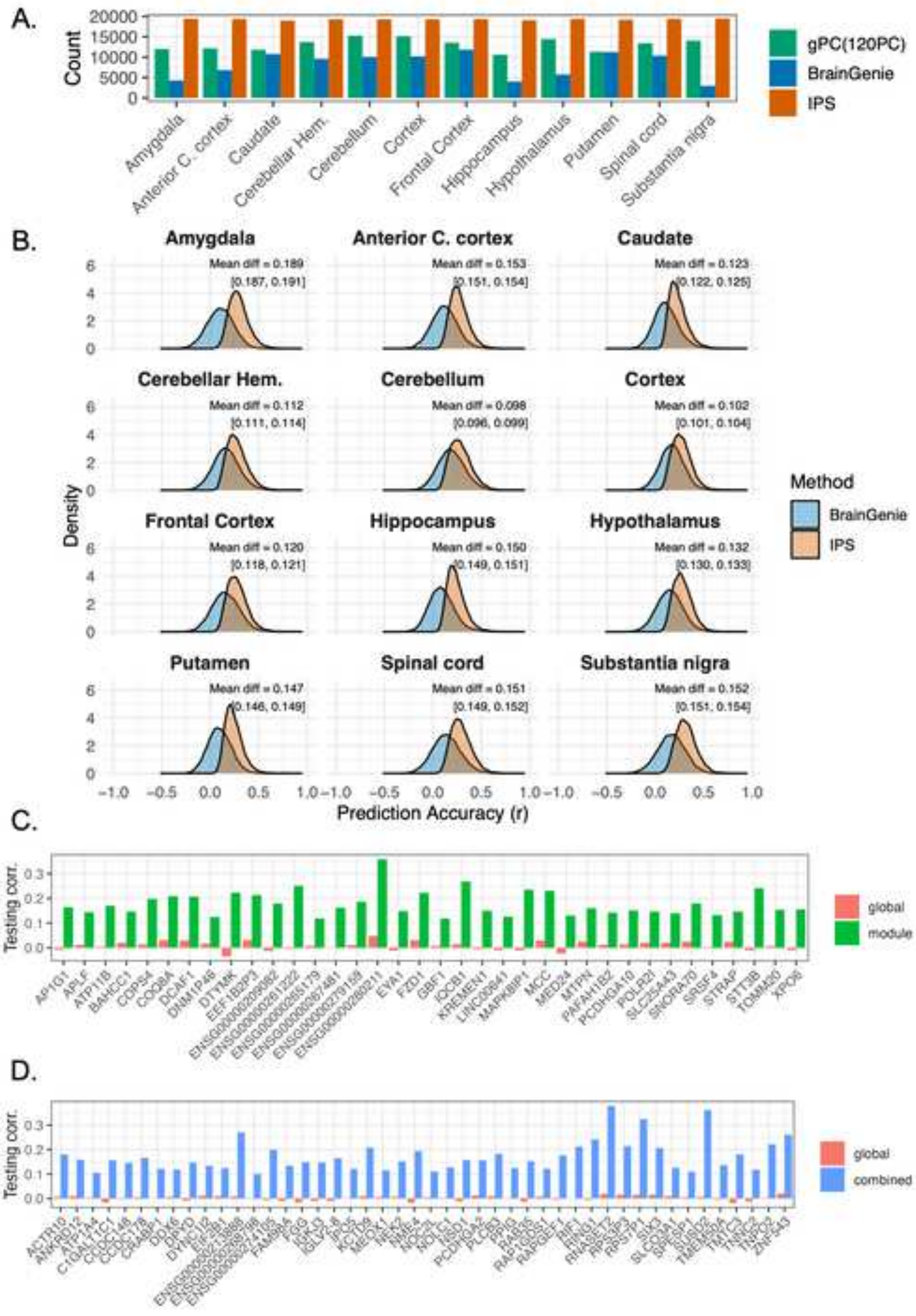

Figure Click here to access/download;Figure;Figure4.png

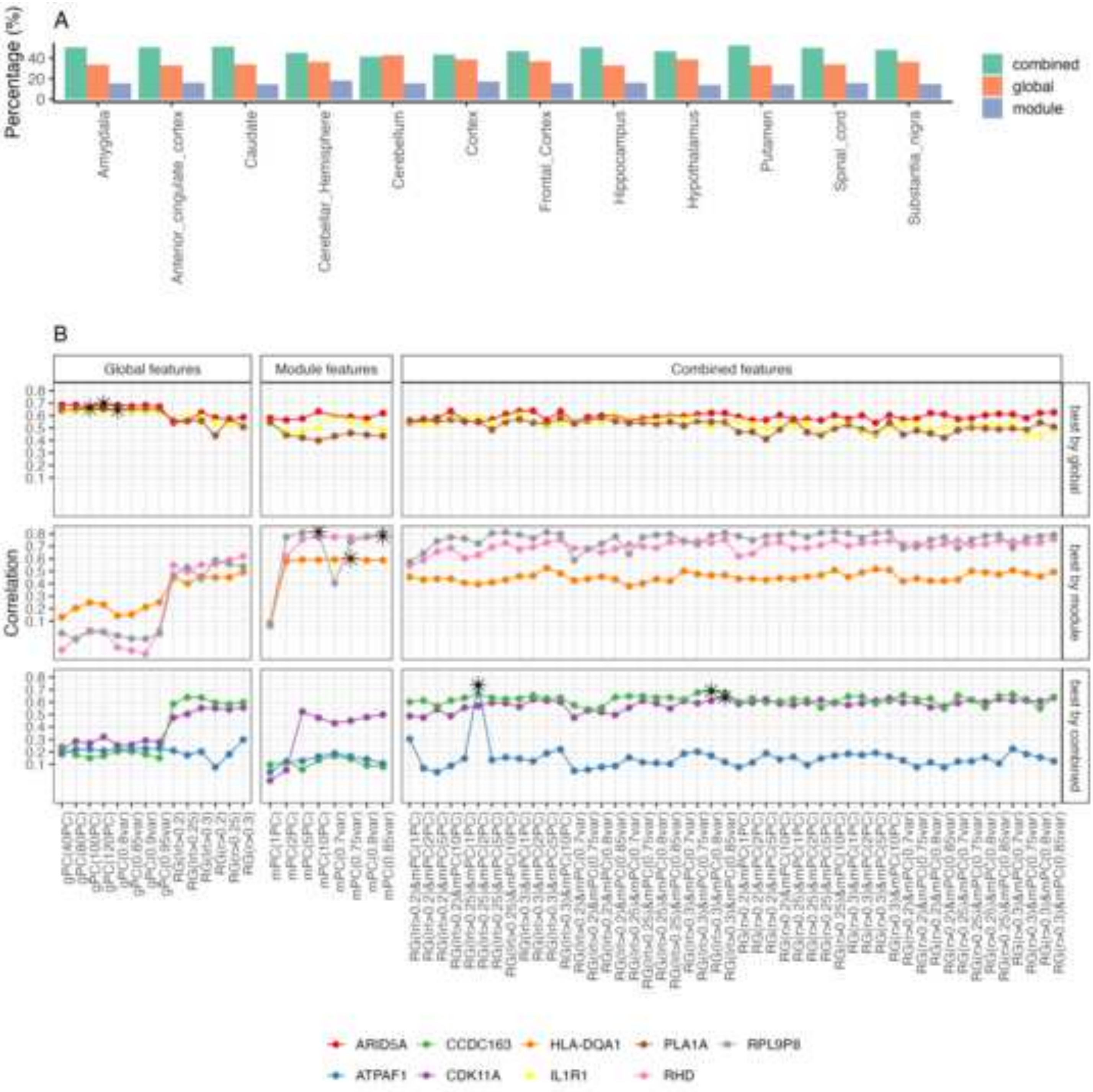

Figure

Click here to access/download;Figure;newFigure5.png

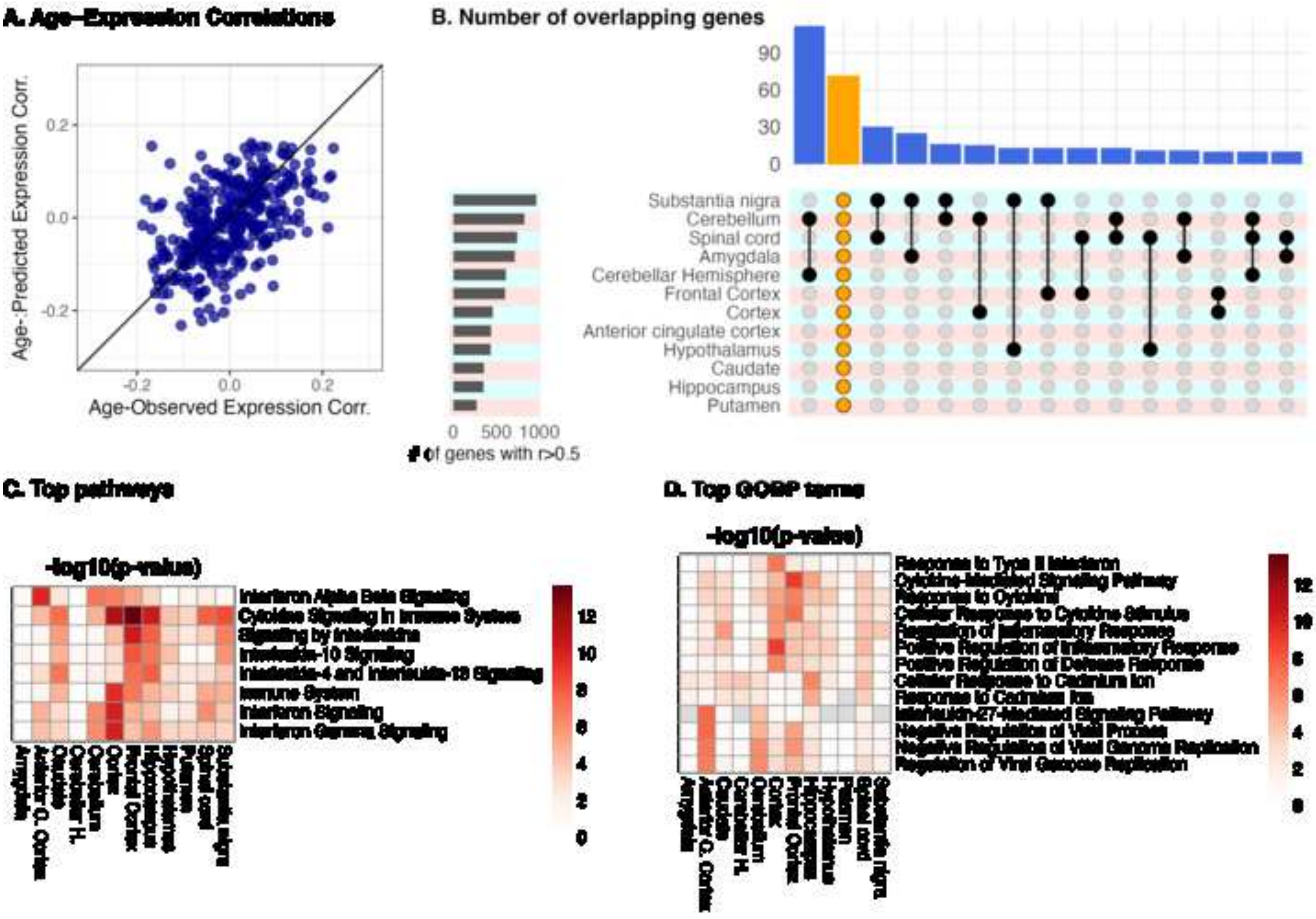

Figure

Click here to access/download;Figure;newFigure6.png

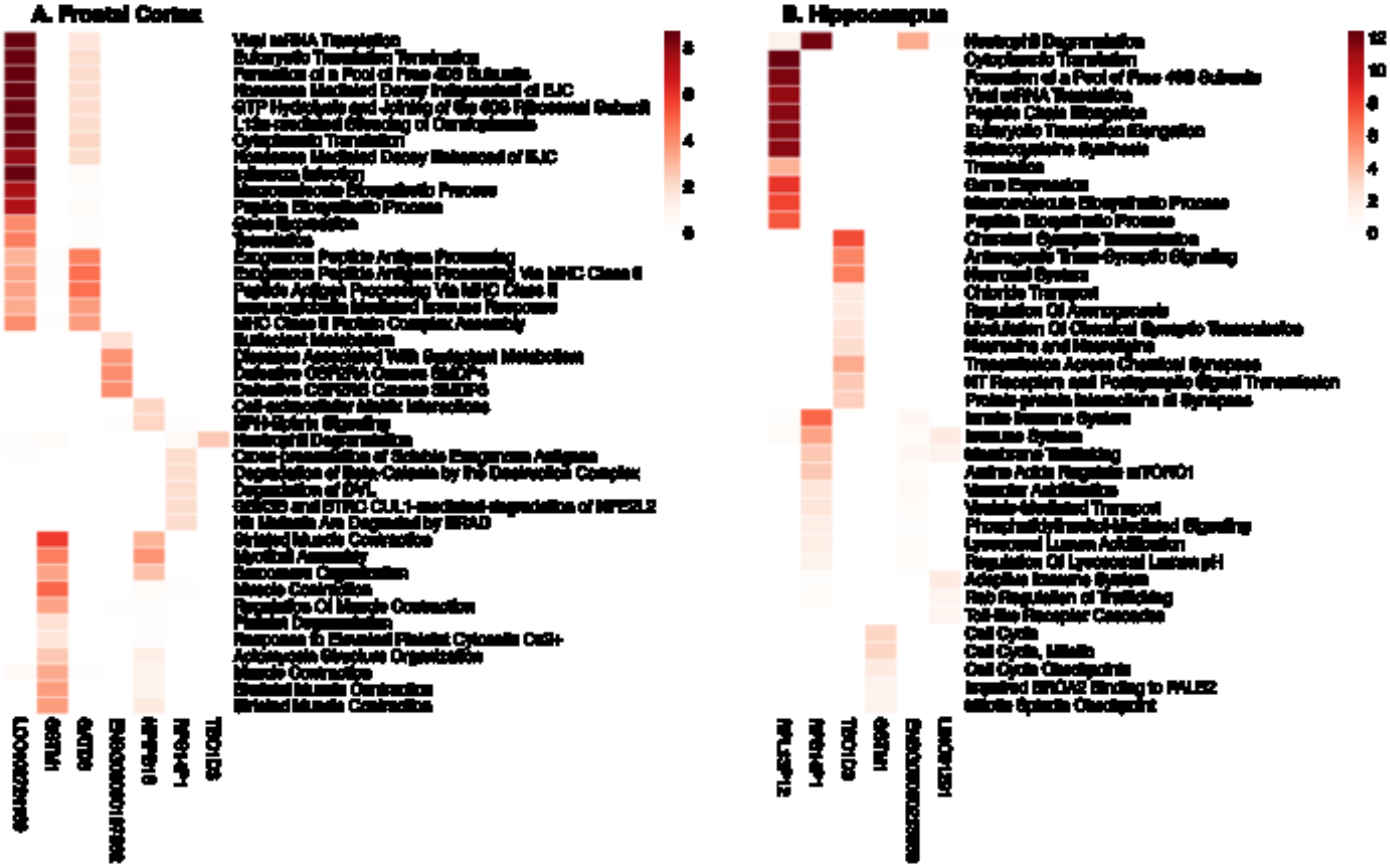

Figure

Click here to access/download;Figure;newFigure7.png

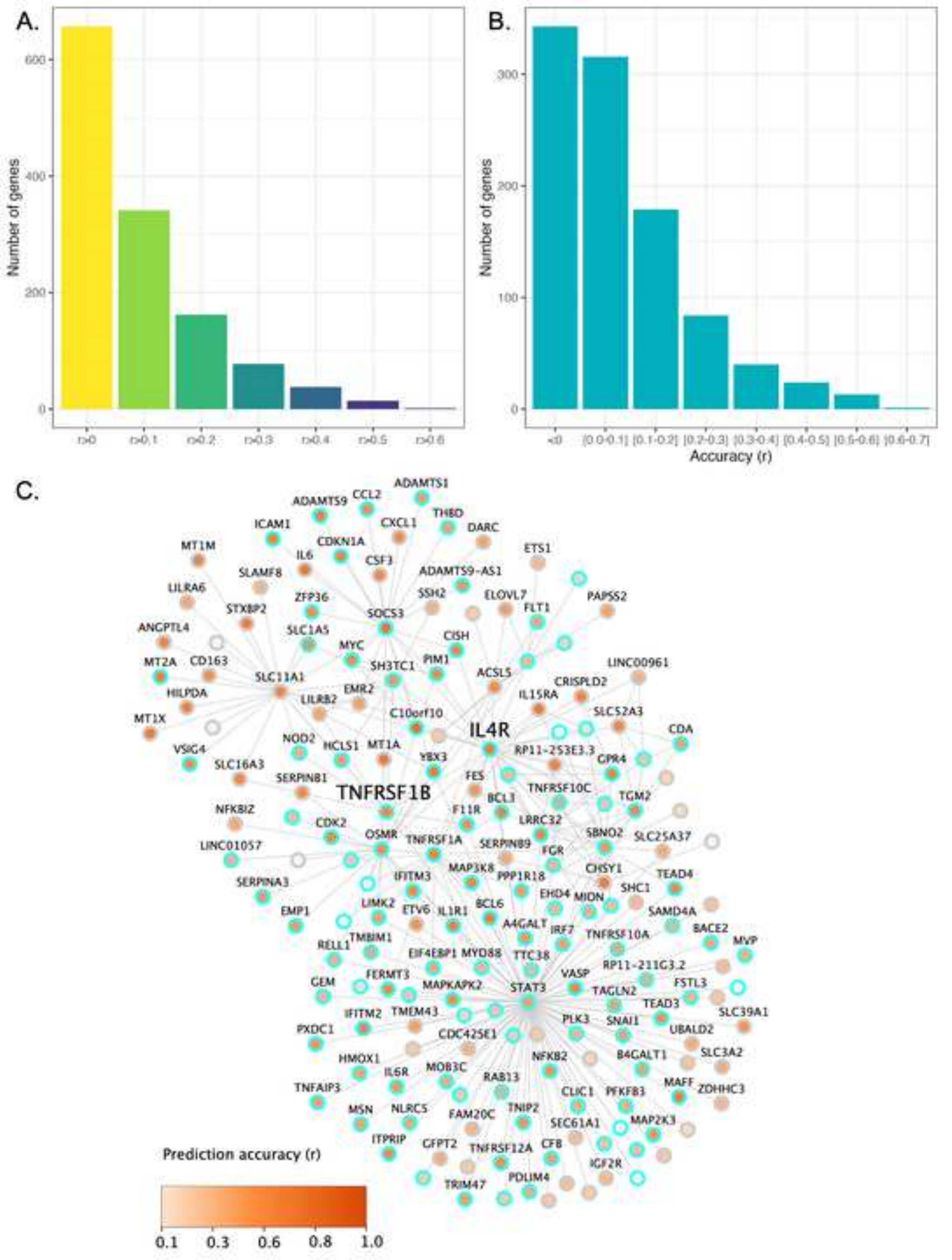

Supplementary Tables

Click here to access/download
Supplementary Material
SupplTables.xlsx

Supplementary Figures

Click here to access/download
Supplementary Material
SupplFigs_May2026.pdf

Personal Cover
Click here to access/download;Personal Cover;CoverLetter_revisions.docx

Icahn School of Medicine at Mount Sinai

Bin Zhang, PhD
Willard T.C. Johnson Research Professor of Neurogenetics
Professor, Departments of Genetics & Genomic Sciences, Pharmacological Sciences, and Artificial Intelligence and Human Health
Director, Mount Sinai Center for Transformative Disease Modeling
Icahn Institute of Genomics, Icahn School of Medicine at Mount Sinai

1425 Madison Avenue (Icahn 3-43)
Box 1498
New York, NY 10029
Phone: 212-659-1726
Fax: 212-659-5507
Email: bin.zhang@mssm.edu

March 26th, 2026

Dear Editors,

We thank you and the reviewers for the careful evaluation of our manuscript, "**Leveraging Machine Learning and Network Biology to Uncover Blood Biomarkers of Brain Gene Expression**" (GIGA-D-25-00434). We appreciate the constructive comments and suggestions, which have greatly helped us improve the clarity, rigor, and reproducibility of our work.

In the revised manuscript, we have:

- clarified the modeling framework with explicit formulations for linear regression and elastic net and included comparisons with alternative machine learning methods (Supplementary Table S3).
- updated analyses on age-associated gene expression, stratified by prediction accuracy (Supplementary Table S7).
- assessed model generalizability using the ROSMAP dataset (Supplementary Figure S8).
- improved all figures and legends, revised the workflow figure for clarity, and added supplementary tables describing sample characteristics and feature combinations.
- moderated claims regarding diagnostic/prognostic applications and clarified limitations regarding external validation.
- updated citations, registered the IPS workflow in WorkflowHub, and included RRID SCR_027608 for the software tool.

We have provided a detailed point-by-point response addressing all the comments. We look forward to hearing from you soon.

Sincerely,

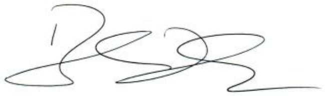

Bin Zhang, PhD
Willard T.C. Johnson Research Professor of Neurogenetics
Professor, Genetics & Genomic Sciences, Pharmacological Sciences, AI & Human Health
Director, Mount Sinai Center for Transformative Disease Modeling
Icahn School of Medicine at Mount Sinai

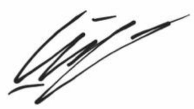

Cigdem Sevim Bayrak, PhD
Assistant Professor, Genetics & Genomic Sciences
Icahn School of Medicine at Mount Sinai

Graphical Abstract

Click here to access/download;Graphical Abstract;graphical_abstract.tiff

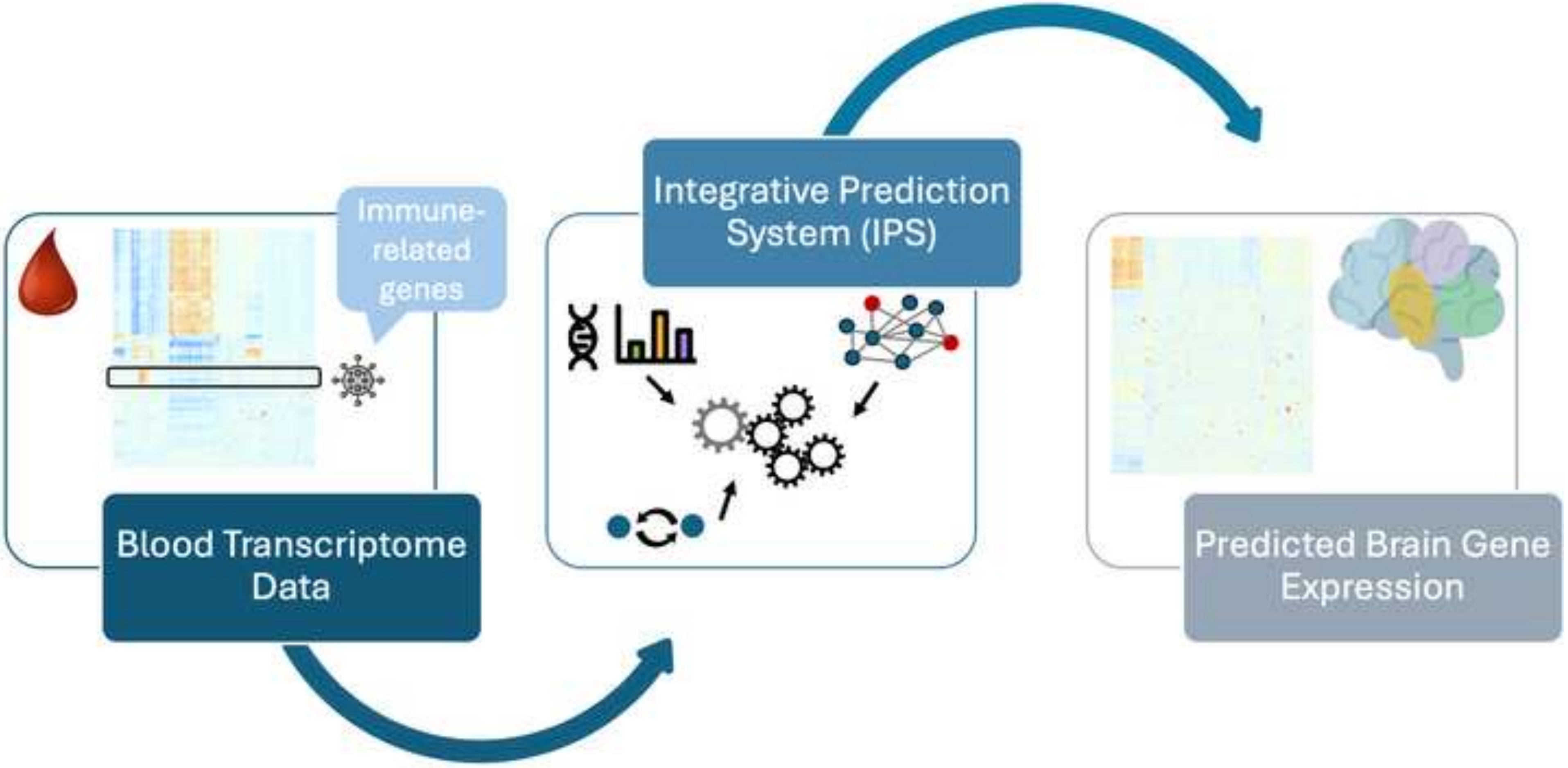

Supplement: giag058_GIGA-D-25-00434_revision_1 [file giag058_giga-d-25-00434_revision_1.pdf]
