# Supplementary material for: Leveraging machine learning and network biology approaches to predict brain gene expression from blood transcriptomes
Source: Gigascience. 2026 May 18;15:giag058. doi: 10.1093/gigascience/giag058 (PMC13201078; doi:10.1093/gigascience/giag058)
Supplement: giag058_Authors_Response_To_Reviewer_Comments_original_submission [file giag058_authors_response_to_reviewer_comments_original_submission.pdf]

Author's Response To Reviewer Comments

Responses to Reviewers' Comments

We sincerely appreciate the thoughtful comments from the reviewers, which provided us with valuable insights and opportunities to greatly improve the quality of the manuscript. We have revised the manuscript according to the reviewers' comments and provided a point-by-point response to each suggestion below.

Responses to the reviewers' comments are provided below with our responses given in blue.

Reviewer #1:

General comments:

In Bayrak et al's work, the authors build machine learning models to predict brain gene expression based on the paired blood data leveraging network biology and different feature selection method. Some efforts were made in the work; however, it consists of some claims with exaggeration and not supported by the contents. Substantial improvements would be needed for it to get published.

We thank the reviewer for their overall assessment of our work and for highlighting concerns regarding potential overstatement of certain claims. We have carefully reviewed the manuscript and revised the text to ensure that our conclusions are fully supported by the presented results. In particular, we have moderated language in the abstract and discussion sections and clarified the scope and limitations of our approach. We believe these revisions address the concerns raised.

Major Comments:

1. It seems the authors used a simple linear regression (and its variants) model for this whole framework. Is there a justification for that? Of course, sometimes simple model outperforms any of the fancy modern methods, but at least discussions should be added, or other methods should be evaluated/explored for comparison.

Response #1: We thank the reviewer for this important and constructive comment. The use of linear regression and elastic net models in our framework was a deliberate choice, guided by both prior literature and our own empirical evaluation.

First, prior studies on predicting tissue-specific gene expression from high-dimensional genomic features (e.g., BrainGenie, TEEBoT, and Bayesian ridge regression–based frameworks) have predominantly employed linear or regularized linear models. This is largely due to their robustness when sample sizes are limited and predictors are highly correlated.

Second, we evaluated multiple nonlinear machine learning methods, including random forest, support vector machines, and gradient boosting (XGBoost), using the same training and validation framework. As now shown in Supplementary Table 3, these more complex models did not provide consistent improvement in predictive performance compared to linear regression or elastic net. Notably, for moderate-to-high prediction thresholds (e.g., $r > 0.5$), linear and elastic models achieved comparable or better performance in terms of the number of well-predicted genes. Based on these results, we selected linear models for the main analyses to balance accuracy and interpretability.

Importantly, we would like to emphasize that the proposed framework is model-agnostic and flexible. While linear models performed well in the current setting, particularly at higher correlation thresholds, the pipeline can readily incorporate alternative modeling approaches depending on the research context. The primary contribution of our work is not tied to a specific modeling choice, but rather to the demonstration that gene-specific feature selection and model selection can improve predictive performance for individual

genes.

To address the reviewer’s concern, we have now included a comparison of these alternative methods in the Supplementary Table 3, and we have expanded the Discussion to explicitly comment on model choice. (Methods, page 7, lines 151-155; Results, page 12, lines 262-268; Discussion, pages 21-22, lines 497-504)

2. How is the generalizability of the models? Other than internal training/validation, is there any external validation on any of the models? On page 11, last paragraph: "To assess the predictive capacity of our models for brain expression of AD-related genes, we evaluated the prediction accuracy of the top 1,000 AD key drivers identified in postmortem parahippocampal gyrus (PHG) samples from the Mount Sinai Brain Bank (MSBB) AD cohort…", but this doesn't seem to be applying the model to an external dataset. If the models are built based on data from GTEx, one would expect the they would not be so informative in regard to disease state such as Alzheimer's disease. If the authors' intention is to evaluate the genes implicated in the disease, the models probably wouldn't perform so well since the GTEx data is essentially the comprehensive baseline for "normal" human gene expression.

Response #2: We thank the reviewer for raising this important point regarding model generalizability and external validation. We agree that independent external validation is the gold standard for assessing generalizability. However, at present, there are no publicly available datasets with matched blood and brain expression data in independent cohorts, which precludes direct external validation of our framework.

The analysis involving AD–related genes was not intended as an external validation of the models across datasets or disease states. Rather, it was designed to assess whether genes previously implicated in AD (which were previously identified independently in the MSBB cohort) are among those that can be reliably predicted by our models trained on GTEx data.

Consistent with the reviewer’s observation, our models are trained on GTEx samples representing largely non-diseased baseline expression, and we do not expect them to capture disease-specific expression changes. Instead, the AD-related gene analysis evaluates the baseline predictability of genes with known relevance to AD.

We have revised the manuscript to clarify this distinction. We also now emphasize the lack of external matched datasets as a limitation. (Results, page 15, lines 337-340, Discussion, page 22, lines 509-510)

3. How is age modeled in the whole framework? The authors claim that sex had been regressed out in the dataset prior to model, and on page 13 there is a section "Preservation of Age-Associated Expression Patterns in Predicted Gene Profiles", does it indicate that age is just left out in the models? In the section, they state that "We first identified highly predictable genes (CV r > 0.5)" (how many are there? It would be helpful to be specific) and computed the correlation before/after prediction. If the genes are highly correlated in the first place, r1/r2 would be intuitively correlated as well. I would argue many of the age-related changes are actually present within those genes the models didn't predict well, which contradict the conclusion "the model effectively preserves age-associated expression patterns". It would be helpful to compare the genes the model predicted well and poorly to see if age plays a role, or, to include age in the model in the first place to see if it makes any impact.

Response #3: We thank the reviewer for the thoughtful and detailed comments regarding age modeling and the interpretation of the age-associated expression analysis. We clarify that age was not regressed out prior to model training; only technical covariates and sex were adjusted for, as described in the Methods, subsection “Dataset”.

The analysis in the section “Preservation of Age-Associated Expression Patterns in Predicted Gene Profiles” was intended as a post hoc evaluation of whether age-related transcriptional signals present in the observed brain expression are retained in the predicted expression profiles, rather than as a test of whether the models explicitly model age effects.

In the hippocampus, 353 genes met the criterion of high predictability (CV r > 0.5). For these genes, we

computed the correlation between observed expression and age (r_1) and compared it to the correlation between predicted expression and age (r_2). This correspondence was moderate ($r = 0.496$), indicating that age-associated expression patterns are partially preserved for well-predicted genes.
We agree with the reviewer that preservation of age-associated effects should not be assumed for all genes. To address this, we extended the analysis by stratifying genes based on prediction accuracy. Among poorly predicted genes (CV $r<0.1$; N=536 genes), the correspondence between r_1 and r_2 was substantially weaker ($r = 0.195$). This comparison demonstrates that preservation of age-associated expression patterns depends on prediction quality and is not a trivial consequence of correlation structure alone.

We have revised the manuscript to explicitly report the number of genes in each group and to refine the interpretation of the results. The text now emphasizes that predicted expression preserves age-associated patterns for a subset of well-predicted genes, rather than across the entire transcriptome. We included the stratified analysis in Supplementary Table S7. (Results, page 17, lines 376-391)

4. In general, blood-brain cross-tissue gene expression prediction is weak, and the current models do not demonstrate strong or reliable performance. Therefore, the statement in the last paragraph on page 16 regarding the feasibility of using blood-based transcriptomic models as diagnostic or prognostic tools, substantially overstates what the current results support. At present, the models lack external validation and their performance in actual disease cohorts is unknown. The ROSMAP dataset, which contains paired blood and brain RNA-seq (albeit with some time lag), could serve as a valuable resource for assessing generalizability and helping determine whether these models can meaningfully predict brain expression in independent samples.

Response #4: We thank the reviewer for suggesting the use of the ROSMAP dataset to assess generalizability. In response, we applied the same prediction pipeline to ROSMAP using monocyte RNA-seq as predictors and dorsolateral prefrontal cortex expression as the outcome. While this does not constitute a formal external validation due to differences in cell type, cohort composition, and sample collection timing, it provides a useful comparison across datasets. We observed that fewer genes were predictable in ROSMAP than in GTEx across all thresholds; however, the overlap of top predicted genes was significantly greater than expected by chance. These results are presented in the newly added Supplementary Figure S8. (Methods, page 9, lines 183-187, Results, pages 20-21 lines 460-482)

We agree that external validation in an appropriately matched cohort would be valuable; however, at present, no such dataset is available. Accordingly, we have revised the Discussion to temper the claims regarding diagnostic or prognostic applications, emphasizing that these models are currently best suited for research use and that their performance in clinical cohorts remains to be established. (Discussion, page 22, lines 519-528)

5. How are the models transferable between different brain regions? There are some statistics on the percentage of genes for which each feature set performed best across different brain regions (Figure 4), have the authors tried to assess how one model trained from one region to predict related regions?

Response #5: We thank the reviewer for the suggestion regarding cross-region model transfer. In our framework, each gene in each brain region is modeled separately using blood gene expression as input. As a result, each model captures the blood-to-brain relationships and regulatory structure that are specific to that tissue.

While it is theoretically possible to apply a model trained in one brain region to predict expression in another, we anticipate performance would be limited due to region-specific differences in gene regulation, co-expression networks, and blood–brain correlations. Therefore, we focus on optimizing predictions for each tissue separately. We have clarified this point in the manuscript to emphasize that each model is intended for the specific brain region in which it was trained. (Results, page 15, lines 329-332)

Minor Comments:

1. The workflow chart (Figure 1) is too abstract. It would benefit the readers to have a more concrete description of the actual work done in the manuscript. e.g. the specific feature selection methods explored,

how final models are picked for evaluation, what kind of evaluations have been done etc. More texts in the picture would help.

Response #6: We thank the reviewer for this helpful suggestion. In the revised manuscript, we have substantially updated Figure 1 to provide a more concrete and detailed representation of the workflow. We believe the revised workflow now provides a clearer and more informative overview of the design and analysis pipeline.

2. BrainGENIE will need a citation and some introductions in the introduction section since it has been used in the manuscript for comparison.

Response #7: We thank the reviewer for the suggestion. We have revised the Introduction to provide a clearer description of BrainGENIE. (page 4, lines 63-67)

3. In the title "to Uncover Blood Biomarkers" suggests that the goal of the work is for feature section. However, the contents of the manuscript are more focused on model building and evaluation based on different feature section method, since at least two methods are based purely based on correlation. I would revise the title to reflect the facts.

Response 8: We thank the reviewer for this note. While the term “biomarker” in our current title refers to blood gene expression features that can predict brain expression, we agree that the manuscript primarily focuses on building and evaluating predictive models rather than validating biomarkers in clinical cohorts. To more accurately reflect the scope of the work, we propose revising the title to:
“Leveraging Machine Learning and Network Biology Approaches to Predict Brain Gene Expression from Blood Transcriptomes”

4. There is no legend for the heatmaps in Figure 6 & 7.

Response #9: We thank the reviewer for this comment. We have revised Figures 6 and 7 to include labeled legends for each heatmap.

Reviewer #2:

General comments:

The manuscript presents an integrative framework that combines machine learning and network biology to predict brain gene expression from blood transcriptomic data. The topic is important. However, several issues related to clarity, methodological description, and result presentation need to be addressed to improve the rigor and reproducibility of the study.

We thank the reviewer for recognizing the importance of our work and for the constructive feedback. In response, we have carefully revised the manuscript to improve the clarity of the methodology, providing additional details where necessary to facilitate reproducibility. We have also revised the presentation of results to improve readability and ensure that the key findings are clearly conveyed. We believe these revisions address the concerns raised and strengthen the overall quality of the manuscript.

Major Comments:

1. It lacks sufficient clarity in the description of the prediction models. The Methods section states that linear regression and elastic net models were used, but the exact formulation of the linear model is not clearly described. It is unclear:
What is the dependent variable and what features are included in the regression?
An explicit mathematical formulation of the regression model (e.g., an equation) should be provided to clearly illustrate how the features enter the model and how predictions are generated.

Response #10: We thank the reviewer for highlighting the need for clarity in model description. For each brain gene, we trained linear regression and elastic net models using normalized blood gene expression as

features. The dependent variable is the residualized expression of a single brain gene in a specific brain region, after regressing out sex and sequencing-related covariates. Linear regression models predict brain expression as a weighted sum of blood gene expression, while elastic net models include L1/L2 penalties to stabilize coefficient estimates in the presence of highly correlated predictors.

Predictions were generated for training and test sets, and performance was evaluated using Pearson correlation between predicted and observed expression. An explicit mathematical formulation of both models has been added to the Methods section, and all code is available via GitLab. We believe this revision fully clarifies the modeling framework for readers. (Methods, page 8, lines 164-178)

2. While the manuscript reports that IPS outperforms BrainGENIE, the comparison focuses primarily on the number of predicted genes. It would be more informative to also: Show the overall distribution of average cross-validation correlation coefficients (CV r) for IPS versus BrainGENIE. Provide a direct comparison of prediction accuracy distributions rather than only gene counts above a threshold.

Response #11: We thank the reviewer for this helpful suggestion. In response, we have now examined the full distribution of average cross-validation correlation coefficients (CV r) for both IPS and BrainGENIE. As background, BrainGENIE uses the top 40 PCs with a linear modeling framework to predict all genes, which is one of the approaches we already include in our integrated framework. To ensure a fair and controlled comparison, rather than rerunning BrainGENIE, we compared IPS directly to a linear model trained on the top 40 PCs across all genes using identical training/testing splits. Specifically, we generated density plots showing the overall distribution of prediction accuracies across all genes and performed a direct comparison of the two methods' CV r distributions. These results are now presented in newly added Figure 3B. The figure demonstrates that IPS shows a consistently shifted distribution toward higher CV r values relative to BrainGENIE, supporting the conclusion that IPS achieves improved prediction performance beyond differences in gene counts above threshold. (Results, page 11, lines 237-245)

3. The manuscript compares IPS mainly with BrainGENIE. However, previous methods such as TEEBoT (ref. 13) and Bayesian ridge regression-based approaches (ref. 14) are cited but not systematically benchmarked. A more comprehensive comparison with these methods would strengthen the claims of improved performance.

Response #12: We thank the reviewer for this important suggestion. We agree that benchmarking against previously published approaches strengthens the evaluation of IPS.
TEEBoT and B-GEX were both trained on earlier versions of GTEx that included substantially fewer samples than the version used in our study. Because these methods were trained on earlier releases, direct retraining under identical conditions was not feasible.
Available TEEBoT results were limited to two brain regions (caudate and cerebellum), and B-GEX results were available only for cerebellum. To enable comparison, we evaluated the number of genes predicted at increasing correlation thresholds ($r = 0.0–0.9$) and compared these distributions to TEEBoT. For cerebellum, B-GEX reported a mean gene-level correlation of 0.044, which was lower than that achieved by our integrated IPS approach. Across available regions and thresholds, IPS consistently predicted a greater number of genes at comparable or higher accuracy levels than both TEEBoT and B-GEX.

We have now incorporated this comparison and its limitations into the Results section. (Results, pages 11-12, pages 246-261).

4. It remains unclear whether prediction accuracy is influenced by gene expression abundance in blood or brain. Specifically: Are highly expressed genes in blood generally better predicted? Is there a correlation between gene expression level and prediction performance? An explicit analysis addressing this potential confounding factor would improve the biological interpretation of the results.

Response #13: We thank the reviewer for raising this important point. To evaluate whether prediction performance is confounded by gene expression abundance, we explicitly examined the relationship between gene expression level and prediction accuracy. For each brain region, we computed the correlation between gene expression abundance and prediction accuracy. This analysis was performed separately using (i) gene expression levels in blood (Supp Figure S6) and (ii) gene expression levels in brain tissue

(Supp Figure S7). Across all regions, the correlation between abundance and prediction accuracy was negligible ($|r| < 0.1$). This indicates that prediction performance is not systematically driven by highly expressed genes. The top-performing genes showed that they exhibit moderate expression levels in both blood and brain tissues. We have included this new analysis in the Results section. (Results, page 19, lines 450-459).

Minor:

1. While Figure 2A shows the number of paired blood-brain samples across tissues, the manuscript does not clearly state: The exact number of samples used for each brain region in model training. Whether the same individuals are used across different brain regions. A comprehensive summary table describing sample size, tissue type, preservation method (e.g., PAXgene vs. frozen), and relevant covariates should be provided as a supplementary table.

Response #14: We thank the reviewer for this suggestion. We have added a comprehensive summary table (Supplementary Table S1) showing, for each brain tissue, the preservation method, the number of individuals with paired whole blood samples, mean age, and sex distribution).

2. Figure 2A does not include a clear y-axis label. The metric being displayed (e.g., number of samples or individuals) should be explicitly labeled to avoid ambiguity.

Response #15: We thank the reviewer for this comment. We have revised Figure 2A to include clear axis labels.

3. Figure S1 presents prediction performance across different feature combinations, but the details of these combinations are not adequately explained in the main text or figure legend. The authors should clearly describe: What each feature combination represents.

Response #16: We thank the reviewer for this suggestion. To clarify the feature combinations used in the prediction analysis, we have now included a comprehensive supplementary table (Supplementary Table S6) that lists, for each correlation cutoff and brain region, the number of genes predicted using 1–5 top methods combined. This table also specifies the combined top methods, allowing readers to see exactly which features were included in each set. (Results, page 14, lines 310-328)

4. Figures 6B and 6C present pathway and GO enrichment heatmaps, but no color scale is shown. A color bar indicating the meaning of the colors (e.g., -log10 P value or adjusted P value) should be added.

Response #17: We thank the reviewer for this comment. We have revised Figures 6 and 7 to include labeled legends for each heatmap. The heatmaps are plotted using -log10(P-value). We have also edited the legends for clarity.

em Editorial Manager®

Cookie Preference Center

We use cookies which are necessary to make our site work. We may also use additional cookies to analyze, improve and personalize our content and your digital experience. For more information, see our Cookie Policy.

You may choose not to allow some types of cookies. However, blocking some types may impact your experience of our site and the services we are able to offer. See the different category headings below to find out more or change your settings.

Allow all

Manage Consent Preferences

Strictly Necessary Cookies

Always active

These cookies are necessary for the website to function and cannot be switched off in our systems. They are usually only set in response to actions made by you which amount to a request for services, such as setting your privacy preferences, logging in or filling in forms. You can set your browser to block or alert you about these cookies, but some parts of the site will not then work.

Functional Cookies

These cookies enable the website to provide enhanced functionality and personalisation. They may be set by us or by third party providers whose services we have added to our pages. If you do not allow these cookies then some or all of these services may not function properly.

Performance Cookies

These cookies allow us to count visits and traffic sources so we can measure and improve the performance of our site. They help us to know which pages are the most and least popular and see how visitors move around the site.

Targeting Cookies

These cookies may be set through our site by our advertising partners. They may be used by those companies to build a profile of your interests and show you relevant adverts on other sites. If you do not allow these cookies, you will experience less targeted advertising.

Cookie List

Clear

Apply Cancel
Consent Leg.Interest

Confirm my choices

Power
