# Supplementary material for: Leveraging machine learning and network biology approaches to predict brain gene expression from blood transcriptomes
Source: Gigascience. 2026 May 18;15:giag058. doi: 10.1093/gigascience/giag058 (PMC13201078; doi:10.1093/gigascience/giag058)
Supplement: giag058_Reviewer_1_Report_original_submission — Reviewer 1 -- 12/9/2025 [file giag058_reviewer_1_report_original_submission.pdf]

Reviewer Report

Title: Leveraging Machine Learning and Network Biology Approaches to Predict Brain Gene Expression from Blood Transcriptomes

Version: Original Submission **Date:** 12/9/2025

Reviewer name: Reviewer 1

Reviewer Comments to Author:

In Bayrak et al's work, the authors build machine learning models to predict brain gene expression based on the paired blood data leveraging network biology and different feature selection method. Some efforts were made in the work; however, it consists of some claims with exaggeration and not supported by the contents. Substantial improvements would be needed for it to get published.

Major:

1. It seems the authors used a simple linear regression (and its variants) model for this whole framework. Is there a justification for that? Of course, sometimes simple model outperforms any of the fancy modern methods, but at least discussions should be added, or other methods should be evaluated/explored for comparison.
2. How is the generalizability of the models? Other than internal training/validation, is there any external validation on any of the models? On page 11, last paragraph: "To assess the predictive capacity of our models for brain expression of AD-related genes, we evaluated the prediction accuracy of the top 1,000 AD key drivers identified in postmortem parahippocampal gyrus (PHG) samples from the Mount Sinai Brain Bank (MSBB) AD cohort…", but this doesn't seem to be applying the model to an external dataset. If the models are built based on data from GTEx, one would expect the they would not be so informative in regard to disease state such as Alzheimer's disease. If the authors' intention is to evaluate the genes implicated in the disease, the models probably wouldn't perform so well since the GTEx data is essentially the comprehensive baseline for "normal" human gene expression.
3. How is age modeled in the whole framework? The authors claim that sex had been regressed out in the dataset prior to model, and on page 13 there is a section "Preservation of Age-Associated Expression Patterns in Predicted Gene Profiles", does it indicate that age is just left out in the models? In the section, they state that "We first identified highly predictable genes (CV $r > 0.5$)" (how many are there? It would be helpful to be specific) and computed the correlation before/after prediction. If the genes are highly correlated in the first place, r1/r2 would be intuitively correlated as well. I would argue many of the age-related changes are actually present within those genes the models didn't predict well, which contradict the conclusion "the model effectively preserves age-associated expression patterns". It would be helpful to compare the genes the model predicted well and poorly to see if age plays a role, or, to include age in the model in the first place to see if it makes any impact.
4. In general, blood-brain cross-tissue gene expression prediction is weak, and the current models do not demonstrate strong or reliable performance. Therefore, the statement in the last paragraph on page 16 regarding the feasibility of using blood-based transcriptomic models as diagnostic or prognostic tools, substantially overstates what the current results support. At present, the models lack external validation

and their performance in actual disease cohorts is unknown. The ROSMAP dataset, which contains paired blood and brain RNA-seq (albeit with some time lag), could serve as a valuable resource for assessing generalizability and helping determine whether these models can meaningfully predict brain expression in independent samples.
5. How are the models transferable between different brain regions? There are some statistics on the percentage of genes for which each feature set performed best across different brain regions (Figure 4), have the authors tried to assess how one model trained from one region to predict related regions?
Minor:
1. The workflow chart (Figure 1) is too abstract. It would benefit the readers to have a more concrete description of the actual work done in the manuscript. e.g. the specific feature selection methods explored, how final models are picked for evaluation, what kind of evaluations have been done etc. More texts in the picture would help.
2. BrainGENIE will need a citation and some introductions in the introduction section since it has been used in the manuscript for comparison.
3. In the title "to Uncover Blood Biomarkers" suggests that the goal of the work is for feature section. However, the contents of the manuscript are more focused on model building and evaluation based on different feature section method, since at least two methods are based purely based on correlation. I would revise the title to reflect the facts.
4. There is no legend for the heatmaps in Figure 6 & 7.

Methods

Are the methods appropriate to the aims of the study, are they well described, and are necessary controls included? Choose an item.

Conclusions

Are the conclusions adequately supported by the data shown? Choose an item.

Reporting Standards

Does the manuscript adhere to the journal’s guidelines on minimum standards of reporting? Choose an item.

Choose an item.

Statistics

Are you able to assess all statistics in the manuscript, including the appropriateness of statistical tests used? Choose an item.

Quality of Written English

Please indicate the quality of language in the manuscript: Choose an item.

Declaration of Competing Interests

Please complete a declaration of competing interests, considering the following questions:

- Have you in the past five years received reimbursements, fees, funding, or salary from an organisation that may in any way gain or lose financially from the publication of this manuscript, either now or in the future?
- Do you hold any stocks or shares in an organisation that may in any way gain or lose financially from the publication of this manuscript, either now or in the future?
- Do you hold or are you currently applying for any patents relating to the content of the manuscript?
- Have you received reimbursements, fees, funding, or salary from an organization that holds or has applied for patents relating to the content of the manuscript?
- Do you have any other financial competing interests?
- Do you have any non-financial competing interests in relation to this paper?

If you can answer no to all of the above, write 'I declare that I have no competing interests' below. If your reply is yes to any, please give details below.

I declare that I have no competing interests

I agree to the open peer review policy of the journal. I understand that my name will be included on my report to the authors and, if the manuscript is accepted for publication, my named report including any attachments I upload will be posted on the website along with the authors' responses. I agree for my report to be made available under an Open Access Creative Commons CC-BY license (http://creativecommons.org/licenses/by/4.0/). I understand that any comments which I do not wish to be included in my named report can be included as confidential comments to the editors, which will not be published.

Choose an item.

To further support our reviewers, we have joined with Publons, where you can gain additional credit to further highlight your hard work (see: https://publons.com/journal/530/gigascience). On publication of this paper, your review will be automatically added to Publons, you can then choose whether or not to claim your Publons credit. I understand this statement.

Yes Choose an item.
