# Supplementary material for: Leveraging machine learning and network biology approaches to predict brain gene expression from blood transcriptomes
Source: Gigascience. 2026 May 18;15:giag058. doi: 10.1093/gigascience/giag058 (PMC13201078; doi:10.1093/gigascience/giag058)
Supplement: giag058_Reviewer_2_Report_original_submission — Reviewer 2 -- 1/11/2026 [file giag058_reviewer_2_report_original_submission.pdf]

Reviewer Report

Title: Leveraging Machine Learning and Network Biology Approaches to Predict Brain Gene Expression from Blood Transcriptomes

Version: Original Submission **Date:** 1/11/2026

Reviewer name: Reviewer 2

Reviewer Comments to Author:

The manuscript presents an integrative framework that combines machine learning and network biology to predict brain gene expression from blood transcriptomic data. The topic is important. However, several issues related to clarity, methodological description, and result presentation need to be addressed to improve the rigor and reproducibility of the study.

Major Comments:

1. It lacks sufficient clarity in the description of the prediction models. The Methods section states that linear regression and elastic net models were used, but the exact formulation of the linear model is not clearly described. It is unclear:

What is the dependent variable and what features are included in the regression?

An explicit mathematical formulation of the regression model (e.g., an equation) should be provided to clearly illustrate how the features enter the model and how predictions are generated.

2. While the manuscript reports that IPS outperforms BrainGENIE, the comparison focuses primarily on the number of predicted genes. It would be more informative to also: Show the overall distribution of average cross-validation correlation coefficients (CV r) for IPS versus BrainGENIE. Provide a direct comparison of prediction accuracy distributions rather than only gene counts above a threshold.

3. The manuscript compares IPS mainly with BrainGENIE. However, previous methods such as TEEBoT (ref. 13) and Bayesian ridge regression-based approaches (ref. 14) are cited but not systematically benchmarked. A more comprehensive comparison with these methods would strengthen the claims of improved performance.

4. It remains unclear whether prediction accuracy is influenced by gene expression abundance in blood or brain. Specifically: Are highly expressed genes in blood generally better predicted? Is there a correlation between gene expression level and prediction performance? An explicit analysis addressing this potential confounding factor would improve the biological interpretation of the results.

Minor:

1. While Figure 2A shows the number of paired blood-brain samples across tissues, the manuscript does not clearly state: The exact number of samples used for each brain region in model training. Whether the same individuals are used across different brain regions. A comprehensive summary table describing sample size, tissue type, preservation method (e.g., PAXgene vs. frozen), and relevant covariates should be provided as a supplementary table.

2. Figure 2A does not include a clear y-axis label. The metric being displayed (e.g., number of samples or individuals) should be explicitly labeled to avoid ambiguity.

3. Figure S1 presents prediction performance across different feature combinations, but the details of

these combinations are not adequately explained in the main text or figure legend. The authors should clearly describe: What each feature combination represents.
4. Figures 6B and 6C present pathway and GO enrichment heatmaps, but no color scale is shown. A color bar indicating the meaning of the colors (e.g., -log10 P value or adjusted P value) should be added.

Methods

Are the methods appropriate to the aims of the study, are they well described, and are necessary controls included? Choose an item.

Conclusions

Are the conclusions adequately supported by the data shown? Choose an item.

Reporting Standards

Does the manuscript adhere to the journal's guidelines on minimum standards of reporting? Choose an item.

Choose an item.

Statistics

Are you able to assess all statistics in the manuscript, including the appropriateness of statistical tests used? Choose an item.

Quality of Written English

Please indicate the quality of language in the manuscript: Choose an item.

Declaration of Competing Interests

Please complete a declaration of competing interests, considering the following questions:

- Have you in the past five years received reimbursements, fees, funding, or salary from an organisation that may in any way gain or lose financially from the publication of this manuscript, either now or in the future?
- Do you hold any stocks or shares in an organisation that may in any way gain or lose financially from the publication of this manuscript, either now or in the future?
- Do you hold or are you currently applying for any patents relating to the content of the manuscript?
- Have you received reimbursements, fees, funding, or salary from an organization that holds or has applied for patents relating to the content of the manuscript?
- Do you have any other financial competing interests?

- Do you have any non-financial competing interests in relation to this paper?

If you can answer no to all of the above, write 'I declare that I have no competing interests' below. If your reply is yes to any, please give details below.

No

I agree to the open peer review policy of the journal. I understand that my name will be included on my report to the authors and, if the manuscript is accepted for publication, my named report including any attachments I upload will be posted on the website along with the authors' responses. I agree for my report to be made available under an Open Access Creative Commons CC-BY license (http://creativecommons.org/licenses/by/4.0/). I understand that any comments which I do not wish to be included in my named report can be included as confidential comments to the editors, which will not be published.

Choose an item.

To further support our reviewers, we have joined with Publons, where you can gain additional credit to further highlight your hard work (see: https://publons.com/journal/530/gigascience). On publication of this paper, your review will be automatically added to Publons, you can then choose whether or not to claim your Publons credit. I understand this statement.

Yes Choose an item.
